# Heterochiral and Heterotypic Self-Assembly of Intrinsically Disordered Peptides Confers Peptide Supercoils with Exceptional Proteolytic Stability

**DOI:** 10.1021/jacs.5c13598

**Published:** 2025-10-21

**Authors:** Yuchen Qiao, Myeonggon Park, Matthew Chu, Grace Wu, Ruipeng Guo, Chen Liu, Hongjian He, Tongyu Li, Lei Tian, Xixiang Zhang, W. Benjamin Rogers, Bing Xu

**Affiliations:** † Department of Chemistry, 8244 Brandeis University, 415 South St., Waltham, Massachusetts 02453, United States; ‡ Martin A. Fisher School of Physics, Brandeis University, Waltham, Massachusetts 02453, United States; § Department of Electrical and Computer Engineering, 1846 Boston University, Boston, Massachusetts 02215, United States; ∥ Physical Science and Engineering Division, 127355 King Abdullah University of Science and Technology, Thuwal, 23955-6900, Saudi Arabia

## Abstract

Chirality has received extensive exploration in homotypic supramolecular assemblies; however, few heterotypic peptide assemblies employ heterochirality, especially in the context of intrinsically disordered peptides (IDPs). In this work, we show that heterochiral, heterotypic assemblies of IDPs unexpectedly form supercoils of nanofibers. Specifically, conjugating an aromatic motif to IDPs with opposite charge and chirality results in positively and negatively charged IDPs that form supercoils when mixed in a 2:1 ratio. These supercoils significantly modulate the enzymatic stability of l-peptides: they prevent the proteolysis of l-peptides when the d-peptide to l-peptide ratio is 2:1 but promote it when the ratio is 1:2. Moreover, the formation of supercoils enhances the stability of post-translational modification, phosphotyrosine, against a powerful phosphatase. This work presents the first case of heterochiral and heterotypic assemblies of IDPs. It offers a novel and facile approach for designing supramolecular materials made of IDPs with tunable enzymatic stability. These materials promise applications in a variety of in vivo settings, particularly when efficacy depends on enzymatic stability of IDPs.

## Graphical abstract

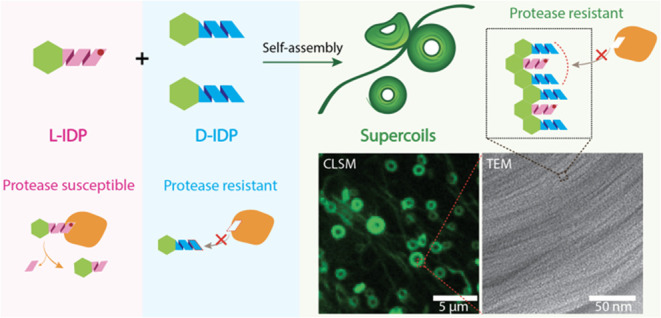

## Introduction

This article reports that heterochiral, heterotypic assemblies of intrinsically disordered peptides (IDPs) form supercoils, which confer remarkable proteolytic resistance to l-peptides. IDPs are often derived from naturally occurring intrinsically disordered proteins or designed de novo to mimic functional motifs involved in cellular signaling, molecular recognition, and regulatory pathways.^1–4^ IDPs lack a stable three-dimensional structure under physiological conditions and exhibit disorder even in the solid state,^5,6^ a feature that defines intrinsically disordered regions in proteins. Given their inherent flexibility and ability to engage in high-specificity yet low-affinity interactions, IDPs hold great promise as drug candidates, particularly in targeting protein–protein interactions and modulating intracellular signaling pathways. Their unique conformational plasticity allows them to engage in dynamic interactions with multiple targets, often making them ideal for modulating complex signaling pathways in diseases such as cancer, neurodegeneration, and infectious diseases. For example, several functional IDPs are emerging as promising drug candidates, such as HIV-derived TAT (Trans-Activator of Transcription) peptide^7^ that is widely used for drug delivery due to its ability to cross biological membranes, LL-37,^8,9^ an antimicrobial peptide (AMP) that functions via disorder-to-order transitions when interacting with bacterial membranes, thymosin α1 with FDA-approval for certain immunodeficiencies,^10^ and a C-terminal domain of human IL-10, named IT9302 that induces monocyte differentiation into TGF-β-producing tolerogenic dendritic cells.^11^ De novo peptides have induced liquid–liquid phase separation of α-synuclein.^12^ LEA-like peptides can tune liquid–liquid phase separation and prevent aggregation.^13^ These developments suggest that the inherent flexibility of IDPs, which allows dynamic interactions without rigid structures,^14^ holds untapped potential for rational peptide drug design.

However, a major challenge limiting the therapeutic application of IDPs is their susceptibility to rapid proteolytic degradation, which significantly shortens their half-life in vivo. Enhancing proteolytic stability is therefore critical for IDP-based drug development, especially for long-term release. Common strategies to improve peptide stability include sequence modifications such as *N*-methylation,^15^ d-amino acid substitution,^16–21^ cyclization,^22–24^ and conjugation with polyethylene glycol (PEG)^25^ or lipids.^26–28^ A well-known recent example is glucagon-like peptide-1 (GLP-1),^29^ a peptide hormone used for diabetes treatment, which has been engineered with fatty acid conjugation (e.g., liraglutide^30^) or backbone modifications (e.g., semaglutide^31^) to resist degradation by dipeptidyl peptidase-4 (DPP-4). Beyond these conventional approaches, emerging strategies seek to actively modulate proteolytic degradation for precise control of peptide activity. For instance, supramolecular glycosylation has been shown to accelerate proteolytic degradation of peptide nanofibrils,^32^ highlighting a novel method to fine-tune peptide stability through glycan-mediated structural remodeling. Ryu et al. reported that intramitochondrial self-assembly overcomes intracellular enzymatic degradation of l-peptides.^33^ Despite these advances, each approach still has limitations. For example, pegylation may be limited by the immunogenicity of PEG,^34,35^ lipidation likely increases the difficulty of purification, and backbone modification could result in a reduction in activity. Thus, there is still a need to explore new approaches, especially in the context of IDPs, where conformational flexibility favors enzymatic proteolysis.^36,37^

Our recent study showed that a C-terminal SRC-kinase–derived motif fused to a tripeptide formed nanofibers with disordered peripheral regions.^6^ These assemblies also exhibited reduced protein adsorption, suggesting that supramolecular assembly can shield peptide backbones from proteases, thereby potentially enhancing proteolytic resistance. This observation aligns with the notion of context-dependent resistance to proteolysis of intrinsically disordered proteins.^38^ Thus, we decided to examine whether d-amino acid–based IDPs can modulate the enzymatic stability of l-amino acid–based IDPs. In addition to testing the proteolytic stability of l-IDPs, we also sought to evaluate the stability of phosphopeptides against phosphatases, as reversible phosphorylation, a form of post-translational modification, is commonly associated with intrinsically disordered regions of proteins.^39,40^ Therefore, we generated a pyrene-conjugated, negatively charged phosphohexapeptide, Pyn-(l)-EEEEE_p_Y (**1**), and a pyrene-conjugated, positively charged hexapeptide, Pyn-(d)-kkkkkk (**2**). We selected these sequences because our previous study demonstrated that their l-enantiomers (Pyn-(l)-EEEEEY (**5**) and Pyn-(l)-KKKKKK (**4**)) can self-assemble to form nanofibers that retain intrinsic disorder.^5^ As a stereochemical control, we also generated Pyn-(d)-eeeee_p_y (**3**) and a pyrene-conjugated, positively charged hexapeptide, Pyn-(l)-KKKKKK (**4**) (Figure 1). To our surprise, we found that mixing Pyn-(l)-EEEEE_p_Y (**1**) with two equivalents of Pyn-(d)-kkkkkk (**2**) unexpectedly results in the formation of a supercoiled structure. A similar assembly occurs when mixing Pyn-(d)-eeeee_p_y (**3**) with two equivalents of Pyn-(l)-KKKKKK (**4**), confirming the generality of this heterochiral design. When the d-peptide to L-peptide ratio is 2:1, the resulting tightly integrated supercoils confer over 360-fold resistance to proteolysis (in the presence of proteinase K, a powerful protease) compared to l-IDPs alone. In these supercoils, two d-IDPs protect one l-IDP from degradation, whereas reversing the ratio (one d-IDP with two l-IDPs) accelerates l-IDP breakdown. Moreover, heterotypic assemblies stabilize phosphotyrosines, regardless of the peptide chirality, against alkaline phosphatase (ALP), increasing their stability by over 3 orders of magnitude. These results illustrate a supramolecular approach for boosting the enzyme stability of l-IDPs. Such advances not only provide a means to improve peptide drug stability but also open new avenues for designing tunable biomaterials with controlled degradation profiles for diverse therapeutic applications.

**Figure 1:**
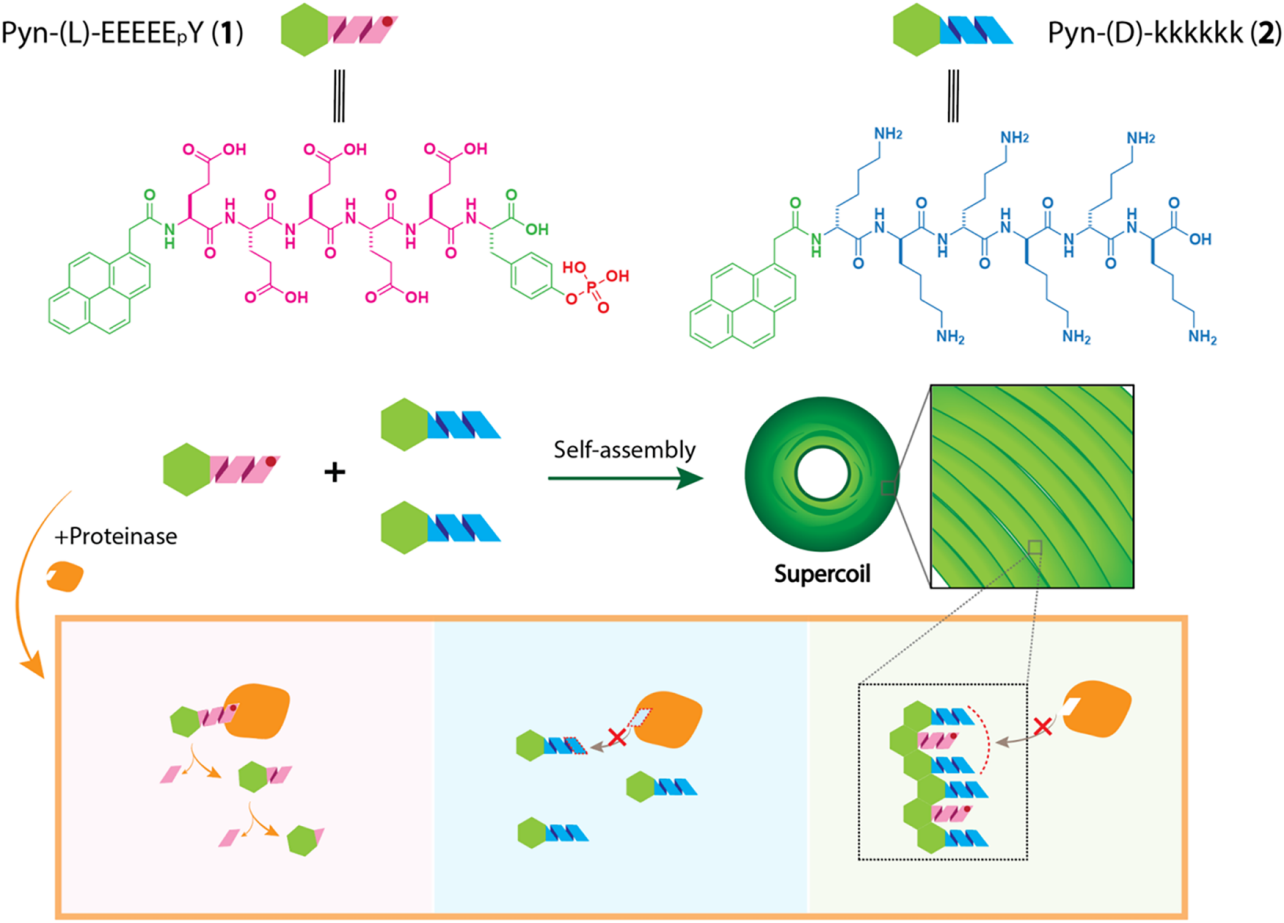
Molecular structures of the investigated IDPs and the proposed mechanism by which supercoil formation boosts the proteolytic stability of l-peptides.

## Results and Discussion

### Molecular Design and Synthesis

The negatively charged peptide sequence EEEEEY is derived from intrinsically disordered regions (IDRs) found in known proteins.^41–43^ Positively charged sequence KKKKKK is commonly present in disordered regions of various human proteins.^44–46^ To enable fluorescence monitoring and promote self-assembly via π–π stacking, a pyrene group was conjugated to the N-terminus of each peptide sequence. To mimic a common post-translational modification observed in intrinsically disordered regions (IDRs), we replaced the tyrosine residue in Pyn-(l)-EEEEEY (**5**) with a phosphorylated tyrosine to generate Pyn-(l)-EEEEE_p_Y (**1**). We synthesized the d-enantiomeric Pyn-(d)-eeeee_p_y (**3**), the phosphorylated analog of the dephosphorylated control Pyn-(d)-eeeeey (**6**). The positively charged peptide Pyn-(l)-KKKKKK (**4**) served as the primary binding partner for the heterotypic assembly studies. Its enantiomeric analog, Pyn-(d)-kkkkkk (**2**), is also a control. To evaluate the impact of sequence length and charge density, we prepared truncated variants of each type. For the negatively charged series, we included Pyn-(l)-EEE_p_Y (**7**) and Pyn-(l)-EEEE_p_Y (**8**). For the positively charged series, the truncated l-lysine analogs consisted of Pyn-(l)-KKK (**9**), Pyn-(l)-KKKK (**10**), and Pyn-(l)-KKKKK (**11**), while the d-form analogs included Pyn-(d)-kkk (**12**), Pyn-(d)-kkkk (**13**), and Pyn-(d)-kkkkk (**14**) (Figure S1).

We synthesized compounds **1**–**14** using conventional Fmoc solid-phase peptide synthesis. Briefly, 2-Cl-trityl chloride resin was swollen in methylene chloride (DCM) for 5 min before loading the first amino acid using *N*,*N*-diisopropylethylamine (DIPEA) in DCM overnight. We applied a capping solution (DCM:MeOH:DIPEA = 17:2:1) for 30 min and used 20% piperidine in dimethylformamide (DMF) for another 30 min to deprotect the peptides. We performed the subsequent amino acid couplings using HBTU, HOBt, and DIPEA for 2 h, washing with DMF between the steps. In the final coupling step, 1-pyreneacetic acid was added along with HBTU and DIPEA in the specified equivalents and allowed to react overnight. Peptides were cleaved with trifluoroacetic acid (TFA) for 1 h, then concentrated, precipitated in ethyl ether, and purified by HPLC. We obtained final products in multimilligram quantities with excellent yields, as verified by LC/MS (Figures S38–S51).

### Self-Assembly

#### Assembly Behavior of Each Peptide

Based on the critical aggregation concentrations (CACs) determined by using pyrene fluorescence^47^ (Figure S2), all peptides showed CAC values between 300–500 μM, indicating good aqueous solubility. This observation is consistent with the fact that these disordered peptides bear multiple charges. Among the negatively charged IDPs, the phosphorylated peptides Pyn-(l)-EEEEE_p_Y (**1**) and Pyn-(d)-eeeee_p_y (**3**) showed lower excimer emission at 5 mM than their dephosphorylated analogs, suggesting increased solubility upon phosphorylation. For peptides containing phosphotyrosines, shorter glutamic acid sequences displayed stronger excimer peaks, indicating reduced solubility with fewer acidic residues. Among the positively charged IDPs, reduced lysine content increased excimer emission at 5 mM, across both d- and l-forms. This result indicates that higher lysine content enhances hydrophilicity and reduces aggregation.

To examine the morphology of each peptide, we selected a concentration of 500 μM—slightly above the CACs—to allow for aggregation while maintaining solubility. TEM analysis showed that negatively charged peptides formed nanoparticles (Figure S3); positively charged peptides, however, formed nanosheets and micelles, without exhibiting ordered nanostructures (Figure S4).

To further characterize the morphology of the peptide assemblies, we performed confocal laser scanning microscopy (CLSM) on each sample. Although excitation at 405 nm is suboptimal for pyrene, we collected emission spectra between 420 to 600 nm—covering the excimer range—to maximize signal collection without introducing additional fluorophores. Consistent with TEM data, negatively charged peptides showed minimal fluorescence, while positively charged peptides formed large aggregates with strong fluorescence signals at neutral pH, particularly those containing 3–5 lysines (Figure S16). These CLSM results agree with TEM findings, suggesting that the bright aggregates correspond to aligned pyrene-capped nanosheets.

#### Heterochiral and Heterotypic Mixtures Forming Fibrillar Supercoils

In multicomponent systems that form ordered structures, both the molar ratio and the concentration of each component play critical roles.^48,49^ In our recent work, we found that heterotypic mixtures of oppositely charged IDPs could form ordered nanofiber structures. One-third of the peptides in the nanofibers remained partially disordered, indicating that intrinsic disorder is retained within the assembled nanofibers.^5^ Building on this observation, we systematically investigated the morphologies formed by mixing positively and negatively charged IDPs at varying stoichiometric ratios. Pyn-(l)-EEEEE_p_Y (**1**) or Pyn-(d)-eeeee_p_y (**3**) alone formed disordered nanoparticles; mixing **1** or **3** with oppositely charged Pyn-(l)-KKKKKK (**4**) or Pyn-(d)-kkkkkk (**2**), however, resulted in the formation of ordered structures (Figures S5–S6).

In homochiral mixtures, increasing the ratio of positively charged component led to a gradual transition from nanoparticles to nanofibers. Specifically, mixing Pyn-(l)-EEEEE_p_Y (**1**) with Pyn-(l)-KKKKKK (**4**) at a 1:0.5 ratio led to the appearance of nanofibers emerging from nanoparticle backgrounds. At a 1:1 ratio, the proportion of nanofibers increased significantly, and at a 1:2 ratio, the fibers bundled into thicker structures. d-peptide homochiral mixtures exhibited a similar progression, confirming consistent assembly behavior across enantiomeric forms. Homochiral mixtures of the nonphosphorylated analogs, such as Pyn-(l)-EEEEEY (**5**) with Pyn-(l)-KKKKKK (**4**), also resulted in fiber formation, consistent with our prior findings.^5^

Heterochiral mixtures, in contrast, yielded distinct and more complex morphologies. At a 1:0.5 ratio of Pyn-(l)-EEEEE_p_Y (**1**) and Pyn-(d)-kkkkkk (**2**), thick, helical fibers and fiber bundles dominated. At a 1:1 ratio, some of the fibers exhibited ring-like curvature, and at a 1:2 ratio, these curved fibers assembled into well-defined donut-shaped supercoils (Figures 2A and S5–S6). The enantiomeric combination of Pyn-(d)-eeeee_p_y (**3**) and Pyn-(l)-KKKKKK (**4**) exhibited a comparable trend, underscoring the generality of the heterochirality-driven assembly behavior. Heterochiral mixtures of the nonphosphorylated analogs, such as Pyn-(l)-EEEEEY (**5**) with Pyn-(d)-kkkkkk (**2**), or their enantiomers, however, exhibited few such donut-like structures. This observation suggests that tyrosine phosphorylation plays a key role in promoting the curvature required for supercoil assembly (Figures S7–S8), likely due to enhanced electrostatic interactions between the phosphate group and lysine residues.^50^

**Figure 2:**
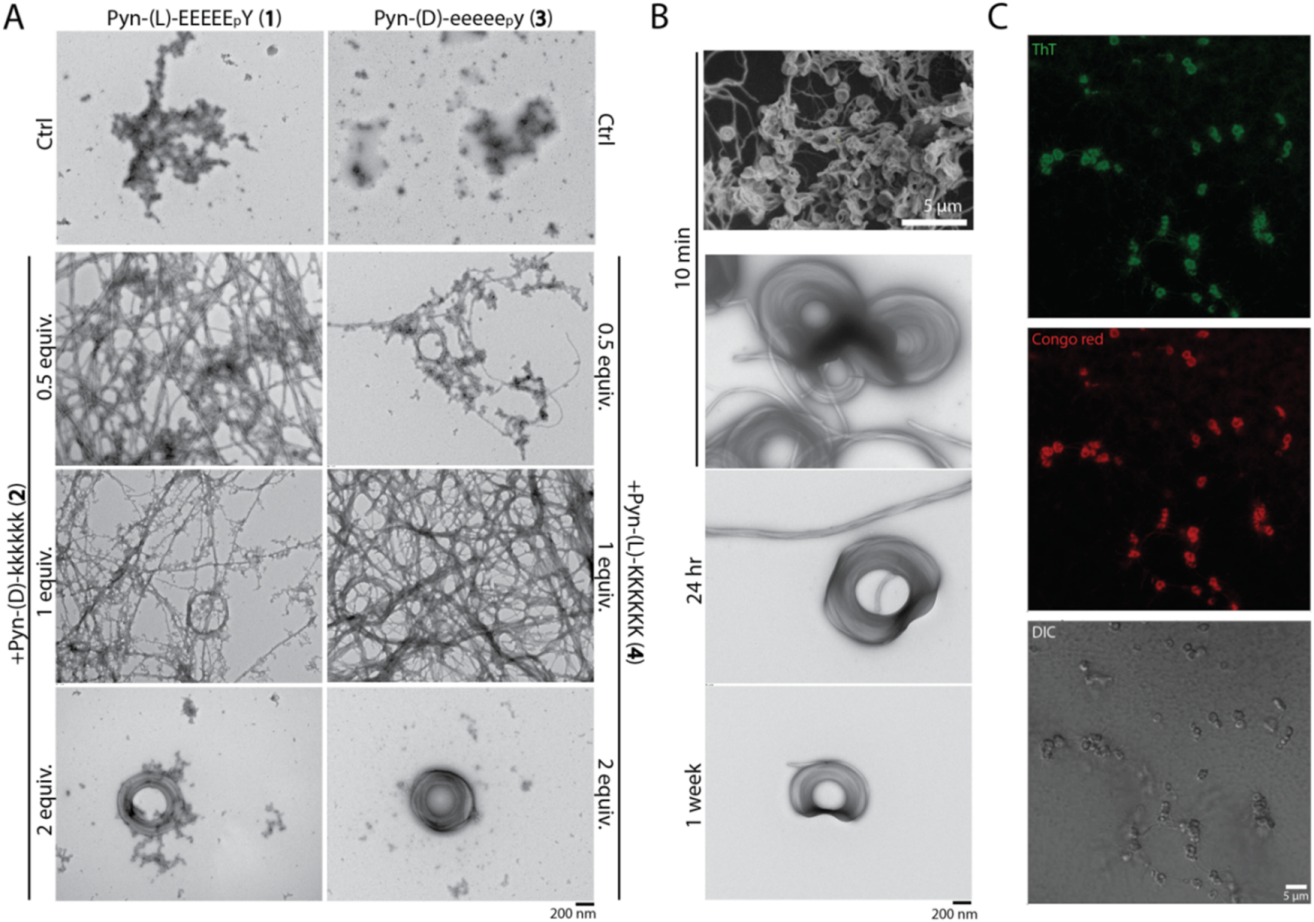
(A) TEM images of 500 μM Pyn-(l)-EEEEE_p_Y (**1**) or Pyn-(d)-eeeee_p_y (**3**) alone and after mixing with 0.5, 1.0, or 2.0 equiv of Pyn-(d)-kkkkkk (**2**) or Pyn-(l)-KKKKKK (**4**) for 24 h in neutral pH water. (B) SEM and TEM images of Pyn-(l)-EEEEE_p_Y (**1**) mixed with 2 equiv of Pyn-(d)-kkkkkk (**2**) after 10 min and corresponding TEM images after incubation for 24 h and 1 week, demonstrating morphological stability over time. (C) CLSM images of Pyn-(l)-EEEEE_p_Y (**1**) with 2 equiv of Pyn-(d)-kkkkkk (**2**) after 24 h incubation, stained with Thioflavin T (green channel) and Congo Red (red channel) to assess amyloid-like features.

These supercoils formed rapidly and in high abundance (Figure 2B). Scanning electron microscopy (SEM) confirmed their widespread presence on the same grid used for TEM imaging, just 10 min after mixing Pyn-(l)-EEEEE_p_Y (**1**) with Pyn-(d)-kkkkkk (**2**). The supercoils displayed interfiber alignment, as revealed by Cryo-EM (Figure S55). The morphology remained stable over time, with few changes observed after 24 h or even 1 week of incubation at room temperature. The supercoils also formed robustly across a broad temperature range. At lower temperatures (4 °C), more twisted fiber bundles appeared to attach to the supercoils, whereas at higher temperatures (37 °C), the structures existed as more isolated and well-defined individual rings (Figure S9). Both concentration and mixing ratio affected the supercoil formation. At 500 μM Pyn-(l)-EEEEE_p_Y (**1**) with two equivalents of Pyn-(d)-kkkkkk (**2**), well-defined and uniform supercoils were consistently formed. However, lowering the overall peptide concentration to 50 or 200 μM resulted in less pronounced structures: the mixture of Pyn-(l)-EEEEE_p_Y (**1**) with 2 equiv of Pyn-(d)-kkkkkk (**2**) resulted in thinner ring-like assemblies; equal molar mixtures primarily produced twisted fiber bundles instead of closed rings (Figures S10–S11). CLSM, combined with Thioflavin T and Congo Red staining, confirmed that β-sheets were dominant in the fibrillar structures that constitute these assemblies (Figure 2C). CD spectra further confirmed the secondary structures in heterotypic mixtures and revealed the mirror-image relationship between enantiomeric peptides and their mixtures (Figure S56A). Individual peptides **1**–**4** displayed random coil features, together with well-defined Cotton effects of opposite sign for d- and l-enantiomers, consistent with their intrinsically disordered nature and validating their enantiomeric relationship. Upon mixing, however, phase separation occurred (Figure S56B), causing much of the assembled material to be excluded from the bulk solution and thereby significantly decreasing the CD signals. Moreover, in heterochiral mixtures the signals largely canceled each other, complicating quantitative interpretation. Nevertheless, the enantiomeric mixtures still produced approximately mirrored CD spectra, confirming the expected chiral symmetry in the assemblies.

### Assemblies by Sequence Variants

#### Heterochiral Assemblies with Glutamic Acid Sequence Variants

To evaluate whether alternative sequence variants could also support supercoil formation, we conducted similar heterochiral mixing experiments using the control IDPs with different lengths and chirality. We mixed Pyn-(l)-EEEE_p_Y (**8**), containing one fewer glutamic acid than Pyn-(l)-EEEEE_p_Y (**1**), with positively charged IDPs containing 3 to 6 lysines. TEM analysis showed that all combinations produced nanofibers or fiber bundles, although the extent of fiber formation depended on lysine content (Figure S12). Specifically, Pyn-(d)-kkkkk (**14**) and Pyn-(d)-kkkkkk (**2**) led to the most prominent fiber formation, whereas Pyn-(d)-kkk (**12**) and Pyn-(d)-kkkk (**13**) primarily yielded nanoaggregates at lower mixing ratios while increased equivalents of the positively charged peptides promoted further fibrillization. CLSM analysis supported these observations (Figure S17). The mixture of Pyn-(l)-EEEE_p_Y (**8**) with Pyn-(d)-kkk (**12**) showed bright fluorescent chunks, consistent with aggregation seen in controls of Pyn-(d)-kkk (**12**) alone. Mixing **8** with Pyn-(d)-kkkk (**13**) produced small aggregates and fluorescent spots, likely corresponding to the nanoaggregates observed by TEM. Mixing Pyn-(l)-EEEE_p_Y (**8**) with Pyn-(d)-kkkkk (**14**) or Pyn-(d)-kkkkkk (**2**) led to extensive fiber formation, though the fibers were thin and often barely distinguishable from the background at 0.5 or 1 equiv. At a 2:1 ratio of Pyn-(d)-kkkkk (**14**) to Pyn-(l)-EEEE_p_Y (**8**), bundled fibers with bright central fluorescence were observed; similarly, mixtures of **8** with Pyn-(d)-kkkkkk (**2**) yielded dense fiber networks. Pyn-(l)-EEE_p_Y (**7**), which contains two fewer glutamic acids, exhibited a similar trend: its heterochiral mixtures with positively charged peptides again showed lysine-dependent assembly. TEM images revealed behavior comparable to Pyn-(l)-EEEE_p_Y (**8**), and CLSM data showed bright aggregates for 3 and 4 lysines, intermediate aggregates or thin sheets for 5 and 6 lysines at 0.5 or 1 equiv, and weakly visible fiber formation at 2 equiv (Figures S13 and S18).

These results of heterochiral assemblies indicate that shorter glutamic acid variants still formed heterochiral assemblies, but the extent and morphology of fiber formation were strongly dependent on lysine content, with higher lysine numbers promoting more robust fibrillization.

#### Homochiral Assemblies with Varied Lysine Content

To further examine homochiral assembly behavior on a broader scale, we used CLSM to observe the mixing of Pyn-(l)-EEEEE_p_Y (**1**) with Pyn-(l)-KKK (**9**), Pyn-(l)-KKKK (**10**), Pyn-(l)-KKKKK (**11**), and Pyn-(l)-KKKKKK (**4**) (Figure S19). As expected, combinations with Pyn-(l)-KKK (**9**) or Pyn-(l)-KKKK (**10**) resulted in particles and disordered aggregates. The mixture with Pyn-(l)-KKKKK (**11**) at a 1:1 ratio, however, produced well-defined and highly fluorescent fiber bundles. Increasing the ratio to 1:2 led to even brighter, thicker bundles. Interestingly, CLSM imaging of the homochiral mixture of Pyn-(l)-EEEEE_p_Y (**1**) and Pyn-(l)-KKKKKK (**4**), which appeared as fiber bundles under TEM (Figure S5), revealed that these bundles were in fact spike-like projections emanating from spherical aggregates.

The trend shows that increasing lysine content in homochiral assemblies drives a transition from disordered aggregates to well-defined fiber bundles, with the highest lysine level producing spike-like projections from spherical aggregates.

#### Heterochiral Assemblies with Varied Lysine Content

To further evaluate the sequence requirements for supercoil formation, we examined heterochiral mixtures of Pyn-(l)-EEEEE_p_Y (**1**) with positively charged IDPs containing fewer lysine residues (Figure S14). Mixing **1** with Pyn-(d)-kkk (**12**) or Pyn-(d)-kkkk (**13**) predominantly formed nanoaggregates and showed no fiber formation; mixtures of **1** with Pyn-(d)-kkkkk (**14**) or Pyn-(d)-kkkkkk (**2**), however, exhibited markedly different behaviors, especially at a broader field of view in CLSM (Figure S20). In the case of Pyn-(d)-kkkkk (**14**), fibers unambiguously formed, and at higher equivalents, these twisted fibers aggregated into large, bright spherical clusters. Notably, some of the fibers curled into ring-like structures at the periphery, though with variable size and morphology. CLSM imaging of the mixture of Pyn-(l)-EEEEE_p_Y (**1**) and Pyn-(d)-kkkkkk (**2**) provided a broader view of the supercoils. These supercoils appeared in high abundance, exhibited relatively uniform diameters, and consistently originated from fiber network scaffolds. The enantiomeric mixture, Pyn-(d)-eeeee_p_y (**3**) with the corresponding positively charged Pyn-(l)-KKKKKK (**4**), produced nearly identical morphologies, confirming that the assembly behavior is consistent and reproducible across mirrored peptide pairs (Figures S15 and S21).

These results suggest that assemblies formed only when the heterochiral mixtures contained peptides with at least five lysines, with longer lysine sequences promoting abundant, uniform supercoil formation, while shorter ones led to nonfibrillar aggregates.

### Pathway Dependency

To further investigate the mechanism underlying supercoil formation, we designed a stepwise mixing experiment to add the required two equivalents of Pyn-(d)-kkkkkk (**2**) in two discrete portions. Initially, we combined Pyn-(l)-EEEEE_p_Y (**1**) with 0.1, 0.2, 0.5, or 1 equiv of Pyn-(d)-kkkkkk (**2**) for incubation of 24 h. Following this preincubation, we added an additional 1.9, 1.8, 1.5, or 1 equiv of Pyn-(d)-kkkkkk (**2**) to reach a final 2:1 ratio of positive to negative peptides. We refer them here as “0.1 + 1.9,” “0.2 + 1.8,” “0.5 + 1.5,” and “1 + 1.”

We used TEM to examine morphological outcomes immediately after the second addition and reassessed them after 24 h. Control experiments showed the expected behavior: incremental addition of Pyn-(d)-kkkkkk (**2**) from 0.1 to 1 equiv progressively enhanced fiber formation, and the 1:1 equimolar mixture began to exhibit early ring-like features. The second addition of Pyn-(d)-kkkkkk (**2**) had, however, markedly different effects depending on the initial mixing ratio (Figures 3A–C and S22). In the “0.1 + 1.9” condition, the additional 1.9 equiv of Pyn-(d)-kkkkkk (**2**) induced immediate and uniform formation of well-defined supercoils (Figure 3A). The “0.2 + 1.8” condition led to a similar outcome, although a slightly higher population of thick fiber bundles coexisted with the supercoils (Figure 3B). For the “0.5 + 1.5” mixture, fiber bundles of varied diameters represented the dominant morphology, featuring significantly fewer supercoils (Figure 3C). Moreover, the ring structures in this condition displayed greater size heterogeneity, with thicknesses ranging from 100 nm to 1 μm and corresponding areas between 0.1 and 6 μm^2^ (Figures 3D and S23). In contrast, the “1 + 1” condition showed few substantial morphological changes following the second addition (Figure S22). The supercoils formed during the initial equimolar mixing remained thin and unchanged in shape, suggesting that the second addition failed to nucleate new ring structures. The opposite enantiomeric pair, Pyn-(d)-eeeee_p_y (**3**) and Pyn-(l)-KKKKKK (**4**), exhibited a similar stepwise trend (Figure S25), confirming that the assembly behavior and pathway dependence are consistent across mirror-image peptide systems.

**Figure 3:**
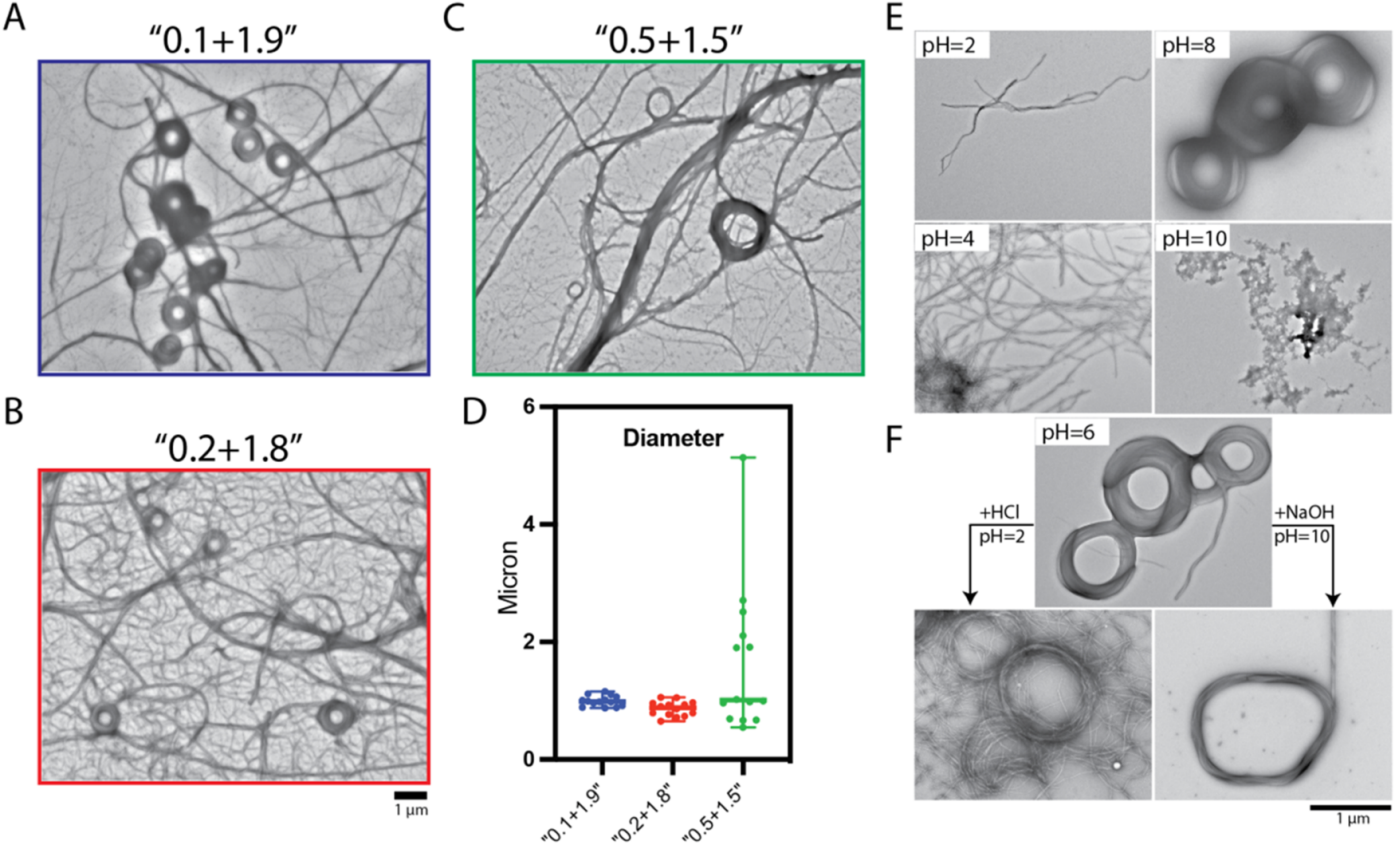
(A–C) TEM images of 1 mM Pyn-(l)-EEEEE_p_Y (**1**) mixed stepwise with Pyn-(d)-kkkkkk (**2**): (A) “0.1 + 1.9”, (B) “0.2 + 1.8”, and (C) “0.5 + 1.5” indicate that 0.1, 0.2, or 0.5 equiv of Pyn-(d)-kkkkkk (**2**) were added and incubated for 24 h prior to the addition of the remaining amount to reach a final 2:1 Pyn-(d)-kkkkkk (**2**):Pyn-(l)-EEEEE_p_Y (**1**) ratio. Samples were incubated for 1 min after the final addition before imaging. (D) Diameter analysis of resulting structures; data are plotted as median with range using GraphPad Prism. (E) TEM images of Pyn-(l)-EEEEE_p_Y (**1**) mixed with Pyn-(d)-kkkkkk (**2**) at various pH values (2, 4, 6, 8, 10), prepared by adjusting the pH of each component individually prior to mixing at a 1:1 volume ratio. (F) TEM images of supercoils formed at pH 6 and then diluted 1:1 with either pH 2 HCl or pH 10 NaOH to assess pH stability under acidic or basic conditions.

CLSM corroborated these findings. Using pyrene fluorescence imaging, we observed similar trends to those seen in TEM, but across a wider field of view (Figures S24 and S26). Supercoils were clearly visible in the “0.1 + 1.9” and “0.2 + 1.8” conditions, consistent with direct 2:1 mixing. In contrast, the “0.5 + 1.5” condition showed a dense fibrous network but no discernible supercoil formation after the second addition within 10 min or after 24 h. For the “1 + 1” condition, supercoils were already present prior to the second addition, and their abundance and morphology remained unchanged afterward, confirming that no additional ring formation occurred.

### pH Dependency

Given the charged nature arising from multiple glutamic acid, phosphotyrosine, and lysine residues in the peptide sequences, we investigated the pH dependence of their self-assembly behavior. Neither component alone formed supercoils across the pH range of 2 to 10 (Figure S27). Pyn-(l)-EEEEE_p_Y (**1**) exhibited fiber formation at acidic pH (2 and 4), while forming disordered nanoparticles at mildly acidic to basic pH (6, 8, and 10), consistent with the deprotonation of glutamic acid at higher pH values. Pyn-(d)-kkkkkk (**2**), composed of six lysines, displayed spherical micelle morphologies from pH 2 to 8 and aggregated nanospheres at pH 10 (Figure S27), implying a potential structural role in guiding supercoil formation upon mixing with peptides of opposite charge and chirality.

Combining two components at pH 2 resulted in entangled helical fibers, which became more aggregated at pH 4. At pH 6, distinctive donut-shaped assemblies emerged and remained stable at pH 8, but dissociated into nanoparticle aggregates at pH 10 (Figure 3E). To assess the stability of these supercoils under extreme conditions, we treated preformed assemblies with either pH 2 HCl or pH 10 NaOH. Acidic treatment for 24 h led to disassembly into thin individual fibers; basic treatment reduced curvature and produced irregular nanoparticles (Figures 3F and S28). The reverse enantiomeric pair, Pyn-(d)-eeeee_p_y (3) and Pyn-(l)-KKKKKK (4), exhibited similar behavior (Figures S29–S30), confirming that supercoils form optimally between pH 6 and 8 but unwind under strongly acidic or basic conditions, likely due to the disruption of electrostatic interactions essential for maintaining their architecture.

Ionic stability tests further support that electrostatic interactions drive supercoil formation (Figure S31). Mixing the peptides directly in Tris-HCl buffer (pH 6.5) results in no supercoils or fibrils. Diluting preformed supercoils with the same Tris-HCl buffer, however, led to disassembly, producing thinner filaments and dispersed nanoparticles. The residual circular shadows (Figure S31) suggest that supercoils initially formed but unwound under these ionic conditions.

### Kinetics

We used CLSM to monitor the dynamic process of ring formation in real time, as illustrated in Figure 4A. After depositing a droplet of Pyn-(l)-EEEEE_p_Y (**1**) solution onto a glass-bottom dish, we carefully added another droplet containing Pyn-(d)-kkkkkk (**2**). Figure 4B shows the progression of ring formation: ring formation typically initiates at regions of preformed fiber networks, as shown in region of interest I (ROI I). Subsequently, a larger, circular droplet nucleates on top of this network. As it evolves, it evaporates while maintaining its circular symmetry, eventually giving rise to a hollow ring structure. The fluorescence intensity profile along the dashed line in ROI I reveals two major peaks separated by a central dip, each well-fitted by bimodal Gaussian curves (Figure 4C). Hence, we define the ring diameter (d) as the distance between these two intensity maxima. The ring diameter stabilizes and reaches a plateau over time, indicating that the structural transition is complete (Figure 4D).

**Figure 4:**
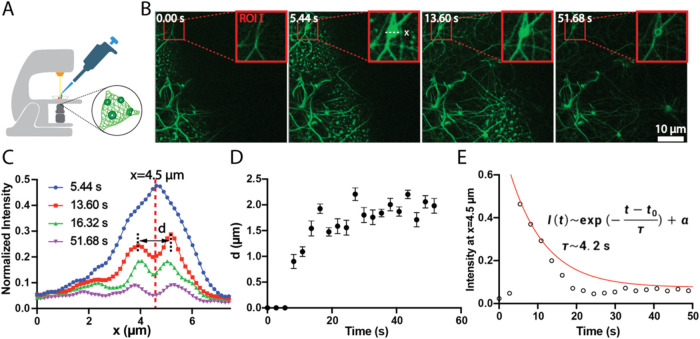
(A) Experimental setup on the microscope for real-time monitoring of ring formation. (B) Time-lapse images showing ring formation during the reaction. Region of interest (ROI) I highlights nucleation on preformed fibers, followed by the emergence of a ring. (C) Time evolution of the normalized fluorescence intensity profiles along the dashed line in panel B. Two distinct peaks at 13.60 s indicate the presence of a ring with a central void. (D) Ring diameter (d), defined as the distance between intensity maxima, rapidly saturates near 2 μm, correlating with the decay of the central intensity. (E) Time evolution of fluorescence intensity at the ring center. The intensity decays rapidly, and the fitted lifetime (τ) is approximately 4.2 s, which is consistent with the time required for diameter saturation.

The changes in fluorescence intensity reflect this morphological evolution from a dense liquid droplet to a stable ring. As shown in Figure 4E, the intensity at the center of the ring decreases rapidly until it becomes comparable with the background noise. This decay is well-fitted by the function *I­(t)* ∼ exp­(−(*t* – *t*_0_)/τ) + α, where the extracted characteristic time τ ≈ 4.2 s matches the time scale for ring diameter saturation in Figure 4C, and α represents the background intensity. This suggests a strong correlation between fluorescence decay and morphological evolution. The kinetic behavior appears to be general. In another region of interest (ROI  II), ring formation also initiates on preformed fiber networks, and the corresponding intensity changes yield a similar τ ≈ 4.8 s, consistent with observations in ROI I (Figure  S32).

To improve temporal resolution during imaging, we added Thioflavin T (ThT) to both peptide solutions before mixing, enabling acquisition of more data points at higher frame rates (Figure S33). The strong fluorescence signal from ThT allowed clear visualization of the dynamic assembly process. Notably, early stage circular structures exhibited liquid-like behaviors, such as coalescence with nearby droplets, and their fluorescence intensity profiles displayed dome-shaped, spherical-cap-like distributions distinct from simple Gaussian curves (Figure S33B). This supports the presence of a condensed, droplet-like intermediate.^51^ The observed kinetics in ThT-containing samples reflect not only the time required for supercoil formation but also include contributions from ThT binding to β-sheet structures and subsequent emission, which may not represent the intrinsic assembly kinetics. Nonetheless, the overall kinetic trends remain consistent, offering valuable insights into the properties of the circular intermediates preceding ring formation.

### Proteolytic Stability

To assess the proteolytic stability of the assemblies, we exposed the peptide mixtures to proteinase K, a broad-spectrum protease with high substrate versatility and strong peptide-cleaving activity^52,53^ and compared proteolysis kinetics with those of the corresponding monomeric peptides at identical concentrations. At designated time points, we quenched the reactions and analyzed them by LC/MS (Figures S34, S52–S53, and Table S1). The monomeric Pyn-(l)-EEEEE_p_Y (**1**) underwent almost complete proteolysis, catalyzed by 1 mg/mL proteinase K, within 30 min (Figure 5A); the heterochiral mixture with Pyn-(d)-kkkkkk (**2**), known to form the supercoil assemblies, remained largely intact for up to 24 h (Figure 5B). TEM analysis confirmed the morphological stability of the supercoils treated with proteinase K (Figures 5E and S37).

**Figure 5:**
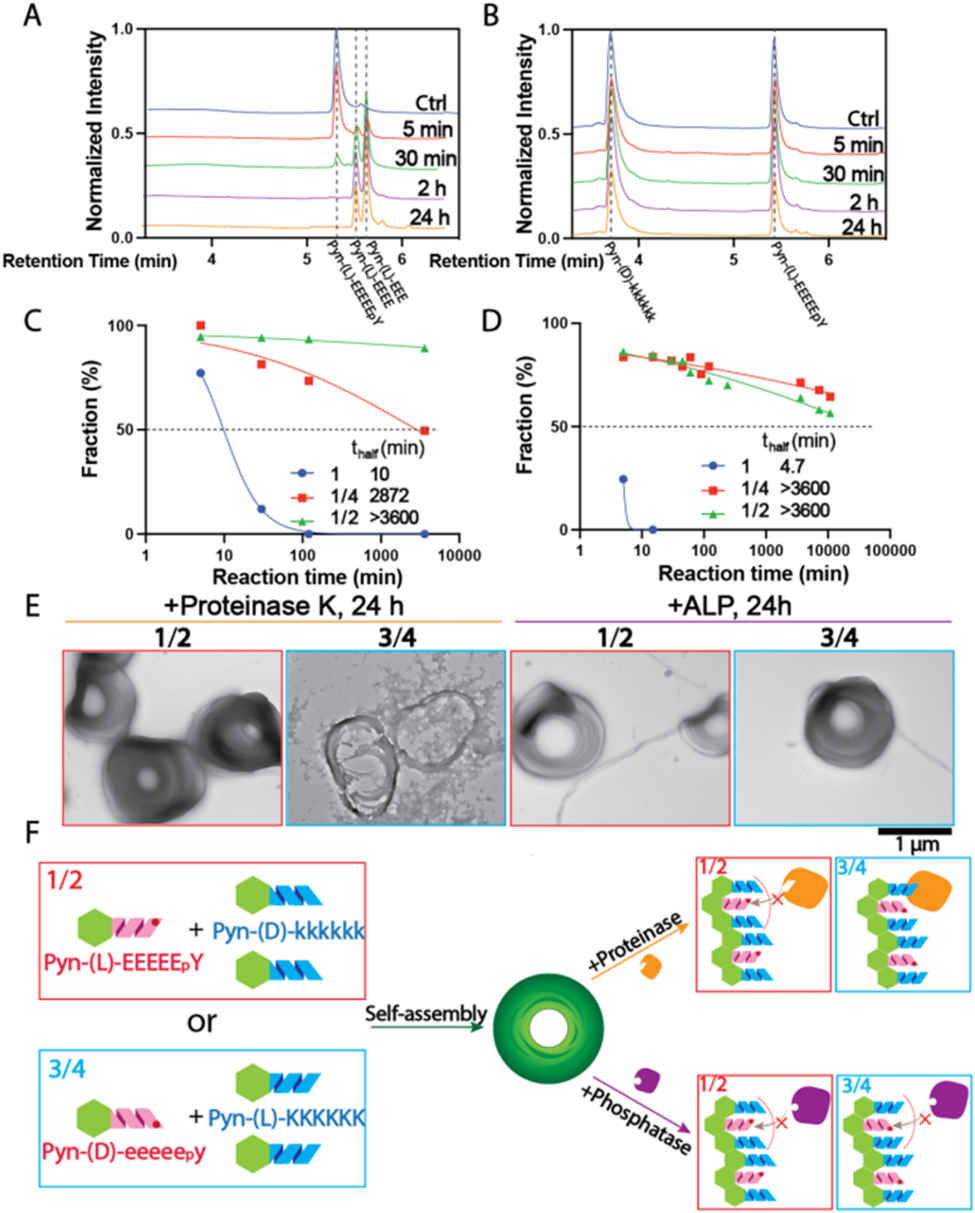
(A) HPLC traces of 1 mM Pyn-(l)-EEEEE_p_Y (**1**) and (B) 500 μM Pyn-(l)-EEEEE_p_Y (**1**) mixed with 2 equiv of Pyn-(d)-kkkkkk (**2**), treated with 1 mg/mL proteinase K at 37 °C and neutral pH for 5 min, 30 min, 2 h, and 24 h. (C) Kinetic analysis of proteinase K digestion based on HPLC peak areas for Pyn-(l)-EEEEE_p_Y alone (**1**, blue dots), Pyn-(l)-EEEEE_p_Y mixed with 2 equiv of Pyn-(l)-KKKKKK (**1**/**4**, red squares), and Pyn-(l)-EEEEE_p_Y mixed with 2 equiv of Pyn-(d)-kkkkkk (**1**/**2**, green triangles). (D) Kinetics of ALP-mediated dephosphorylation under similar conditions using 1 U/mL ALP. (E) TEM images of **1**/**2** and **3**/**4** samples after 24 h treatment with proteinase K or ALP. (F) Proposed structural model illustrating the protective role of supercoils in enzymatic reactions.

Quantitative LC analysis revealed that cleavage of the Pyn-(l)-EEEEE_p_Y/Pyn-(d)-kkkkkk mixture (**1**/**2**) proceeded over 360 times more slowly than for Pyn-(l)-EEEEE_p_Y (**1**) alone, which exhibited a half-life of approximately 10 min (Figure 5C). Similarly, the homochiral Pyn-(l)-EEEEE_p_Y/Pyn-(l)-KKKKKK assembly (**1**/**4**) also showed enhanced proteolytic resistance, with a 287-fold increase in half-life compared to the l-peptide alone. Unlike the supercoil morphology observed in heterochiral mixtures, the homochiral assembly formed more linear fibers and bundles, which disassembled into irregular nanoparticles and aggregates upon protease treatment. Interestingly, Pyn-(l)-KKKKKK (**4**) alone was largely resistant to proteolysis, but incorporation of a negatively charged partner altered this stability. Mixing with Pyn-(l)-EEEEE_p_Y (**1**) accelerated the degradation of Pyn-(l)-KKKKKK (**4**) by nearly 5-fold; pairing with Pyn-(d)-eeeee_p_y (**3**), however, led to more than a 100-fold increase in cleavage rate (Figure S35B). This observation indicates that enantiomeric pairing has a more pronounced effect on proteolytic susceptibility than charge complementarity.

The above observations suggest a structural model in which the d- or l- Pyn-KKKKKK (**2** or **4**) segments are predominantly displayed on the surface of the supercoils, effectively shielding the l- or d-Pyn-EEEEE_p_Y (**1** or **3**) peptides within. In the **1**/**2** assembly, the surface-exposed Pyn-(d)-kkkkkk (**2**) is protease-resistant, protecting the cleavable Pyn-(l)-EEEEE_p_Y (**1**) inside and thereby resulting in exceptional stability. Conversely, in the **3**/**4** assembly, the surface-exposed Pyn-(l)-KKKKKK (**4**) is protease-susceptible, enabling rapid enzymatic access and facilitating processive degradation. This surface presentation of enantiomers explains the stark differences in proteolytic stability across the assemblies (Figure 5F).

To further validate the structural hypothesis, we performed enzymatic assays using ALP, which removes phosphate groups from both d- and l-tyrosines and has been widely used to explore enzyme-instructed self-assembly (EISA).^54,55^ This setup isolates Pyn-EEEEE_p_Y as the reactive component, allowing us to assess the impact of coassembly with Pyn-KKKKKK on dephosphorylation kinetics. Using the same protocol as the protease experiments, we measured the rate of dephosphorylation for Pyn-EEEEE_p_Y monomers and their homo- and heterochiral mixtures with two equivalents of Pyn-KKKKKK (Figure S54 and Table S2). As shown in Figures 5D and S36B, both Pyn-(l)-EEEEE_p_Y (**1**) and Pyn-(d)-eeeee_p_y (**3**) underwent complete dephosphorylation within 30 min, with **3** (*t*_half_ = 9.6 min) reacting about twice as slowly as **1** (*t*_half_ = 4.7 min). However, coassembly with Pyn-KKKKKK significantly slowed the dephosphorylation. In **1**-containing assemblies (**1/2** and **1/4**), less than 50% dephosphorylation occurred even after 3 days, indicating significant protection. **3**-containing assemblies showed similar trends, with **3/2** reaching half-dephosphorylation in approximately 1 day. These results support the hypothesis that Pyn-EEEEE_p_Y is embedded within the nanostructures and shielded from enzymatic access by surface-exposed Pyn-KKKKKK, consistent with the proposed structural arrangement of the assemblies (Figure 5F).

## Conclusion

In summary, this study reports the spontaneous formation of highly stable peptide supercoils through the heterochiral coassembly of oppositely charged IDPs. These supercoils form rapidly in aqueous solution under physiological pH, exhibiting structural stability over extended periods and remarkable resistance to enzymatic degradation. Structural analogs and kinetic analyses reveal that electrostatic interactions and molecular chirality critically influence the self-assembly pathway and final morphology. Fluorescence microscopy uncovers a dynamic two-step formation mechanism involving transient liquid-like droplets that transition into solid fibrillar rings. Moreover, structural organization, particularly the surface presentation of the protease-resistant d-peptide, plays a key role in shielding l-peptides from enzymatic proteolysis. Enzymatic assays confirm that supercoil formation creates a microenvironment unfavorable for enzymatic access, supporting the steric and morphological protection. Although the detailed atomistic interactions between these heterochiral peptides remain to be elucidated due to the current technological limitation, the tightly packed nature of the supercoils prevents cryo-EM averaging of individual fibers, in contrast to the homochiral assemblies in our previous work.^5^ Nevertheless, the “sandwich” architectures proposed in Figures 1 and 5 are consistent with the earlier cryo-EM structure of oppositely charged IDP homochiral assemblies.^5^ Although pyrene facilitates self-assembly of these IDPs by providing hydrophobic interaction, it should be replaceable by other hydrophobic motifs,^56–58^ as shown in the recent IDP assemblies containing Nap-FF,^6^ which supports the generality of such IDP assemblies. Toroidal architectures are rare in peptide self-assembly^59–61^ and may arise from multiple contributing factors. We speculate that chirality-guided interfibrillar interactions promote curvatures for generating supercoils. Electrostatic interactions between lysine and glutamic acid residues, along with hydrophobic aromatic interactions, play essential roles in driving ordered morphologies, as demonstrated in the cryo-EM structure of KFE8.^62^ Moreover, heterochiral mixtures often favor unusual morphologies, potentially leading to twisted or helical intermediates that may contribute to toroidal formation, as suggested by prior (FKFE)­n studies.^63–65^ Although force field–based molecular modeling appears useful for elucidating the molecular interactions in the supercoils, their large size and dynamic heterogeneity preclude accurate computational modeling with current methods. In our work, the heterotypic, heterochiral assemblies not only give rise to toroidal supercoils but also display pronounced resistance to proteinase K digestion (Figure 5), underscoring their stability against enzymatic degradation. This protease resistance is particularly advantageous for potential applications in biomaterials and nanotechnology, where long-term structural integrity under biological conditions is essential. Furthermore, the reproducibility of the assembly behavior across enantiomeric peptide pairs highlights the robustness and designability of these supramolecular systems. These findings highlight a valuable design principle for constructing protease-stable nanostructures and provide mechanistic insight into peptide self-assembly driven by electrostatic interactions guided by chirality, and enhanced by hydrophobic interactions (including π–π interactions^66,67^ ). The demonstrated stability and responsiveness of these supercoils suggest broad potential in biomaterials, drug delivery, and nanotechnology applications.

## Supplementary Material



## Notes

Detailed experimental procedures and materials; LC/MS results of individual compounds and enzymatic degradation experiments; fluorescence emission spectra and CAC fitting data; TEM and CLSM images of additional peptide mixtures; additional kinetic analyses (PDF)
