# Supplementary material for: Heterochiral and Heterotypic Self-Assembly of Intrinsically Disordered Peptides Confers Peptide Supercoils with Exceptional Proteolytic Stability
Source: J Am Chem Soc. 2025 Oct 21;147(44):40727–38. doi: 10.1021/jacs.5c13598 (PMC12593409; doi:10.1021/jacs.5c13598)
Supplement: Supplementary file 1 [file ja5c13598_si_001.pdf]

Supporting information for

Heterochiral and Heterotypic Self-Assembly of Intrinsically Disordered Peptides Confers Peptide Supercoils with Exceptional Proteolytic Stability

Yuchen Qiao[1], Myeonggon Park[2], Matthew Chu[1], Grace Wu[1], Ruipeng Guo[3], Chen Liu[4], Hongjian He[1], Tongyu Li[3], Lei Tian[3], Xixiang Zhang[4], W. Benjamin Rogers[2], Bing Xu[1]*

[1]Department of Chemistry, Brandeis University, 415 South St., Waltham, MA 02453, USA

[2]Martin A. Fisher School of Physics, Brandeis University, Waltham, MA 02453, USA

[3]Department of Electrical and Computer Engineering, Boston University, Boston, MA, 02215, USA

[4]Physical Science and Engineering Division, King Abdullah University of Science and Technology, Thuwal, 23955-6900, Saudi Arabia

Table of Contents

Supplemental Experimental Procedures 3

Supporting Data 10

Supplemental Experimental Procedures

Materials

2-Cl-trityl chloride resin (1.02 mmol/g) and Fmoc protected amino acid building blocks were purchased from GL Biochem (Shanghai, China).O-(Benzotriazol-1-yl)-N,N,N′,N′-tetramethyluronium hexafluorophosphate (HBTU) was purchased from Chem impex. N, N-diisopropylethylamine (DIPEA) were purchased from TCI America. 1-pyreneacetic acid was purchased from Sigma-Aldrich. Proteinase K was purchased from Sigma-Aldrich (Proteinase K from Tritirachium album, Product Number P2308), Alkaline phosphatase (ALP) was purchased from Biomatik (Cat. No. A1130, 30000 U/mL, in 50% Glycerol). Dimethylformamide (DMF), methylene chloride (DCM), trifluoroacetic acid (TFA), methanol (MeOH), triethylamine, and other reagents and solvents were purchased from Fisher Chemical. All reagents and solvents were used without further purification.

Instruments

All crude compounds were purified using a reverse phase HPLC (Agilent 1100 Series) with HPLC grade water (0.1% TFA) and HPLC grade acetonitrile (0.1% TFA) as eluents. LC-MS spectra were obtained on a Bruker timsTOF Pro Mass Spectrometer equipped with an Elute UHPLC chromatograph. TEM was conducted on Morgagni 268 transmission electron microscope. Cryo-EM samples were plunge-frozen using an FEI Vitrobot Mark IV and imaged on a Tundra Cryo-TEM equipped with an x-FEG source and Falcon-C direct electron detector. CLSM images were acquired using “Dagahra” Nikon AX-R Resonant scanner. Fluorescence emission spectra were obtained using Shimadzu RF-5301PC spectrometer.

Peptide synthesis

Synthesis of Fmoc-L-Tyr(PO_3H_2)-OH: The mixture of P_2O_5 (35 mmol, 10 g), H_3PO_4 (133 mmol, 13 g) and H-L-Tyr-OH (18 mmol, 3.22 g) was stirred for 24 h at 80 ºC in N_2 atmosphere. After adding 30 mL H_2O and stirred for 30 min at 80ºC, the reaction mixture was cool to room temperature. The reaction mixture was added to 1-butanol (650 mL) dropwise and recrystallized at 4 ºC overnight, filtration provided H-L-Tyr(PO_3H_2)-OH as white power. To the solution of H-L-Tyr(PO_3H_2)-OH (2 mmol, 522 mg) in H_2O (5 mL), the solution of Fmoc-OSu (2.4 mmol, 808 mg) in MeCN (5 mL) was added. After adjusting pH to ~9 by triethylamine (TEA), the solution was stirred at room temperature for 30 min. After removal of MeCN by evaporation, 50 mL H_2O was added and the pH of the solution was adjusted to ~2 by 12 M HCl. After extraction by ethyl acetate (50 mL × 3), the organic part was washed by 1 M HCl (100 mL × 2), H_2O (100 mL × 2). After being dried by Na_2SO_4, filtered and concentrated by evaporation, Fmoc-L-Tyr(PO_3H_2)-OH was provided as white powder. The synthesis of Fmoc-D-Tyr(PO_3H_2)-OH follows the same procedure, except H-L-Tyr-OH is replaced with H-D-Tyr-OH.

*Synthesis of **1-14**:* Compound **1-14** were synthesized via solid-phase peptide synthesis (SPPS). 2-Cl-trityl chloride resin was dipped in methylene chloride (DCM) for 5 min, followed by loading the amino acid building blocks using N, N-diisopropylethylamine (DIPEA) in DCM overnight. A capping solution (DCM:MeOH:DIPEA = 17:2:1) was added for 30 min, and 20% piperidine in dimethylformamide (DMF) was added for another 30 min for deprotection. For subsequent couplings, amino acids, HBTU, HOBt, and DIPEA were loaded for 2 h, with washing steps with DMF after each coupling. Peptides were

cleaved using trifluoroacetic acid (TFA) and left to react for 1 h. After concentrating the reaction mixtures, ethyl ether was added for peptide precipitation. The crude peptides were then purified by HPLC.

Fluorescent spectra measurement

The fluorescence emission spectra of 400 μL solutions for each sample were recorded from 360 nm to 600 nm. All fluorescence emission spectra were obtained using Shimadzu RF-5301PC spectrometer with an excitation wavelength of 355 nm.

Critical aggregation concentration (CAC) measurement

The critical aggregation concentrations (CAC) were assessed utilizing the fluorescent spectra of pyrene motif on the peptides. Various concentrations of a peptide were prepared in distilled water. By plotting the intensity ratios at 374 nm and 384 nm (I_1/I_3) for each concentration, the concentration at the intersection point of two fitted lines was identified as the CAC.

Transmission electron microscopy (TEM) sample preparation

After placing 5 μL samples on 400 mesh copper grids coated with continuous thick carbon film (~35 nm) which was glowed discharged, we washed the grid with ddH_2O and UA (uranyl acetate). The sample loaded grid was stained with the UA for 20 seconds. The residual UA was removed by filter paper and then dried in air. TEM images were obtained with FEI Morgagni 268 80 kV with a 1 k × 1 k AMT CCD camera.

Confocal laser scanning microscopy (CLSM) Imaging

CLSM images were acquired using a Nikon AX-R (“Dagahra”) system. For pyrene fluorescence, images were captured with a 60× oil-immersion objective at 1024 × 1024 resolution using a Galvano unidirectional scanner (dwell time: 2.0 μsec). Excitation was performed with a 405 nm laser at 20% power and gain set to 20, with emission collected from 419–599 nm. All images were adjusted for brightness and contrast in ImageJ, with minimum and maximum values set to 50 and 250, respectively.

For Thioflavin T (ThT)-labeled samples, images were acquired with the same 60× oil objective at 2048 × 2048 resolution using a resonant bidirectional scanner. Excitation was performed with a 488 nm laser at 1% power and gain of 20, with emission collected from 499–625 nm.

Sample preparation

Stock solution: Peptides were dissolved in double-distilled water at 10 mM as stock solution, with the pH adjusted to 7.0 using 1 M hydrochloric acid or sodium hydroxide. The stock solution was diluted to the desired concentration with double-distilled water for reactions.

Mixtures: Mixtures were prepared by combining equal volumes of two components at the desired concentrations (e.g., “500 μM Pyn-(L)-EEEEE$_p$Y + 2 equiv. Pyn-(D)-kkkkkk” was made by mixing equal volumes of 1 mM Pyn-(L)-EEEEE$_p$Y and 2 mM Pyn-(D)-kkkkkk).

For stepwise mixing, the “0.1+1.9” condition refers to a mixture of 10 μL of 2 mM Pyn-(L)-EEEEE$_p$Y and 10 μL of 200 μM Pyn-(D)-kkkkkk incubated for 24 hours, followed by the addition of 20 μL of 1.9 mM Pyn-(D)-kkkkkk in water. Other stepwise combinations

followed the same volumes and incubation conditions, with variations only in the initial concentration of Pyn-(D)-kkkkkk.

For reactions under various pH conditions, stock solutions of each component were diluted with water adjusted to the desired pH using 1 M hydrochloric acid or sodium hydroxide. The pH was rechecked after mixing to ensure accuracy.

Enzymatic Reactions

Proteinase K Treatment: To 200 μL solutions containing peptides **1**, **2**, **3**, **4**, and their mixtures (**1/2**, **1/4**, **3/2**, **3/4**) in double-distilled water, 2 μL of a 100 mg/mL proteinase K stock solution was added to achieve a final concentration of 1 mg/mL. The reactions were incubated at 37 °C. For controls, 40 μL aliquots were withdrawn from each solution prior to enzyme addition. At designated time points (5 min, 30 min, 2 h, and 24 h), 40 μL aliquots were rapidly frozen in liquid nitrogen to stop the reaction. Samples were then lyophilized at −80 °C, reconstituted in 0.1% TFA in H2O/acetonitrile (1:1 v/v), filtered, and analyzed by LC/MS.

LC/MS analysis was performed with solvent A (water + 0.1% formic acid) and solvent B (acetonitrile + 0.1% formic acid) with a gradient program using the following separation method: 0 min 95%A, 5%B; 1 min 95%A, 5%B; 8 min 1%A, 99%B; 13min 1%A, 99%B; 13.1 min 95%A, 5%B; 14 min 95%A, 5%B. The mass spectrometer was operated in positive ion mode, scanning a range of 50–3000 m/z.

Alkaline Phosphatase (ALP) Treatment: the same sample preparation protocol was used, with the enzyme concentration adjusted to 1 U/mL ALP instead of Proteinase K. Reaction time points included 5, 15, 30, 45, 60, 90, 120, and 240 minutes, 24, 48, and 72 hours. LC/MS separation method: 0 min 95%A, 5%B; 1 min 95%A, 5%B; 3 min 50%A, 50%B; 7 min 20%A, 80%B; 7.1 min 1%A, 99%B; 10min 1%A, 99%B; 10.1 min 95%A, 5%B; 11 min 95%A, 5%B. The mass spectrometer was operated in positive ion mode, scanning a range of 50–3000 m/z.

Data analysis

TEM analysis: TEM images were analyzed using ImageJ. Measurements included both the inner and outer rings of the donuts for area and shape fitting (ellipse). Ring area was calculated as the difference between the outer and inner ellipse areas. Ring diameter was determined as the average of the major and minor axes of the fitted outer ellipse. Ring thickness was calculated as half of the difference between the diameters of the outer and inner ellipses. Circularity was determined as the average circularity of the fitted inner and outer ellipses. Each parameter was measured from 14 independent samples and reported as median with range using GraphPad Prism.

LC/MS Analysis: Representative MS spectra for key peaks are shown in Figure S52-53. Peak areas from enzymatic reaction samples were manually integrated and summarized in Table S1-2.

In Proteinase K analyses, for Pyn-(L)-EEEEE$_p$Y (**1**), peaks at retention times 3.9 min (pk3.9), 4.4 min (pk4.4), 4.6 min (pk4.6), and 5.1 min (pk5.1) were summed and normalized to 100%. For Pyn-(L)-KKKKKK (**4**), peaks at 5.6 min (pk5.6), 5.8 min (pk5.8), 5.9 min (pk5.9), and 6.4 min (pk6.4) were summed and normalized to 100%.

In ALP analyses, the peaks at 4.5 min (pk4.5, phosphorylated precursors **1** or **3**) and 4.7 min (pk4.7, dephosphorylated products **5** or **6**) were summed and normalized to 100%.

Kinetics analysis: The CLSM image processing and kinetic analysis of ring formation were performed using custom MATLAB scripts. The intensity normalization in Figure. 4, S32, and S33 were done independently. Fitting errors were quantified using mean squared error, and the error bars in the figures represent the range corresponding to 90% confidence.

Supporting Data

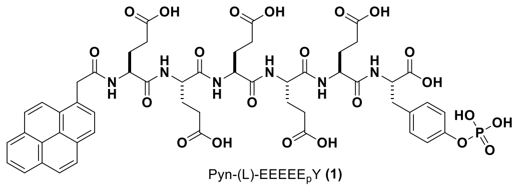

Pyn-(L)-EEEEE$_p$Y **(1)**

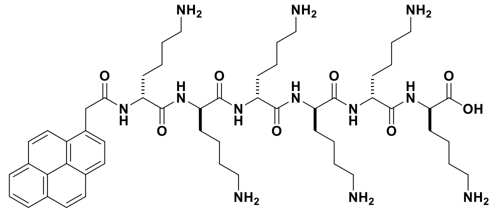

Pyn-(D)-kkkkkk **(2)**

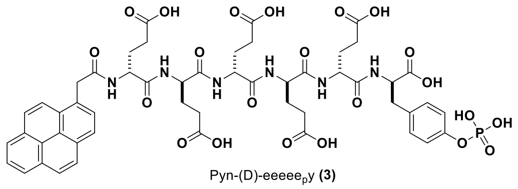

Pyn-(D)-eeeee$_p$y **(3)**

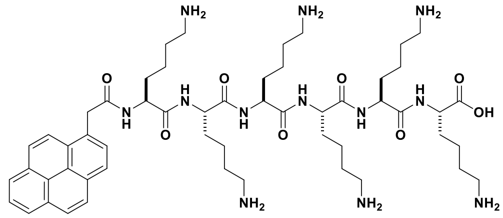

Pyn-(L)-KKKKKK **(4)**

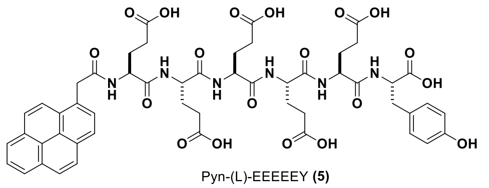

Pyn-(L)-EEEEEY **(5)**

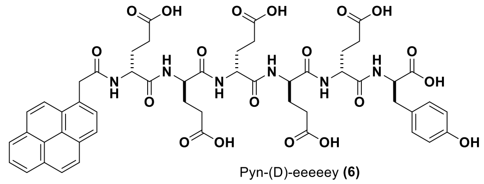

Pyn-(D)-eeeeey **(6)**

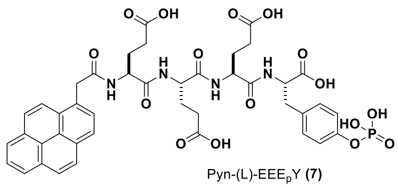

Pyn-(L)-EEE$_p$Y **(7)**

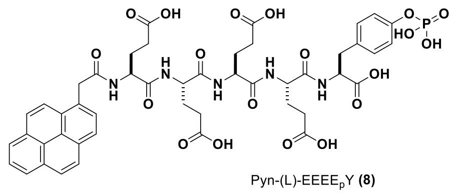

Pyn-(L)-EEEE$_p$Y **(8)**

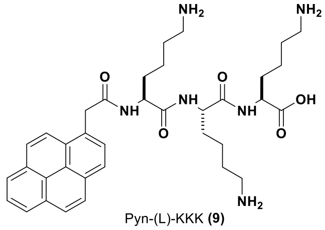

Pyn-(L)-KKK **(9)**

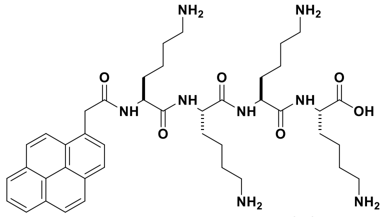

Pyn-(L)-KKKK **(10)**

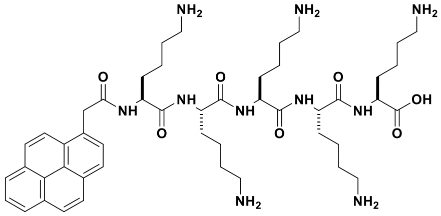

Pyn-(L)-KKKKK **(11)**

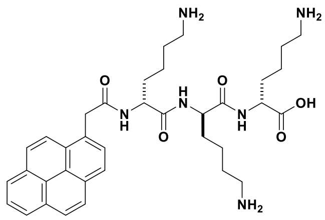

Pyn-(D)-kkk **(12)**

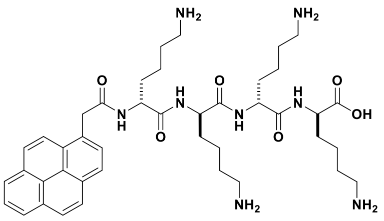

Pyn-(D)-kkkk **(13)**

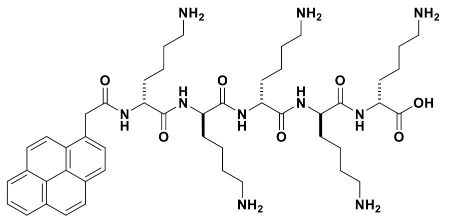

Pyn-(D)-kkkkk **(14)**

Figure S1. Chemical structures of peptides **1-14**.

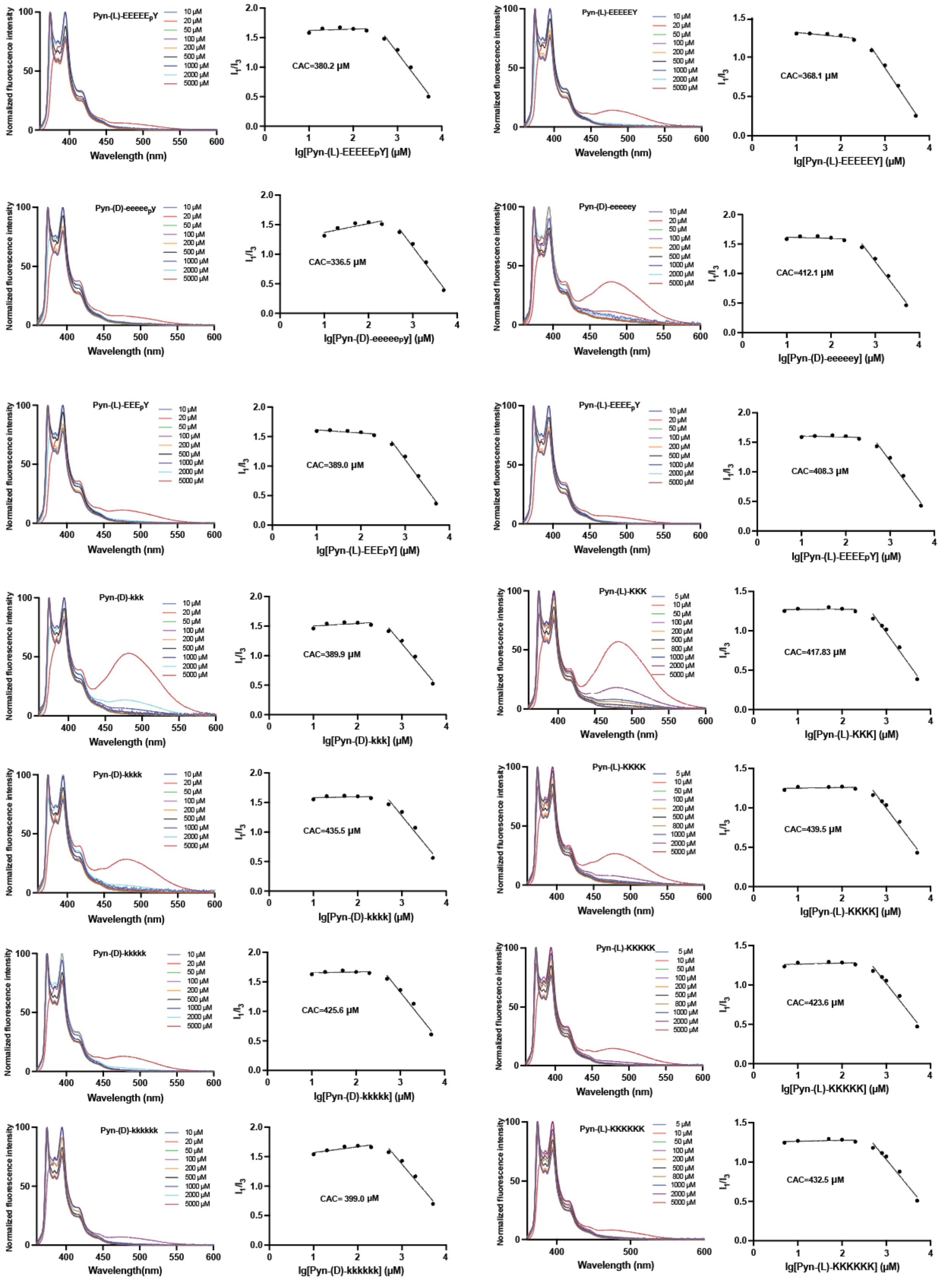
Pyn-(L)-EEEEEpY
Normalized fluorescence intensity
Wavelength (nm)
CAC=380.2 μM
I1/I3
lg[Pyn-(L)-EEEEEpY] (μM)
Pyn-(L)-EEEEEY
CAC=368.1 μM
lg[Pyn-(L)-EEEEEY] (μM)
Pyn-(D)-eeeeepY
CAC=336.5 μM
lg[Pyn-(D)-eeeeepy] (μM)
Pyn-(D)-eeeeey
CAC=412.1 μM
lg[Pyn-(D)-eeeeey] (μM)
Pyn-(L)-EEEpY
CAC=389.0 μM
lg[Pyn-(L)-EEEpY] (μM)
Pyn-(L)-EEEEpY
CAC=408.3 μM
lg[Pyn-(L)-EEEEpY] (μM)
Pyn-(D)-kkk
CAC=389.9 μM
lg[Pyn-(D)-kkk] (μM)
Pyn-(L)-KKK
CAC=417.83 μM
lg[Pyn-(L)-KKK] (μM)
Pyn-(D)-kkkk
CAC=435.5 μM
lg[Pyn-(D)-kkkk] (μM)
Pyn-(L)-KKKK
CAC=439.5 μM
lg[Pyn-(L)-KKKK] (μM)
Pyn-(D)-kkkkk
CAC=425.6 μM
lg[Pyn-(D)-kkkkk] (μM)
Pyn-(L)-KKKKK
CAC=423.6 μM
lg[Pyn-(L)-KKKKK] (μM)
Pyn-(D)-kkkkkk
Wavelength(nm)
CAC= 399.0 μM
lg[Pyn-(D)-kkkkkk] (μM)
Pyn-(L)-KKKKKK
CAC=432.5 μM
lg[Pyn-(L)-KKKKKK] (μM)

Figure S2. The critical aggregation concentrations (CACs) of all IDPs in water at pH=7. I_1/I_3 at y-axis indicates the fluorescence intensity ratios of each peptide at 374 nm (I_1) and 384 nm (I_3).

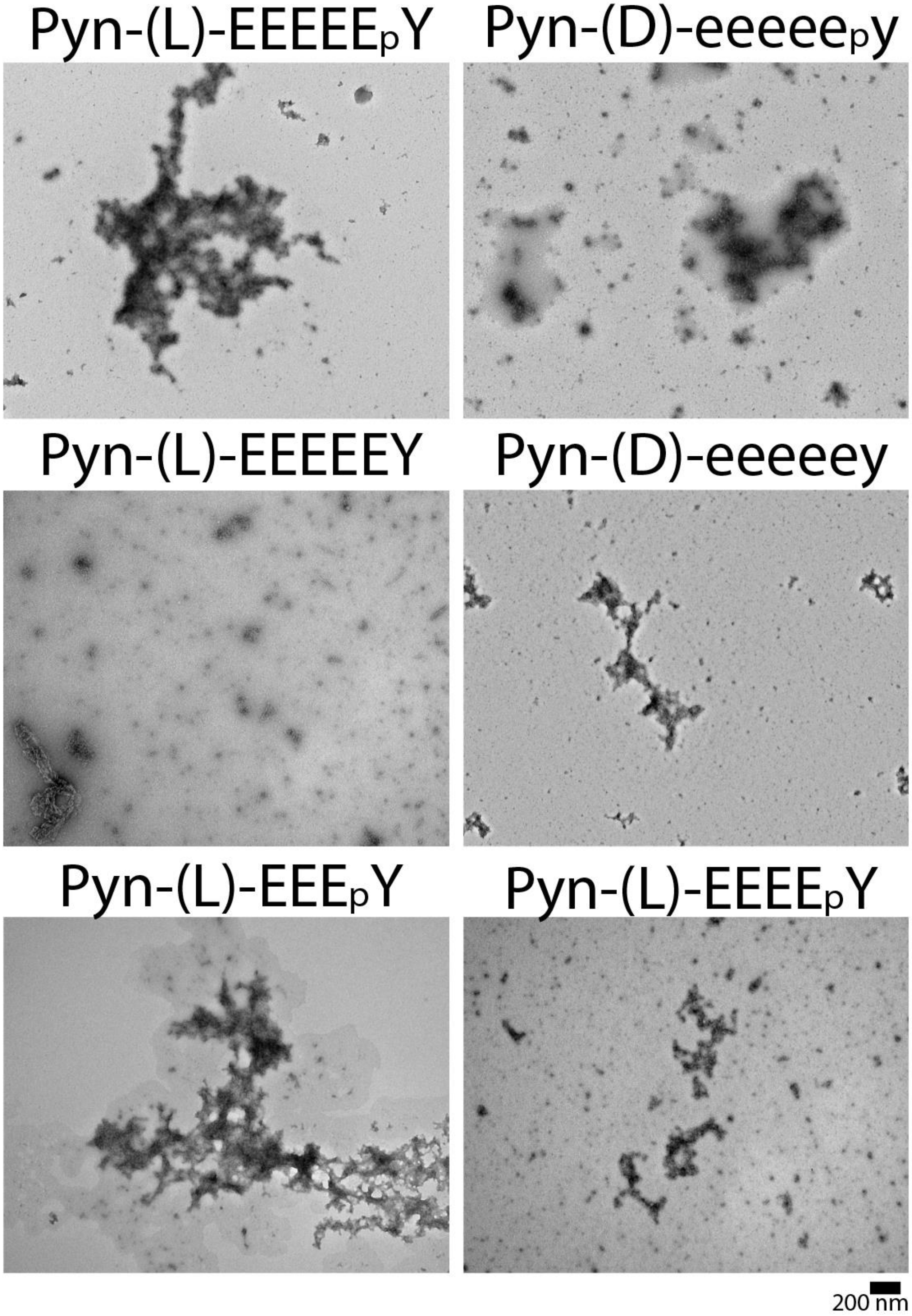

Figure S3. TEM images of all negatively charged IDPs at 500 μM in water at pH 7.

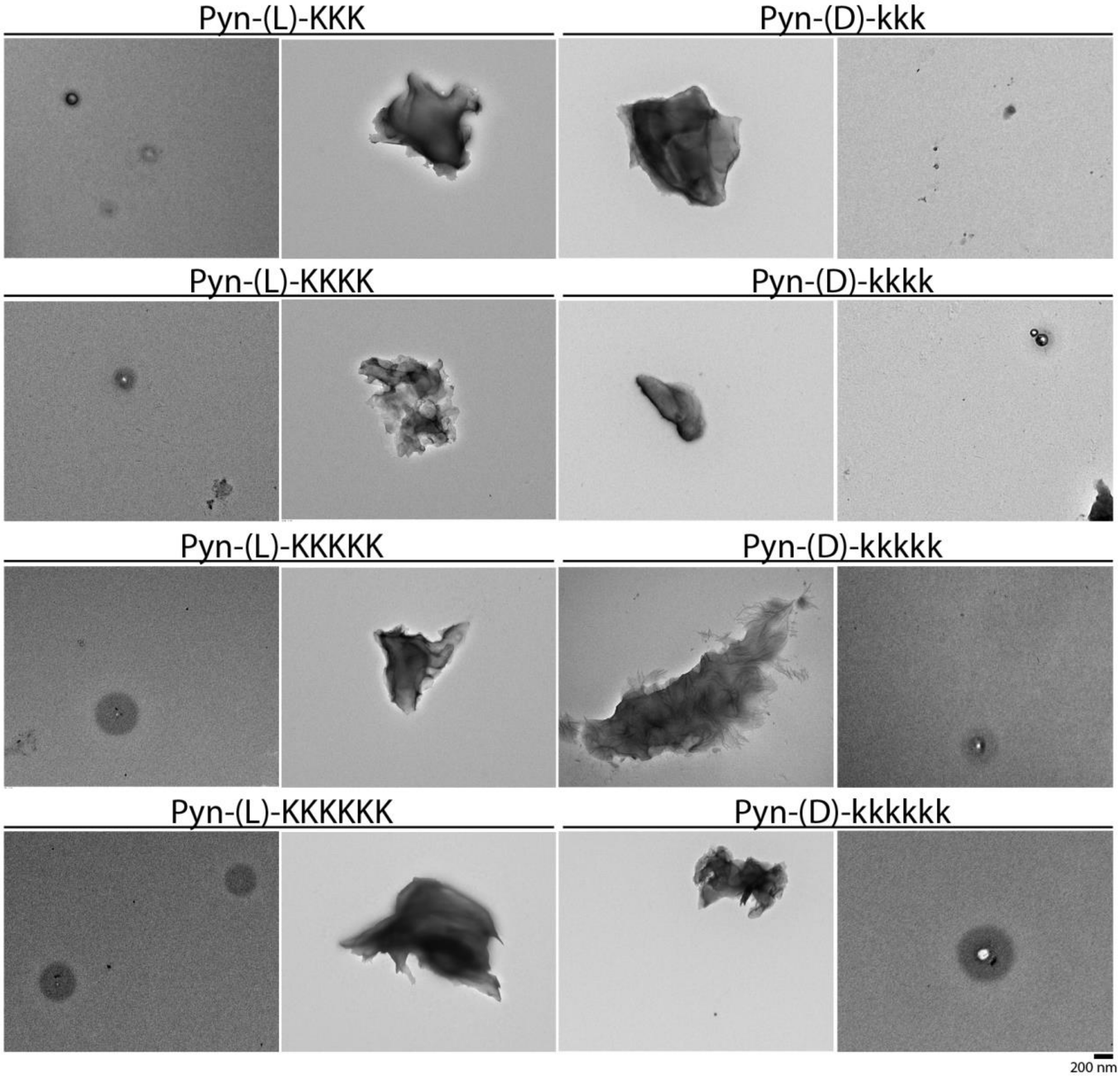

Figure S4. TEM images of all positively charged IDPs at 500 μM in water at pH 7.

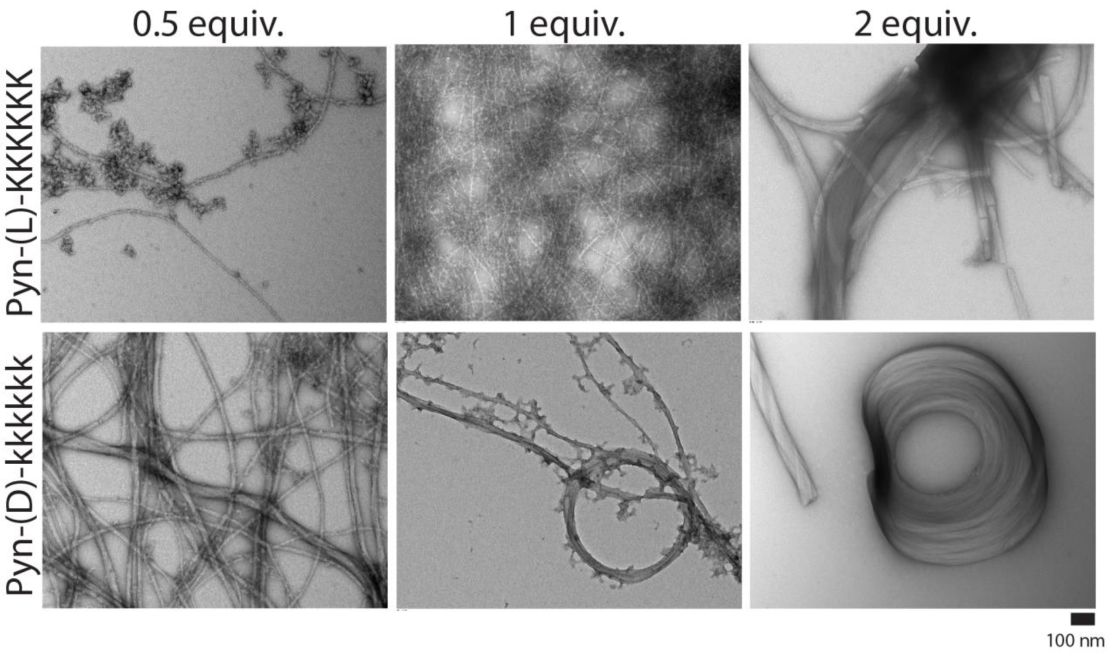

Figure S5. TEM images of 500 μM Pyn-(L)-EEEEE$_p$Y (**1**) mixed with 0.5, 1, and 2 equivalence of Pyn-(L)-KKKKKK (**4**) and Pyn-(D) kkkkkk (**2**) in water.

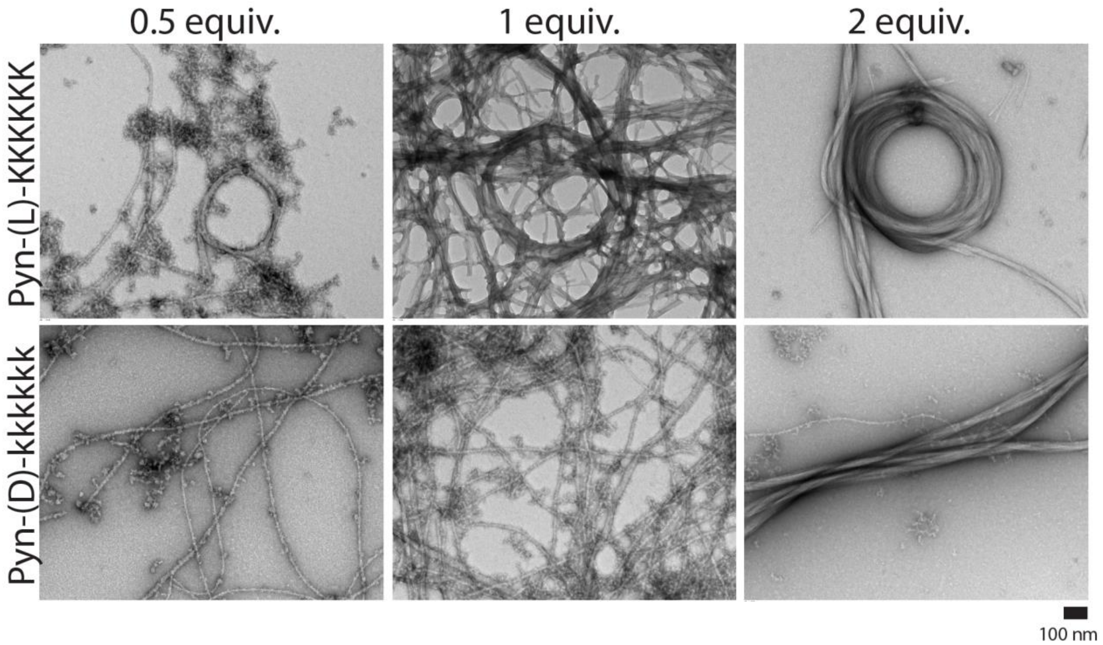

Figure S6. TEM images of 500 μM Pyn-(D)-eeeee$_p$y (**3**) mixed with 0.5, 1, and 2 equivalence of Pyn-(L)-KKKKKK (**4**) and Pyn-(D) kkkkkk (**2**) in water.

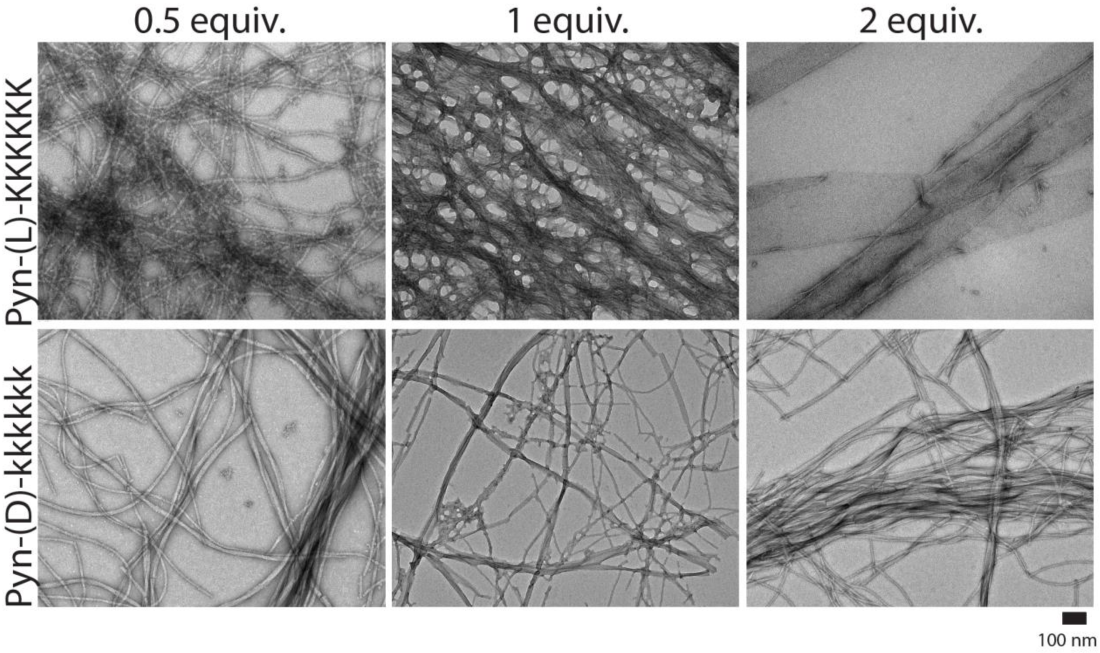

Figure S7. TEM images of 500 μM Pyn-(L)-EEEEEY (**5**) mixed with 0.5, 1, and 2 equivalence of Pyn-(L)-KKKKKK (**4**) and Pyn-(D) kkkkkk (**2**) in water.

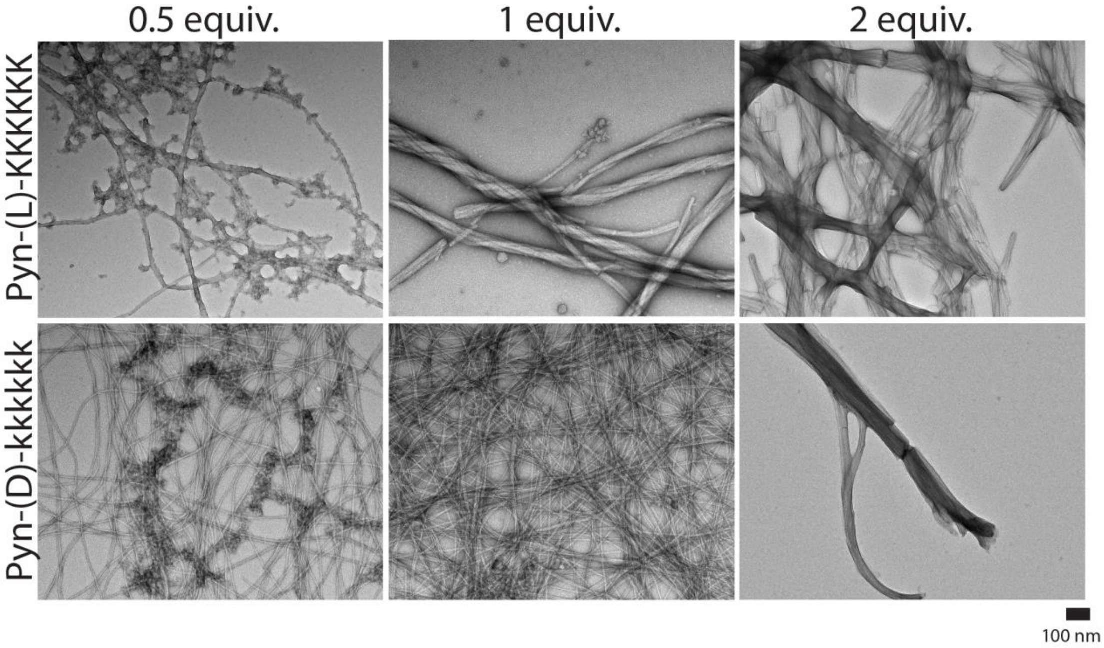

Figure S8. TEM images of 500 μM Pyn-(D)-eeeeey (**6**) mixed with 0.5, 1, and 2 equivalence of Pyn-(L)-KKKKKK (**4**) and Pyn-(D) kkkkkk (**2**) in water.

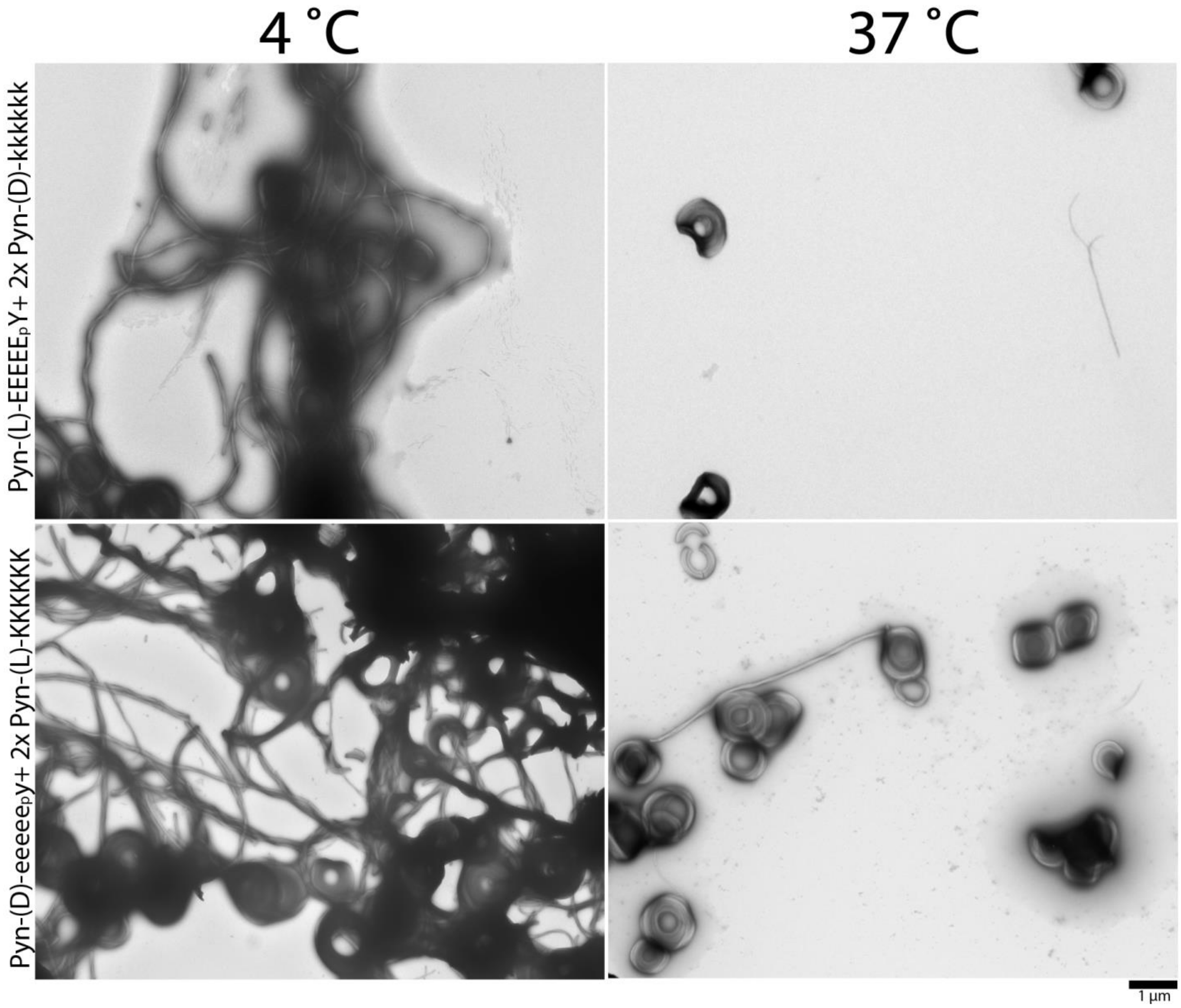

Figure S9. TEM images of 500 µM Pyn-(L)-EEEEE$_p$Y (**1**) and Pyn-(D)-eeeee$_p$y (**3**) mixed with 2 equivalences of Pyn-(D)-kkkkkk (**2**) and Pyn-(L)-KKKKKK (**4**) in water at 4 ˚C and 37 ˚C for 24 h.

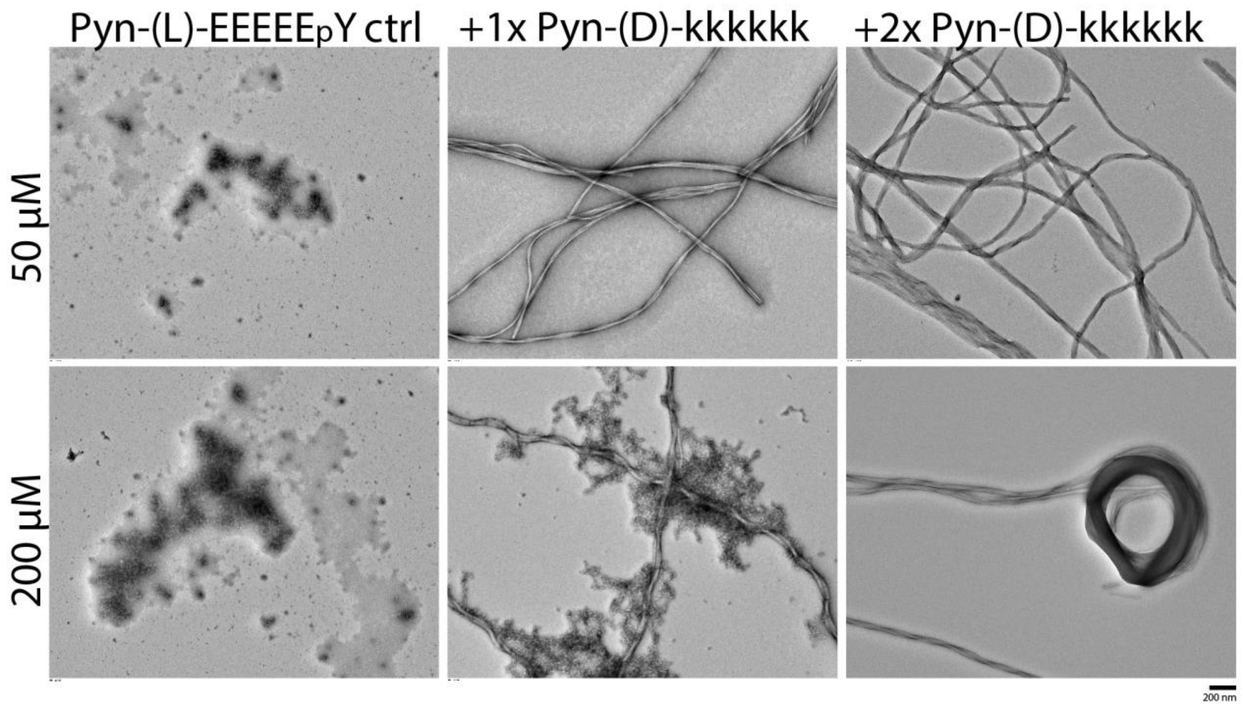

Figure S10. TEM images of 50 and 200 μM Pyn-(L)-EEEEE$_p$Y (**1**) mixed with 1 and 2 equivalences of Pyn-(D)-kkkkkk (**2**) in water for 24 h.

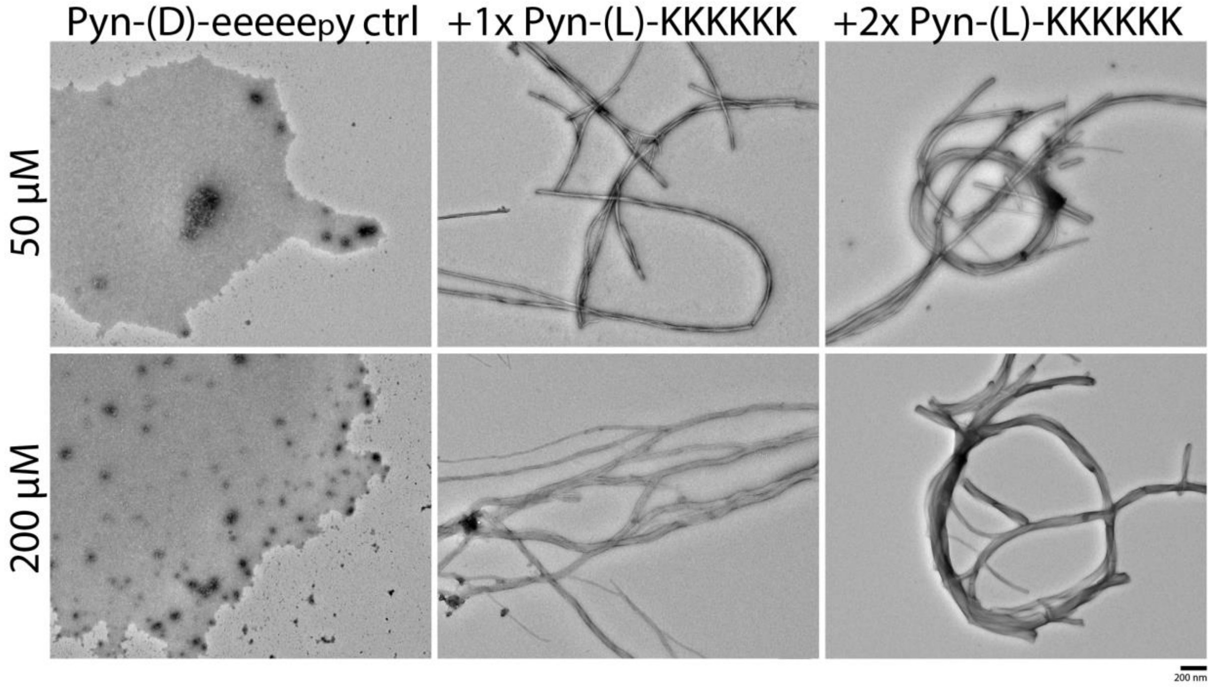

Figure S11. TEM images of 50 and 200 μM Pyn-(D)-eeeee$_p$y (**3**) mixed with 1 and 2 equivalences of Pyn-(L)-KKKKKK (**4**) in water for 24 h.

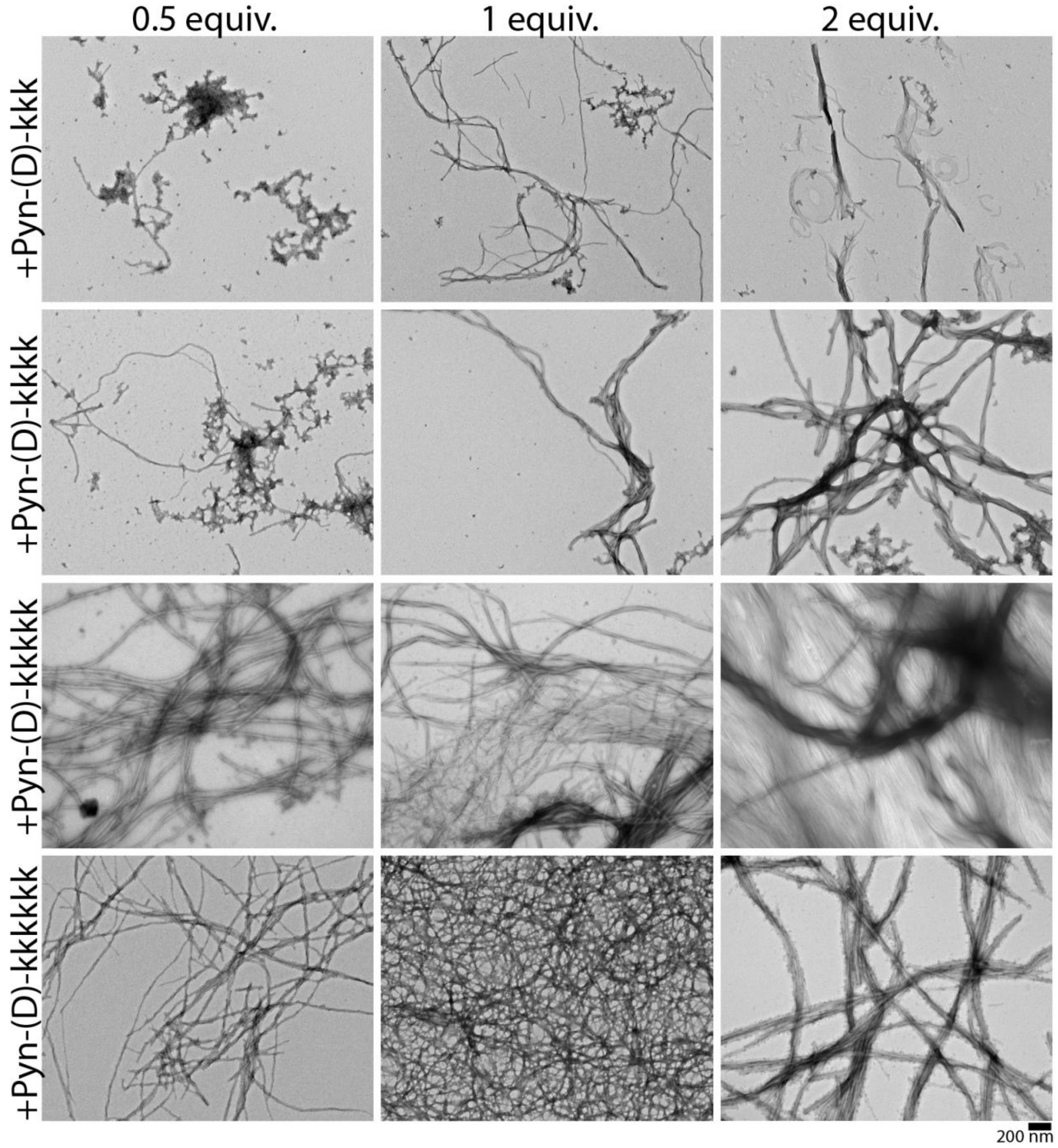

Figure S12. TEM images of 500 μM Pyn-(L)-EEEE$_p$Y (**8**) mixed with 0.5, 1 and 2 equivalences of Pyn-(D)-kkk (**12**), Pyn-(D)-kkkk (**13**), and Pyn-(D)-kkkkk (**14**) in water for 24 h.

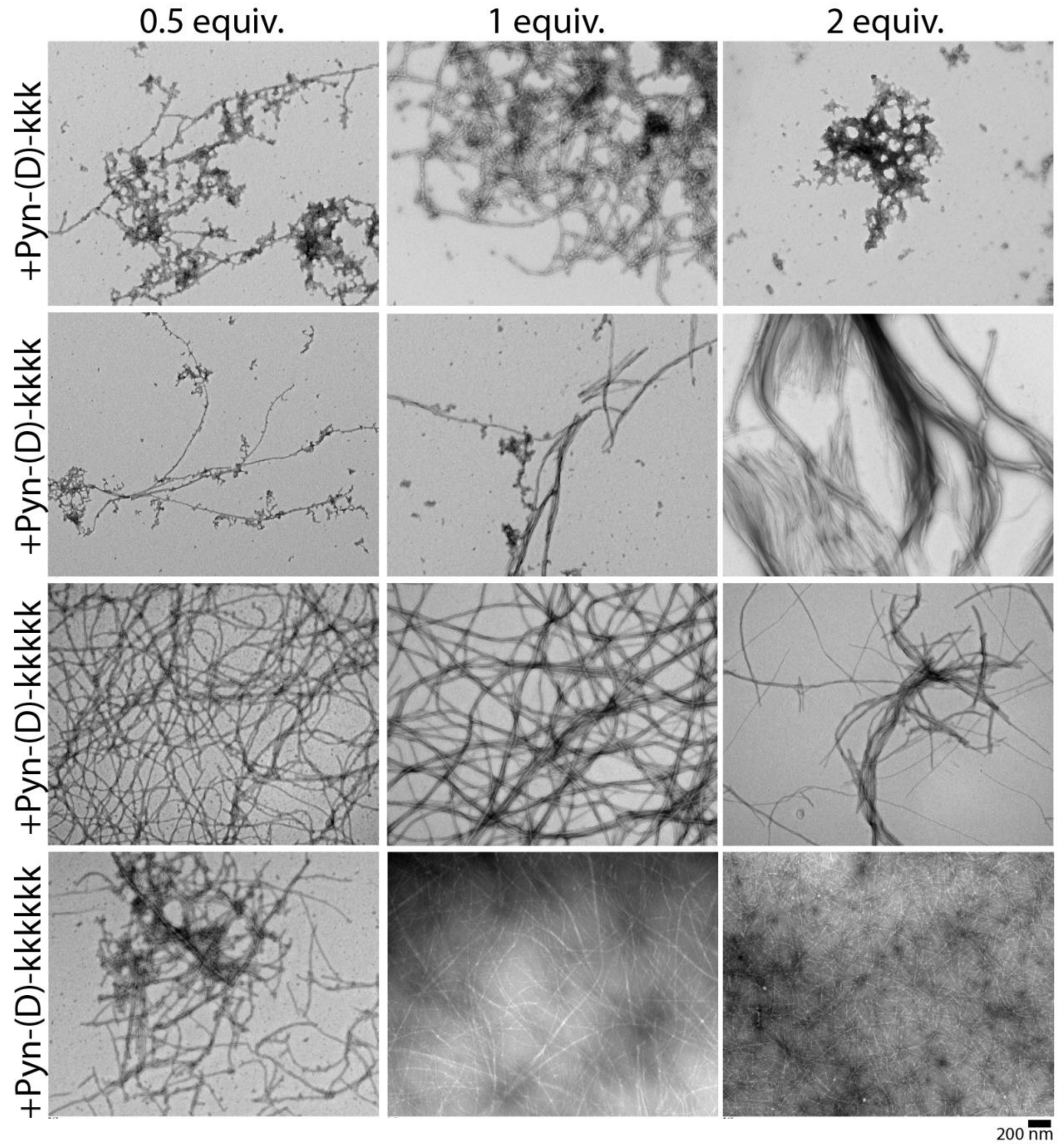

Figure S13. TEM images of 500 μM Pyn-(L)-EEE$_p$Y (**7**) mixed with 0.5, 1 and 2 equivalences of Pyn-(D)-kkk (**12**), Pyn-(D)-kkkk (**13**), and Pyn-(D)-kkkkk (**14**) in water for 24 h.

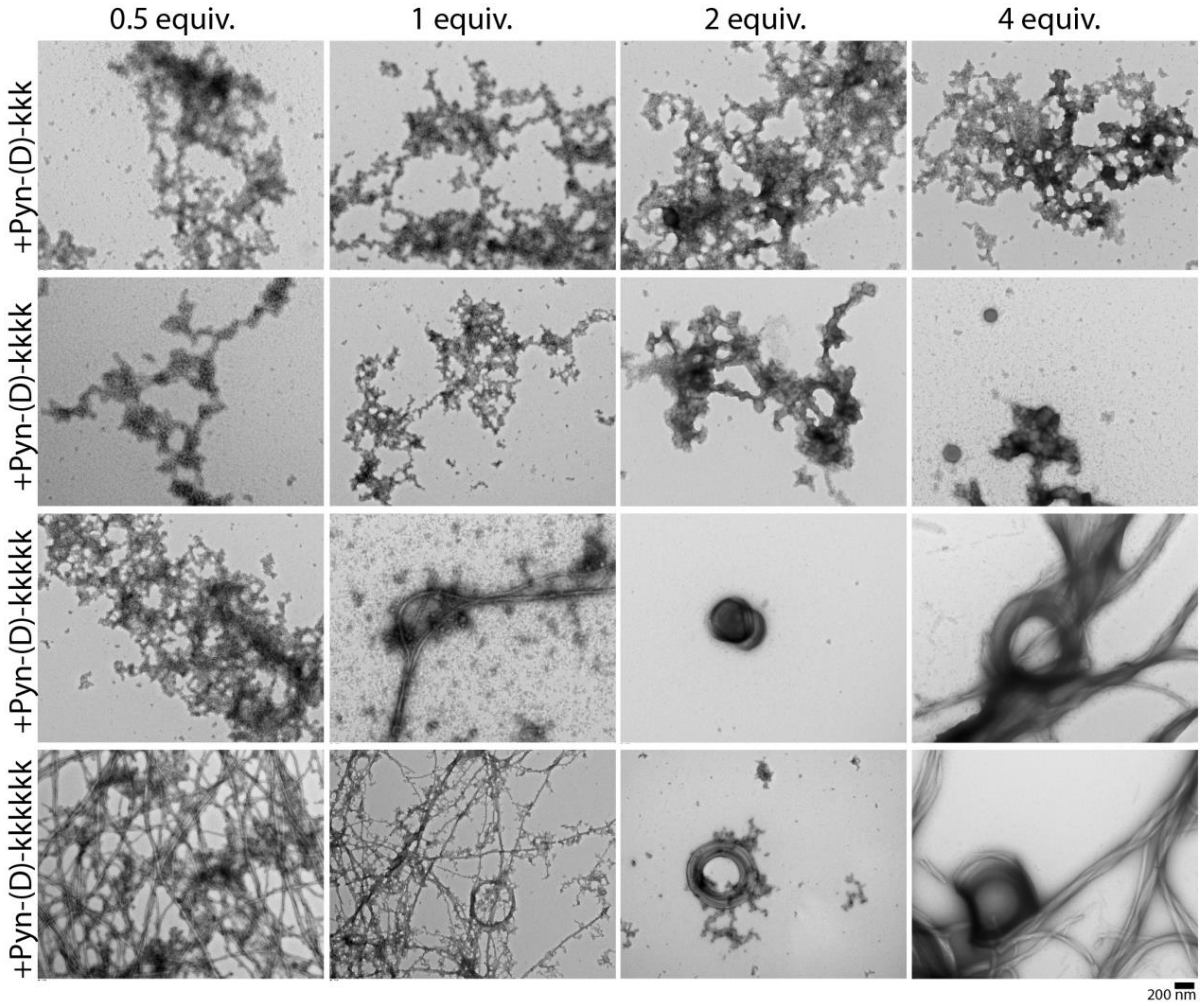

Figure S14. TEM images of 500 μM Pyn-(L)-EEEEE$_{p}$Y (**1**) mixed with 0.5, 1, 2, and 4 equivalence of Pyn-(D)-kkk (**12**), Pyn-(D)-kkkk (**13**), Pyn-(D)-kkkkk (**14**), and Pyn-(D)-kkkkkk (**2**) in water.

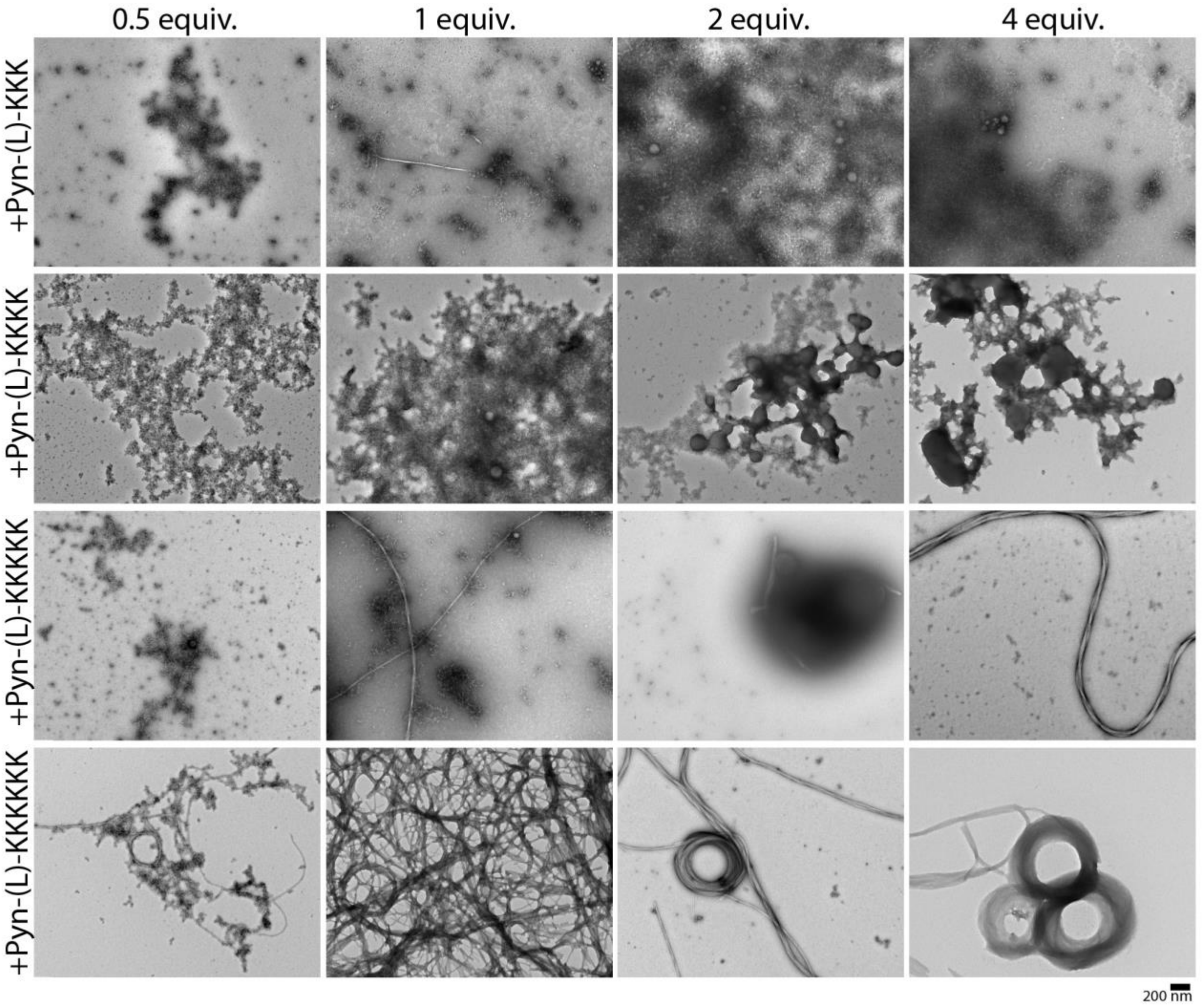

Figure S15. TEM images of 500 μM Pyn-(D)-eeeee$_p$y (**3**) mixed with 0.5, 1, 2, and 4 equivalence of Pyn-(L)-KKK (**9**), Pyn-(L)-KKKK (**10**), Pyn-(L)-KKKKK (**11**), and Pyn-(L)-KKKKKK (**4**) in water.

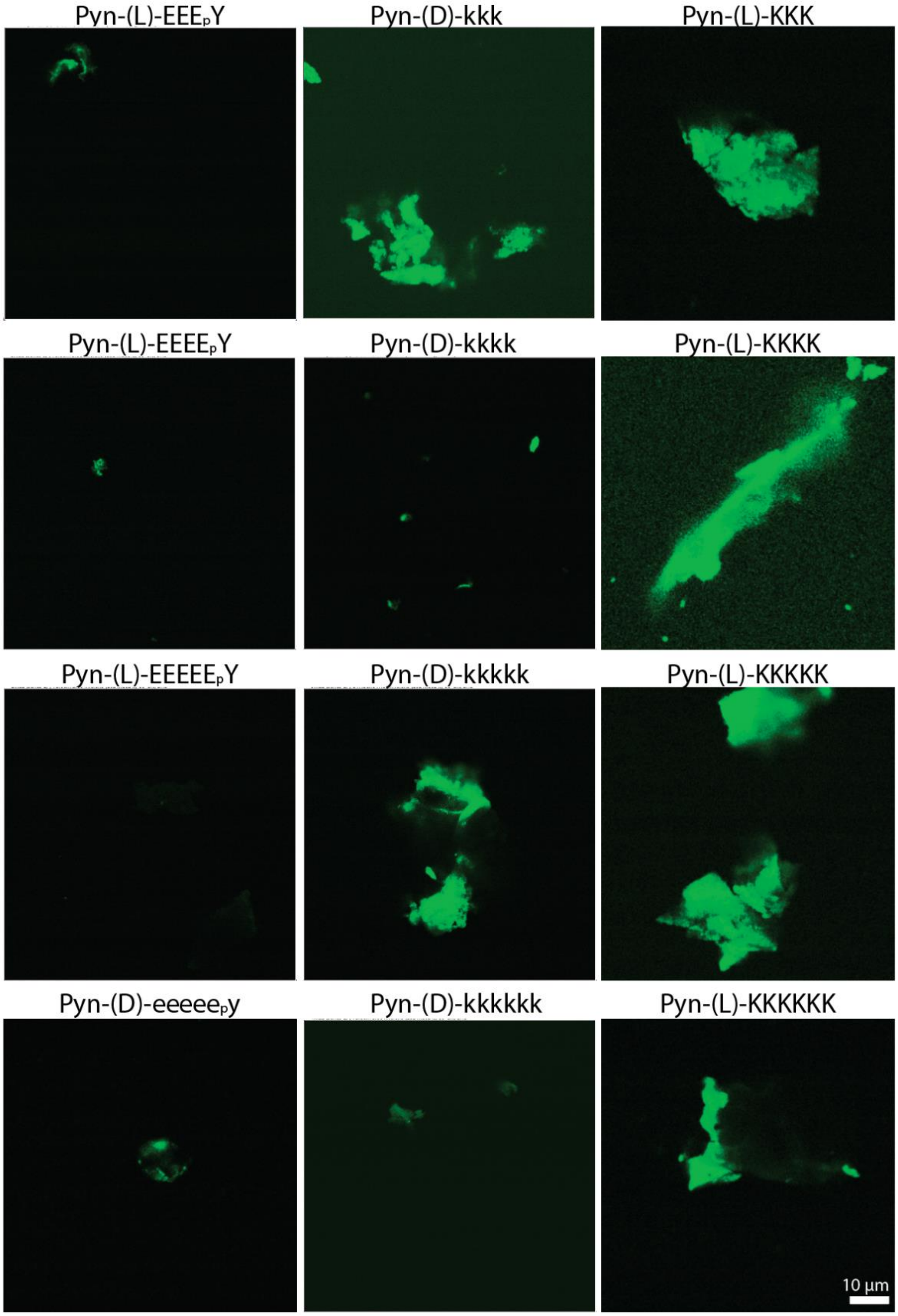

Figure S16. CLSM images of all IDPs at 500 µM in water at pH 7.

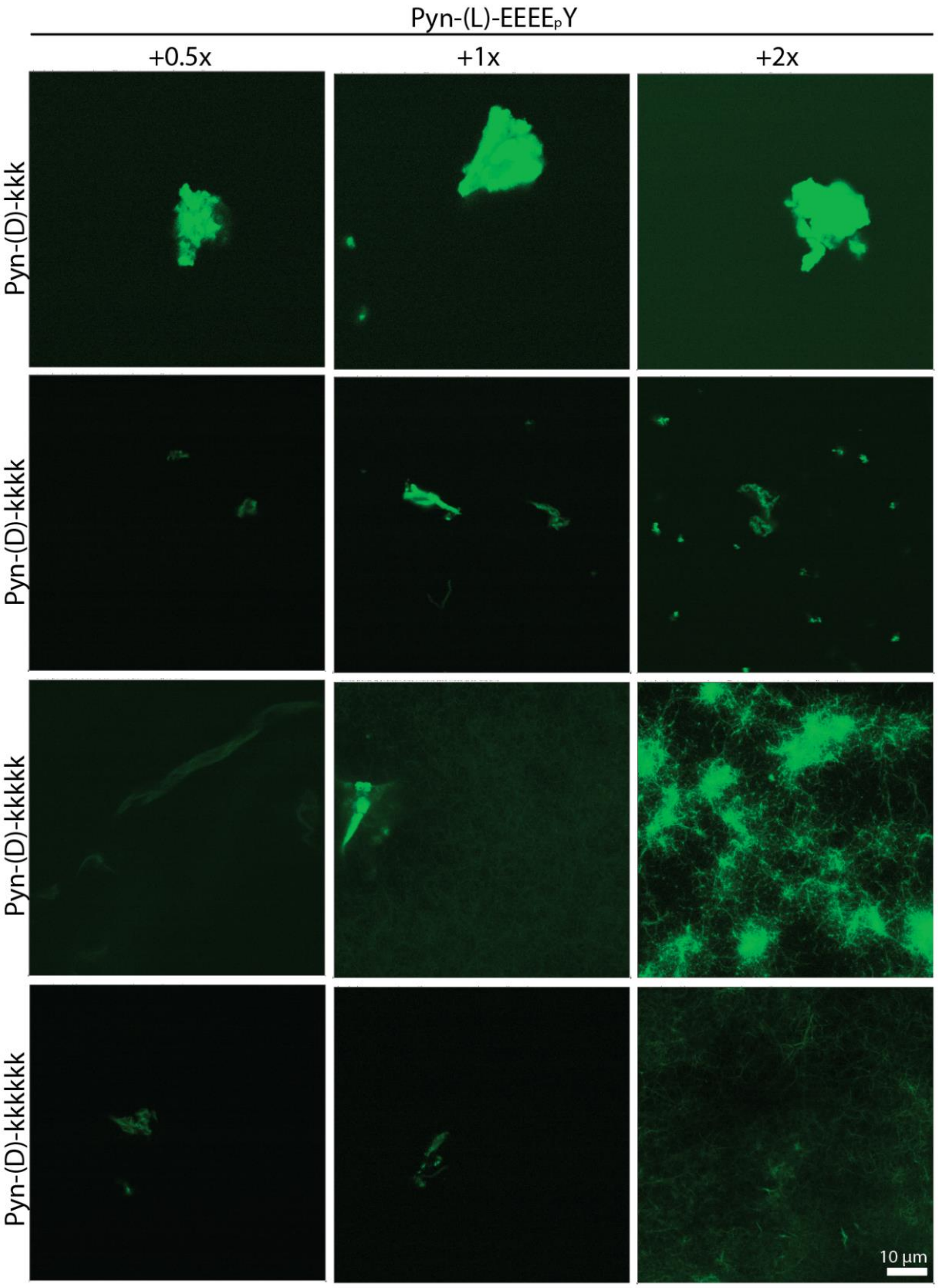

Figure S17. CLSM images of 500 µM Pyn-(L)-EEEE$_p$Y (**8**) mixed with 0.5, 1 and 2 equivalences of Pyn-(D)-kkk (**12**), Pyn-(D)-kkkk (**13**), Pyn-(D)-kkkkk (**14**), and Pyn-(D)-kkkkkk (**2**) in water for 24 h.

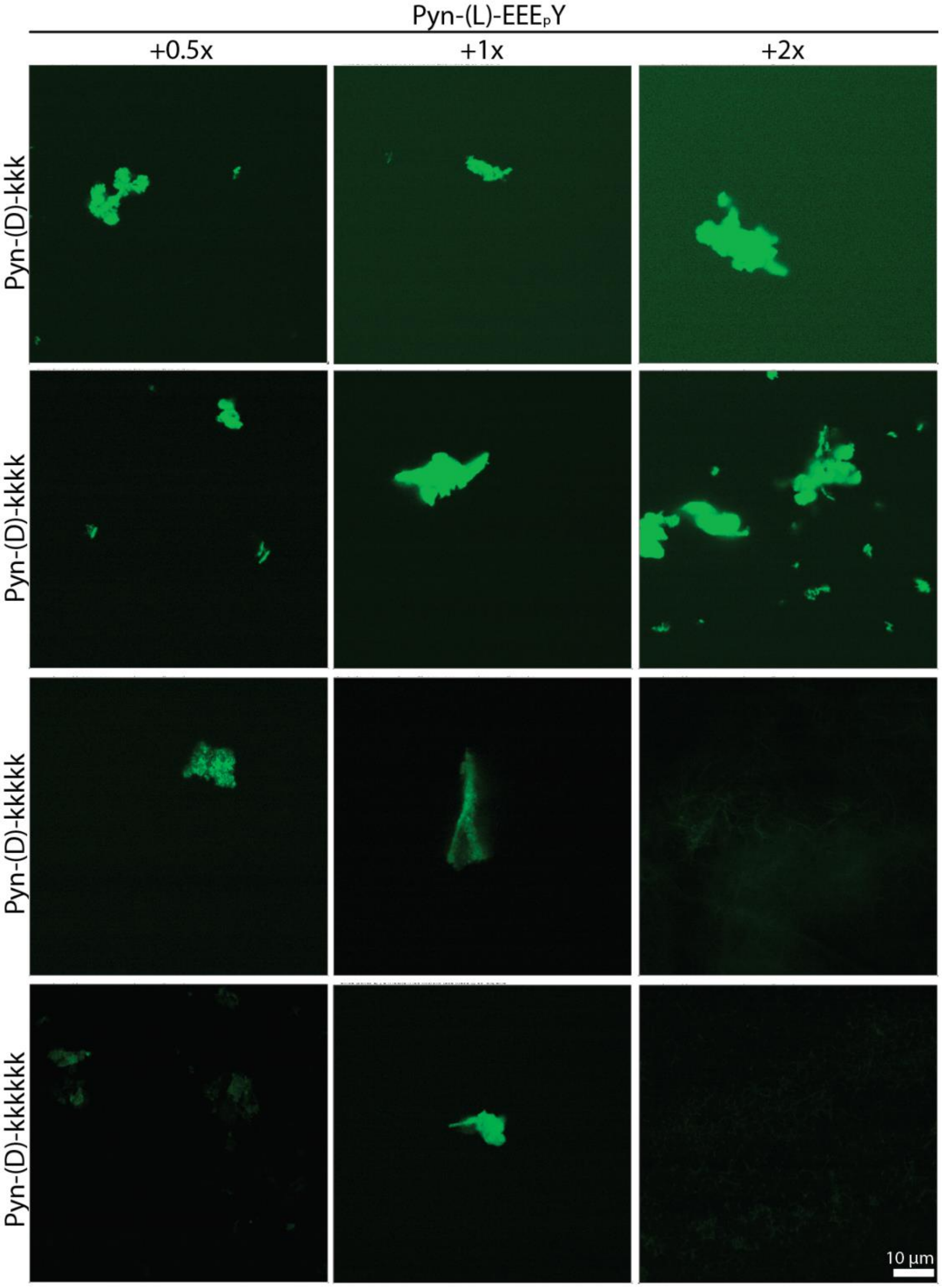

Figure S18. CLSM images of 500 μM Pyn-(L)-EEE$_p$Y (**7**) mixed with 0.5, 1 and 2 equivalences of Pyn-(D)-kkk (**12**), Pyn-(D)-kkkk (**13**), Pyn-(D)-kkkkk (**14**), and Pyn-(D)-kkkkkk (**2**) in water for 24 h.

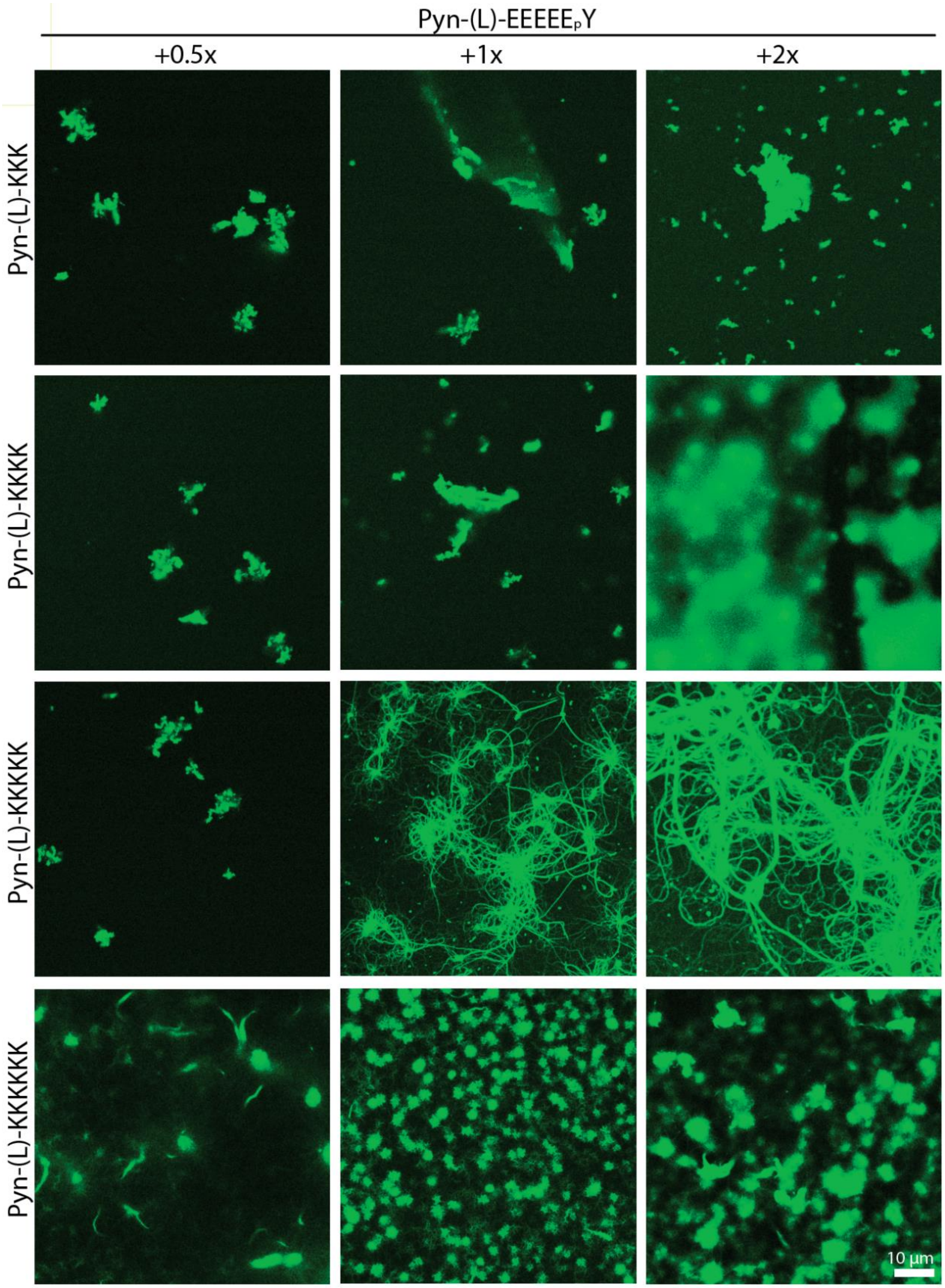

Figure S19. CLSM images of 500 μM Pyn-(L)-EEEEE$_p$Y (**1**) mixed with 0.5, 1 and 2 equivalences of Pyn-(L)-KKK (**9**), Pyn-(L)-KKKK (**10**), Pyn-(L)-KKKKK (**11**) and Pyn-(L)-KKKKKK (**4**) in water for 24 h.

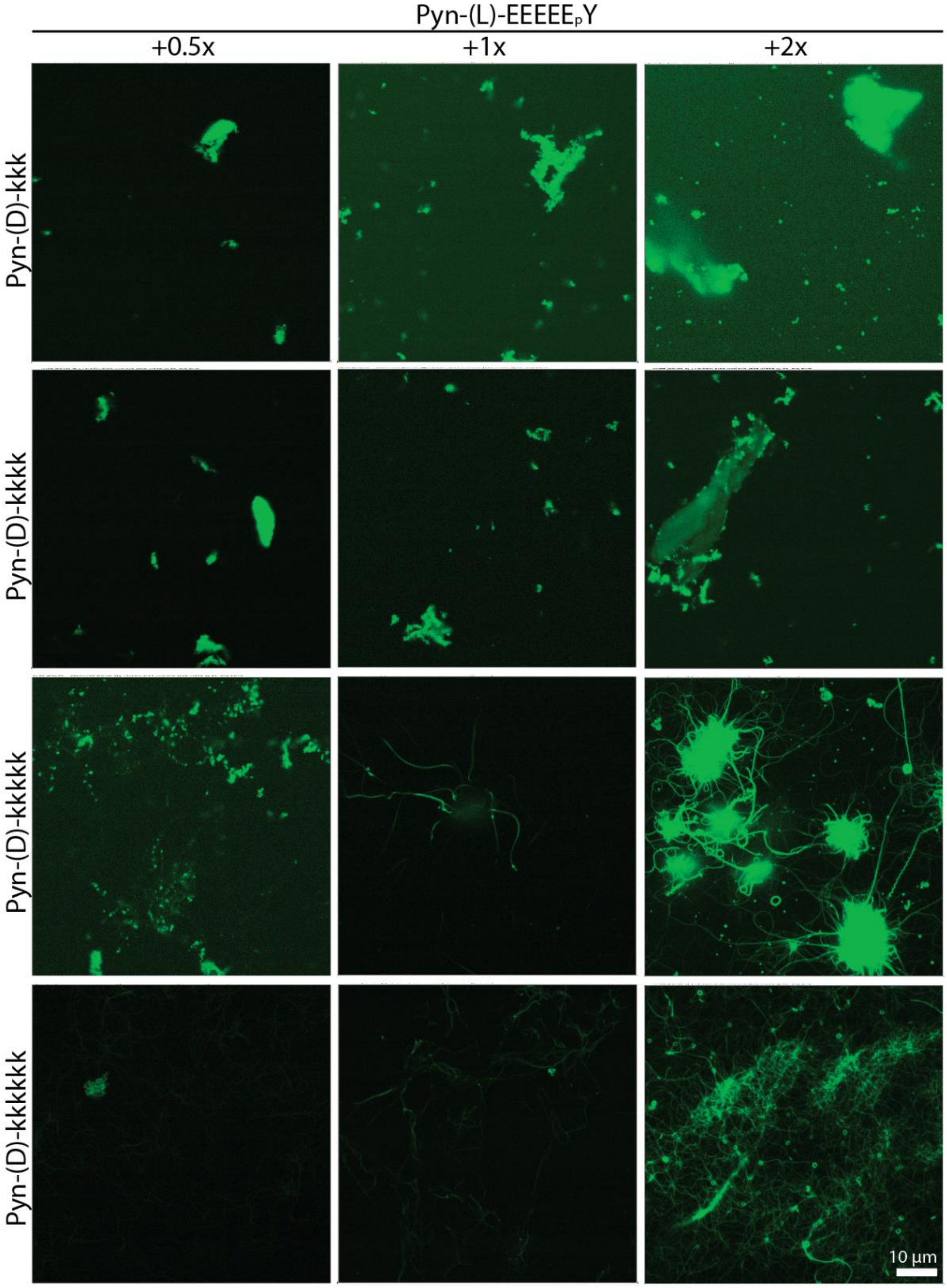

Figure S20. CLSM images of 500 μM Pyn-(L)-EEEEE$_p$Y (**1**) mixed with 0.5, 1 and 2 equivalences of Pyn-(D)-kkk (**12**), Pyn-(D)-kkkk (**13**), Pyn-(D)-kkkkk (**14**), and Pyn-(D)-kkkkkk (**2**) in water for 24 h.

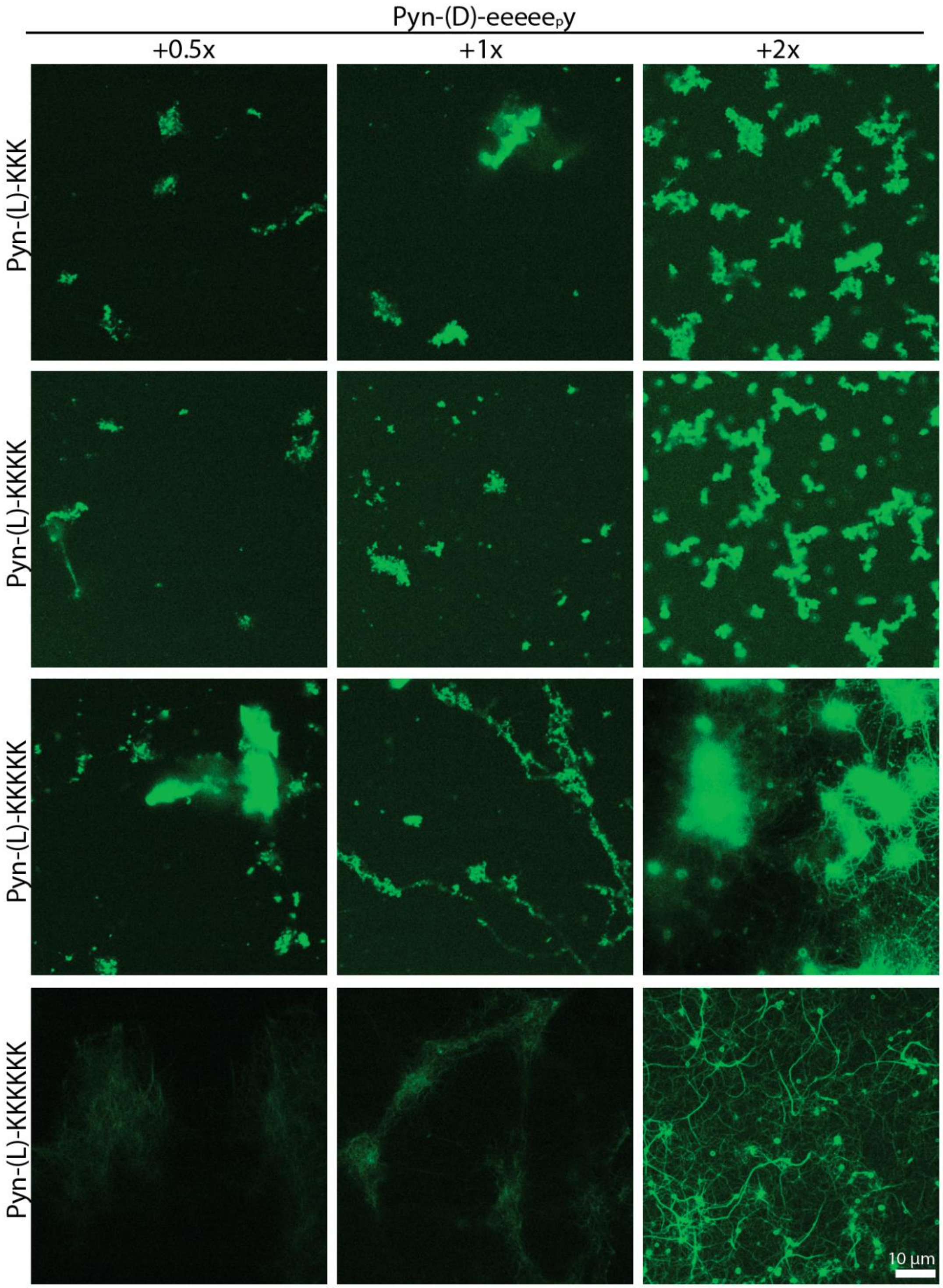

Figure S21. CLSM images of 500 µM Pyn-(D)-eeeee$_{p}$y (**3**) mixed with 0.5, 1 and 2 equivalences of Pyn-(L)-KKK (**9**), Pyn-(L)-KKKK (**10**), Pyn-(L)-KKKKK (**11**) and Pyn-(L)-KKKKKK (**4**) in water for 24 h.

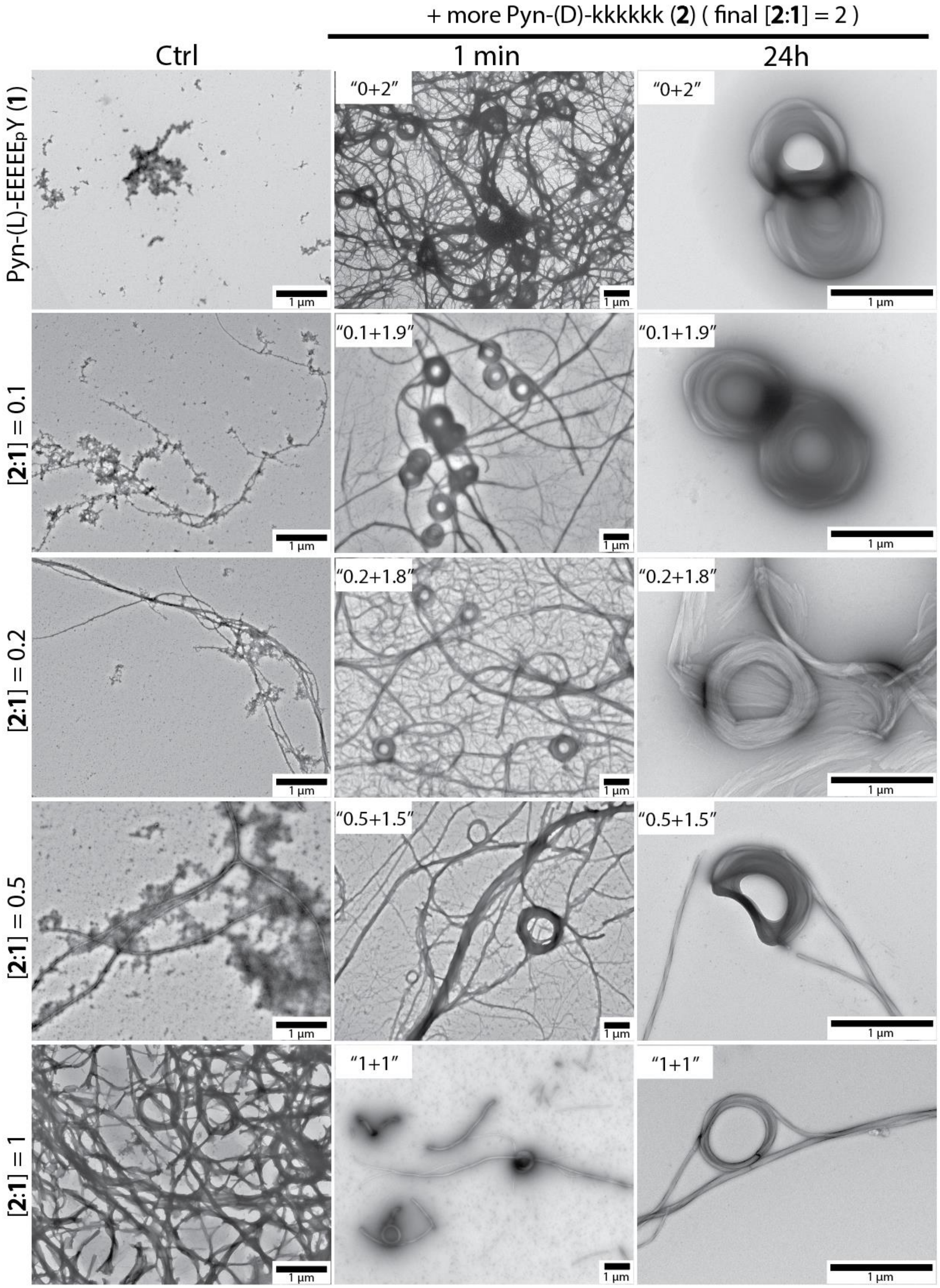

Figure S22. TEM images of 1 mM Pyn-(L)-EEEEE$_p$Y (**1**) mixed with 0, 0.1, 0.2, 0.5, and 1 equivalents of Pyn-(D)-kkkkkk (**2**) incubated for 24 h at 37 °C in pH 7 water. Additional

volumes of **2** were then added to achieve 2, 1.9, 1.8, 1.5, and 1 equivalents, respectively—designated as “0+2,” “0.1+1.9,” “0.2+1.8,” “0.5+1.5,” and “1+1.” All final mixtures had 500 μM of **1** with 2 equivalents of **2** and were incubated for 1 min and 24 h at 37 °C before imaging.

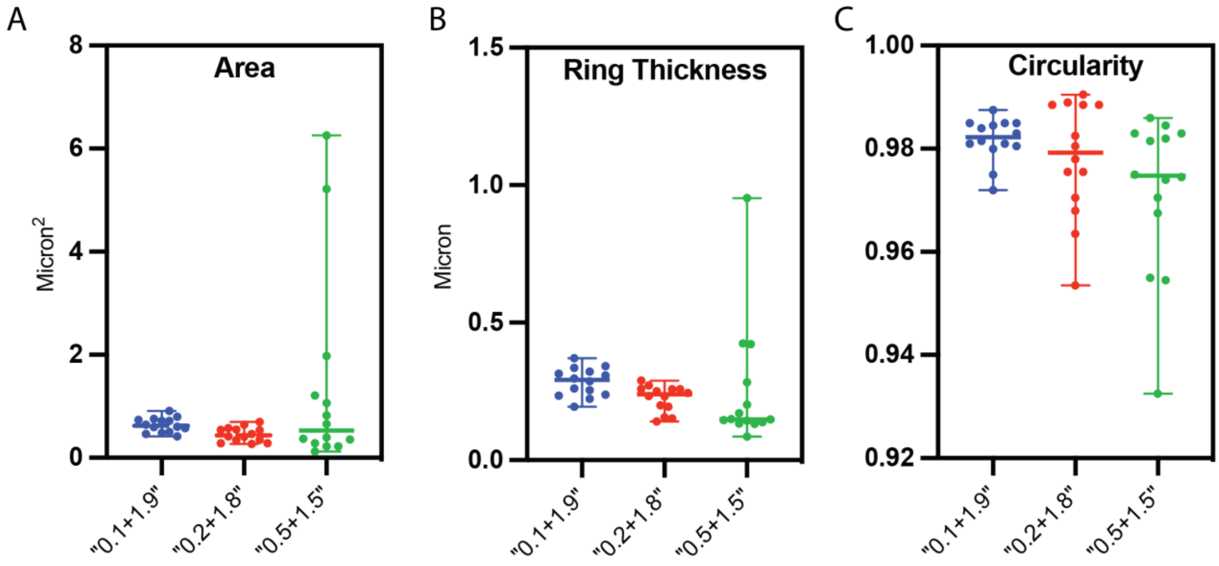

Figure S23. Area, ring thickness, and circularity measurements for TEM images in **Figure S22,** 1 min mixture results. Data analyzed using image J.

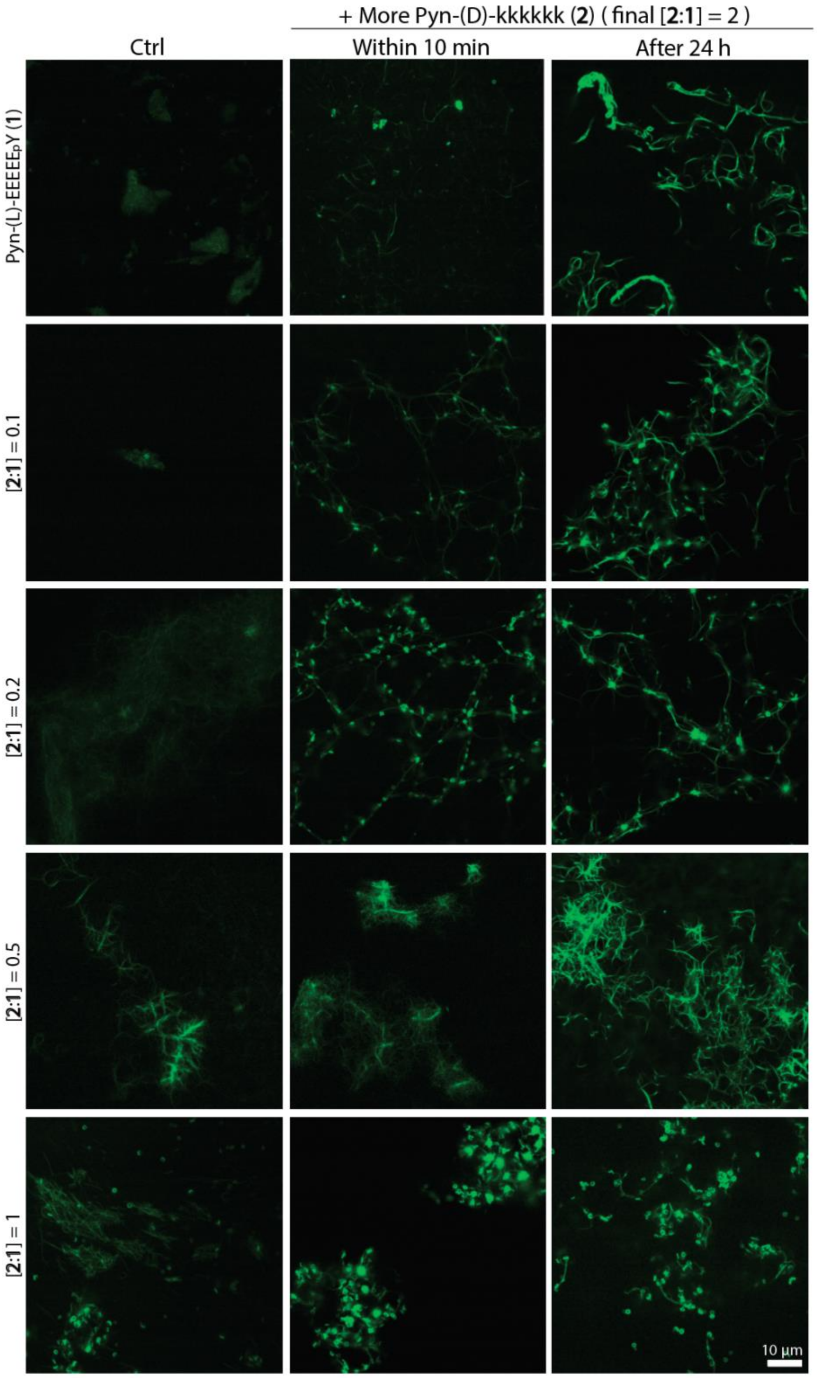

Figure S24. CLSM images of 1 mM Pyn-(L)-EEEEE$_p$Y (**1**) mixed with 0, 0.1, 0.2, 0.5, and 1 equivalents of Pyn-(D)-kkkkkk (**2**) incubated for 24 h at 37 °C in pH 7 water. Additional volumes of **2** were then added to achieve 2, 1.9, 1.8, 1.5, and 1 equivalents. All final

mixtures had 500 μM of **1** with 2 equivalents of **2** and were incubated within 10 min and after 24 h at 37 °C before imaging.

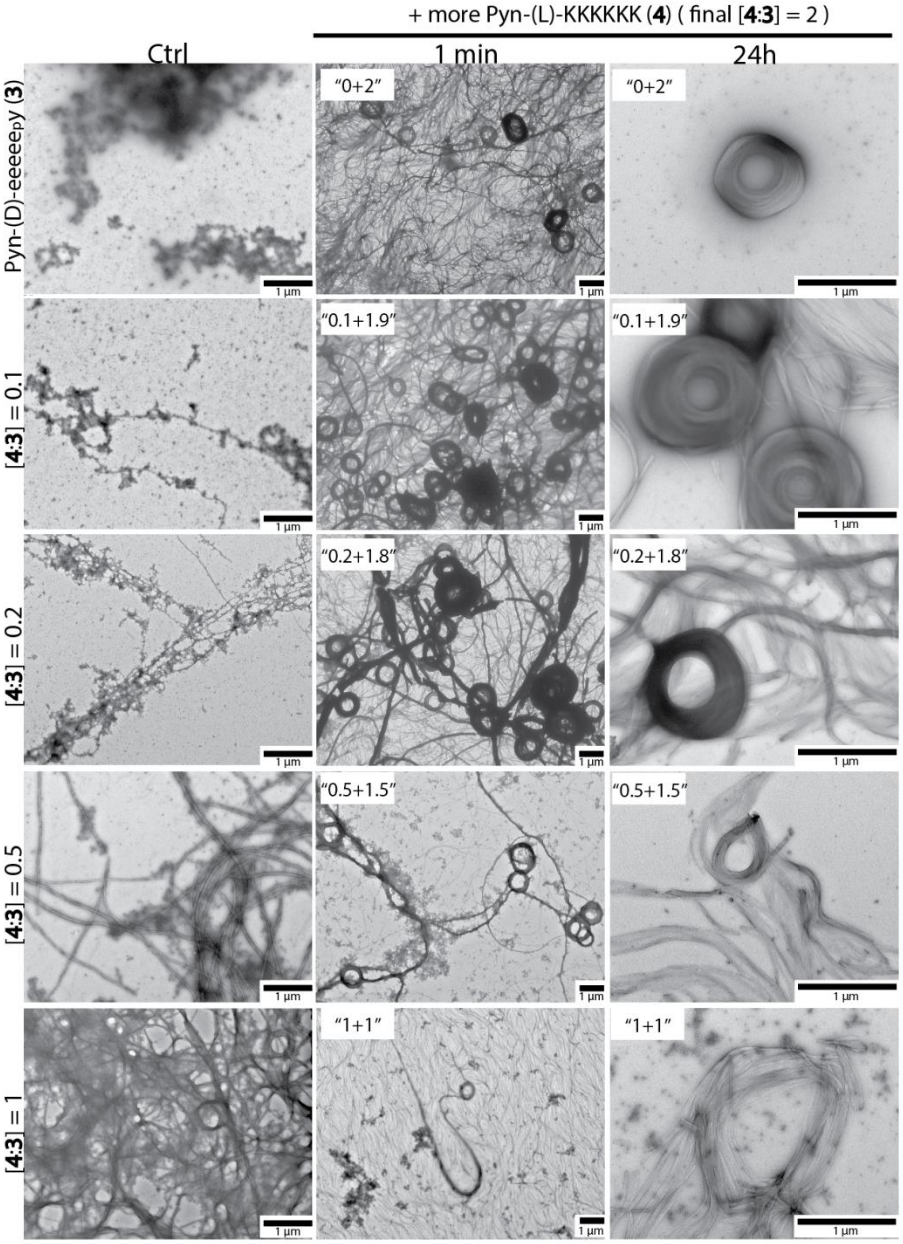

Figure S25. TEM images of 1 mM Pyn-(D)-eeeee$_{p}$y (**3**) mixed with 0, 0.1, 0.2, 0.5, and 1 equivalents of Pyn-(L)-KKKKKK (**4**) incubated for 24 h at 37 °C in pH 7 water. Additional volumes of Pyn-(L)-KKKKKK (**4**) were then added to achieve 2, 1.9, 1.8, 1.5, and 1 equivalents, respectively, resulting in a final concentration of 500 μM Pyn-(D)-eeeee$_{p}$y (**3**)

with 2 equivalents of Pyn-(L)-KKKKKK (**4**). The mixtures were then incubated for 1 min and 24 h at 37 °C.

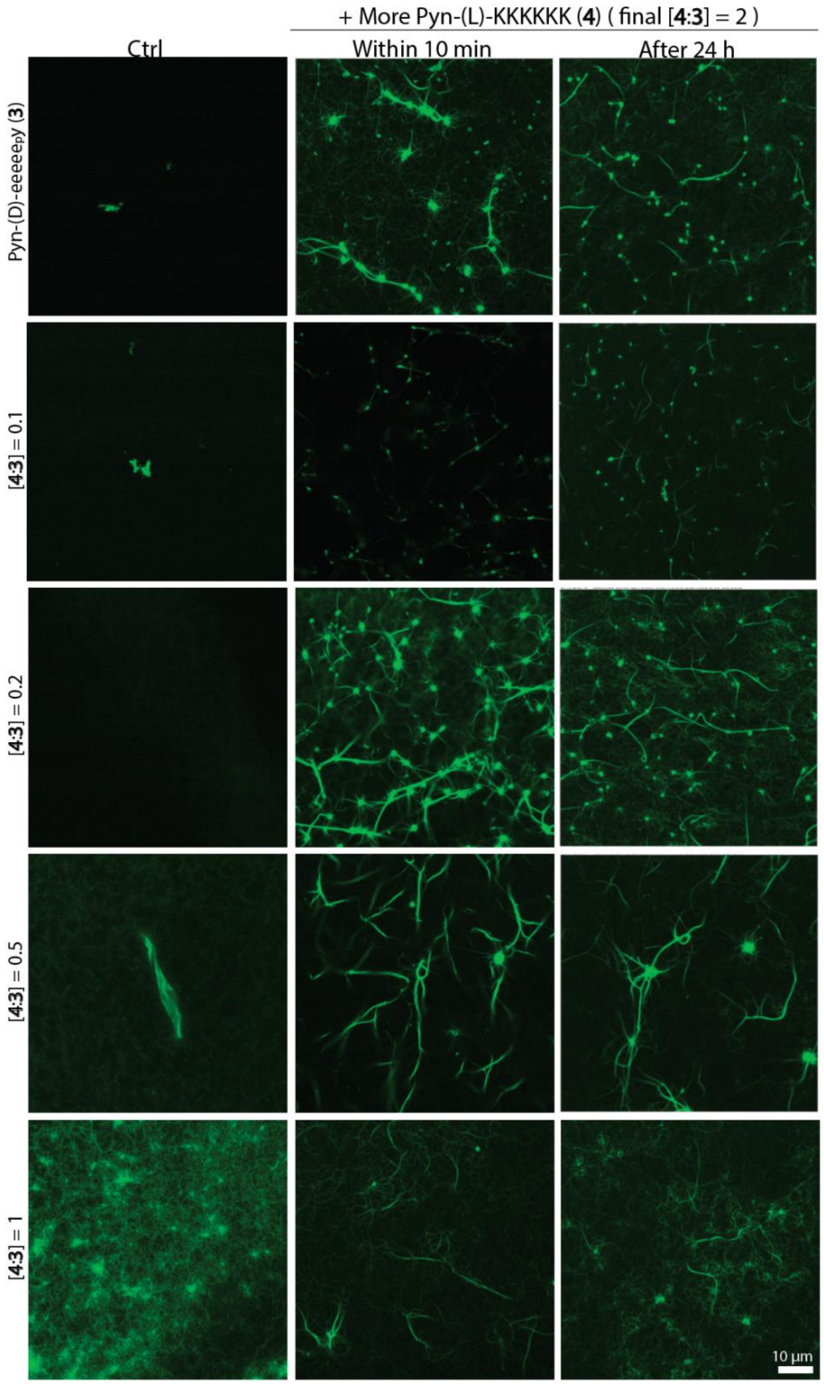

Figure S26. CLSM images of 1 mM Pyn-(D)-eeeee$_p$y (**3**) mixed with 0, 0.1, 0.2, 0.5, and 1 equivalents of Pyn-(L)-KKKKKK (**4**) incubated for 24 h at 37 °C in pH 7 water. Additional

volumes of **4** were then added to achieve 2, 1.9, 1.8, 1.5, and 1 equivalents. All final mixtures had 500 μM of **3** with 2 equivalents of **4** and were incubated within 10 min and after 24 h at 37 °C before imaging.

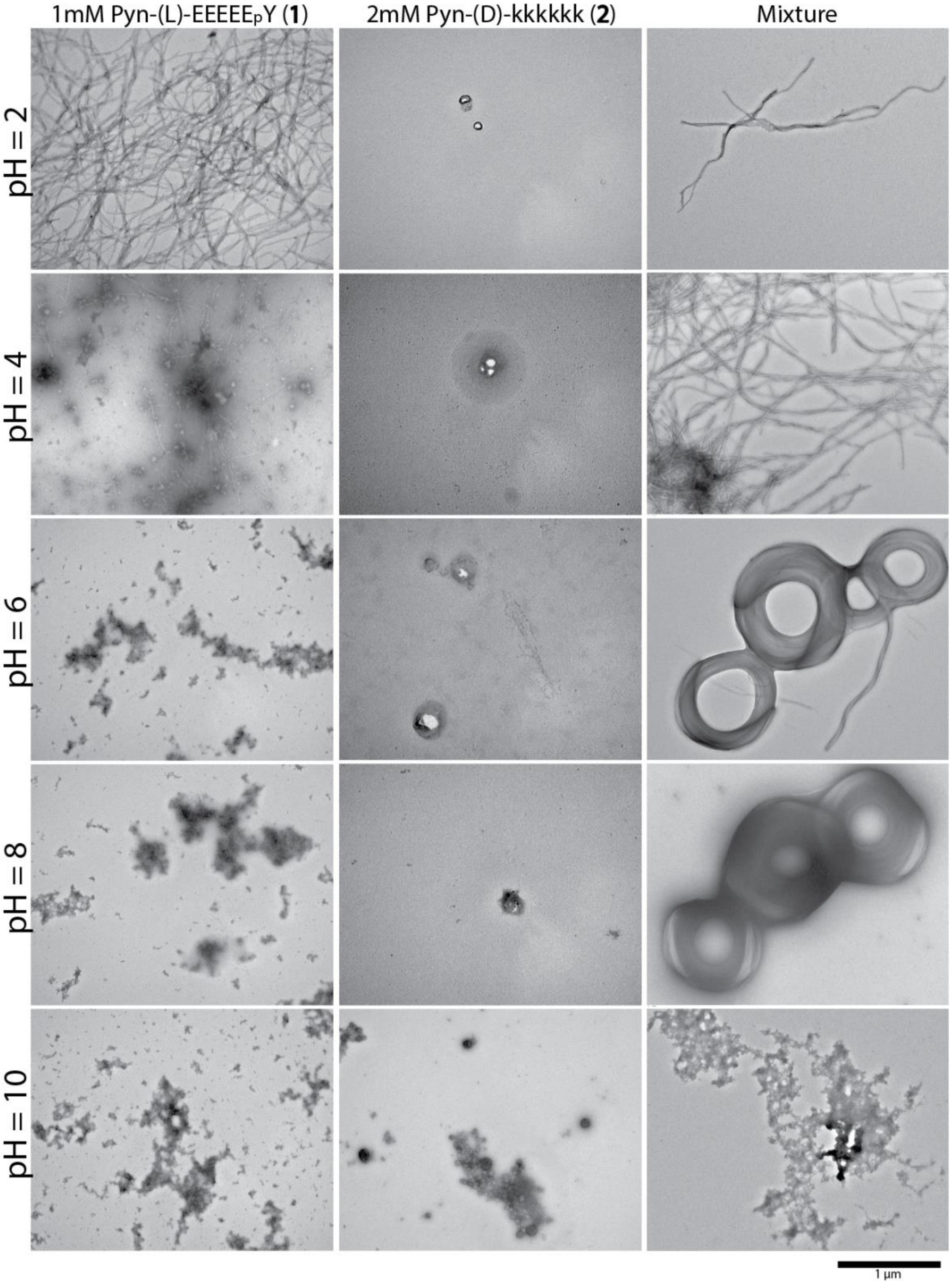

Figure S27. TEM images of 1 mM Pyn-(L)-EEEEE$_p$Y (**1**), 2mM Pyn-(D)-kkkkkk (**2**) and their mixture 500 μM Pyn-(L)-EEEEE$_p$Y (**1**) with 2 equivalents of Pyn-(D)-kkkkkk (**2**) incubated for 24 h at 37 °C in water at pH 2, 4, 6, 8, and 10.

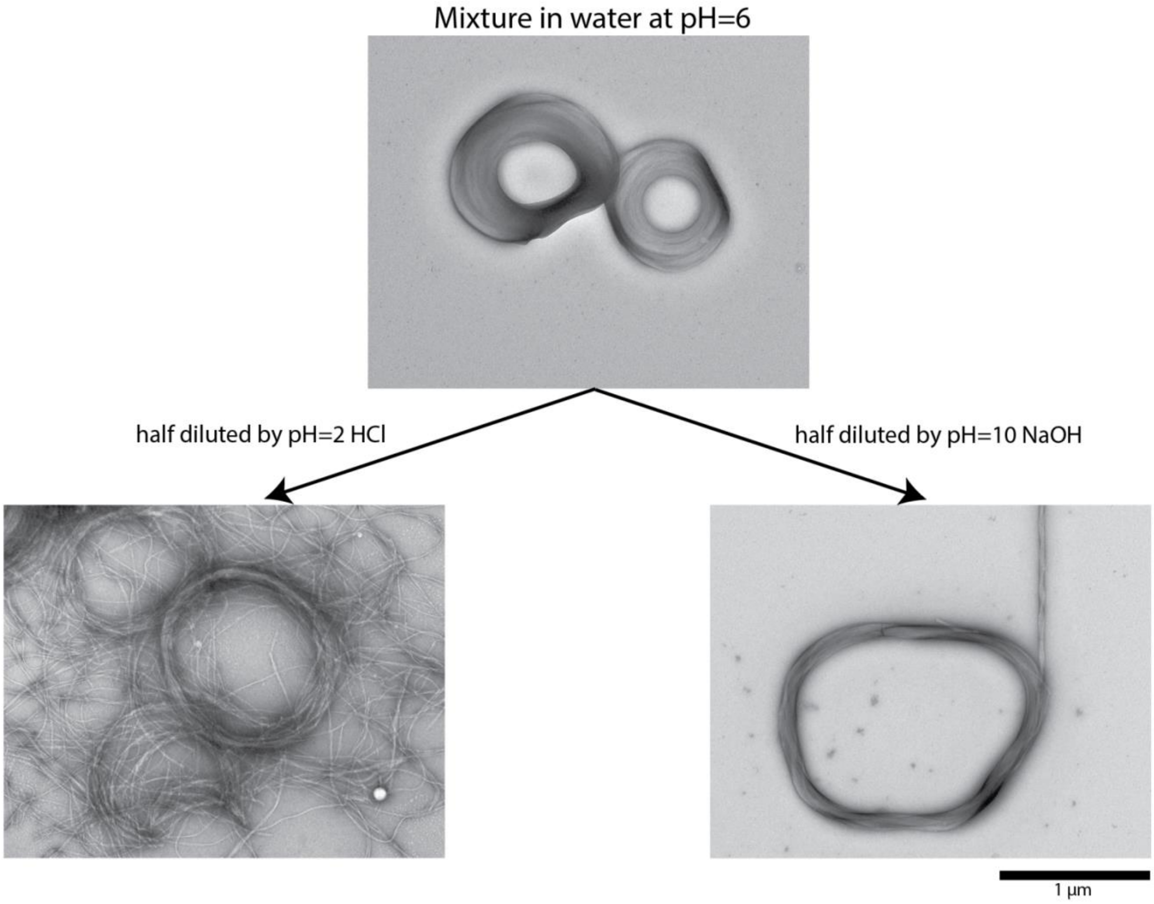

Figure S28. TEM images of 1 mM Pyn-(L)-EEEEE$_p$Y (**1**) with 2 equivalents of Pyn-(D)-kkkkkk (**2**) in water at pH=6 and diluted by half with pH=2 HCl and pH=10 NaOH for 24 h at 37 °C in water.

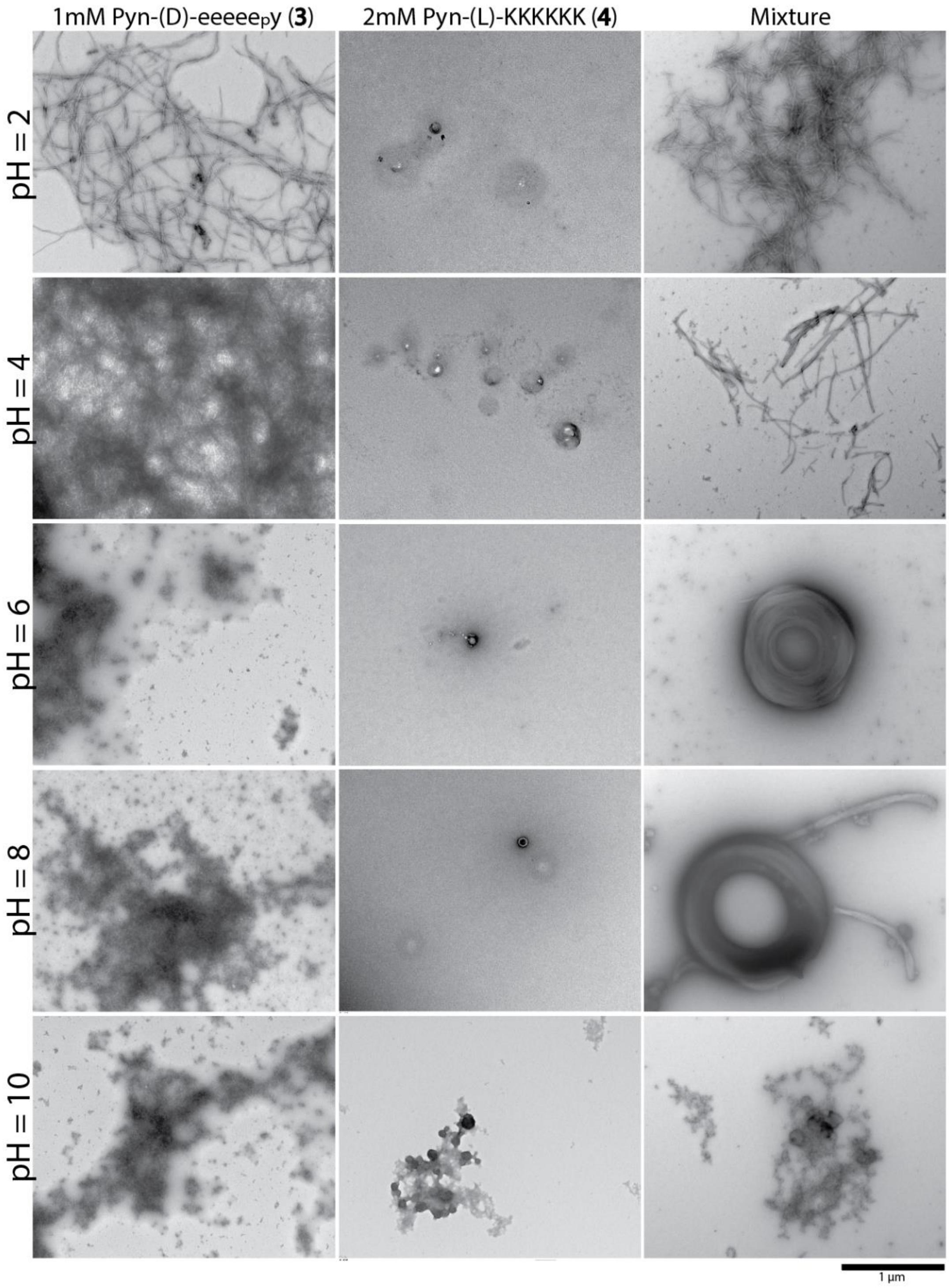

Figure S29. TEM images of 1 mM Pyn-(D)-eeeee$_p$y (**3**), 2mM Pyn-(L)-KKKKKK (**4**) and their mixture 500 µM Pyn-(D)-eeeee$_p$y (**3**) with 2 equivalents of Pyn-(L)-KKKKKK (**4**) incubated for 24 h at 37 °C in water at pH 2, 4, 6, 8, and 10.

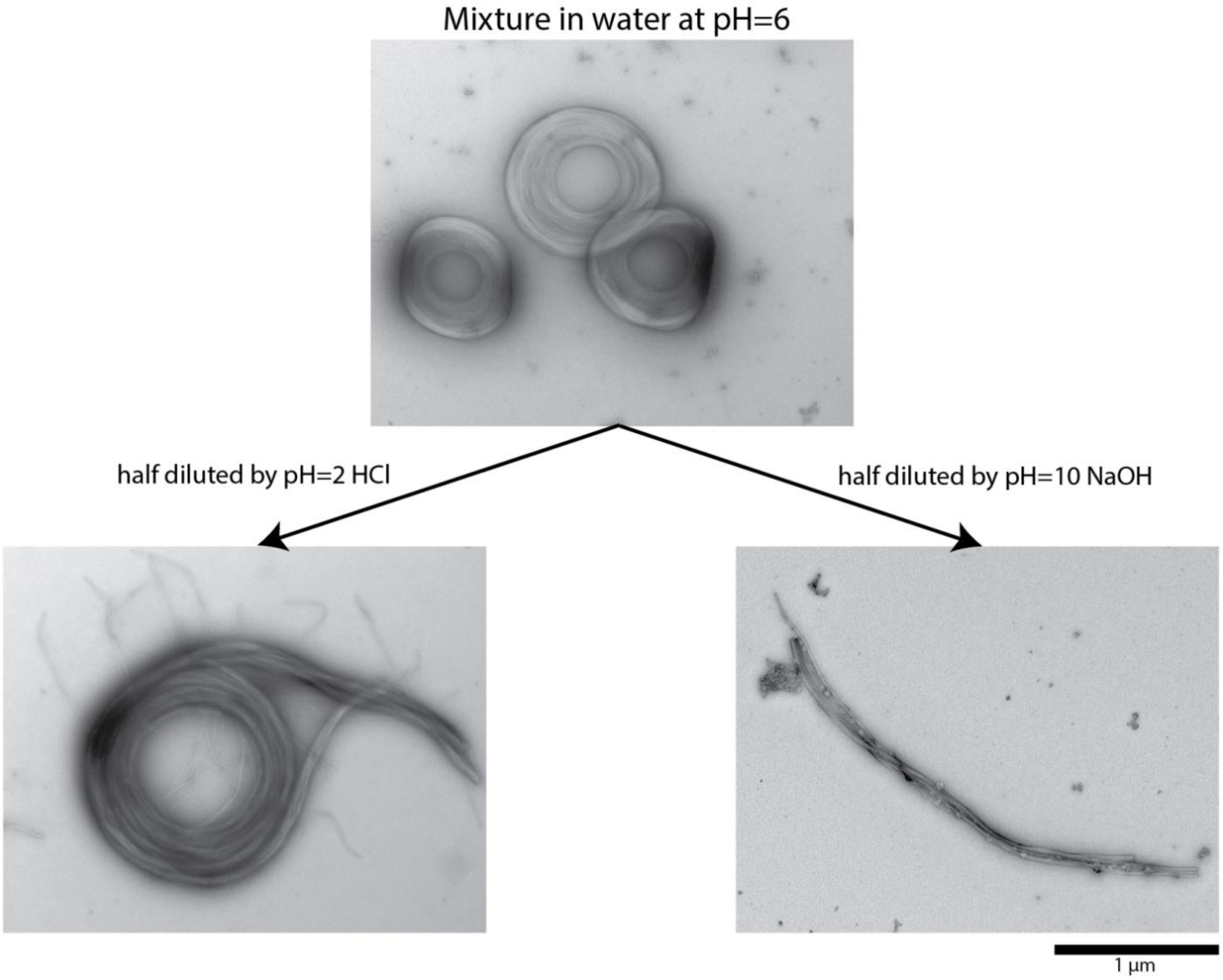

Figure S30. TEM images of 1 mM Pyn-(D)-eeeee$_p$y (**3**) with 2 equivalents of Pyn-(L)-KKKKKK (**4**) in water at pH=6 and diluted by half with pH=2 HCl and pH=10 NaOH for 24 h at 37 °C in water.

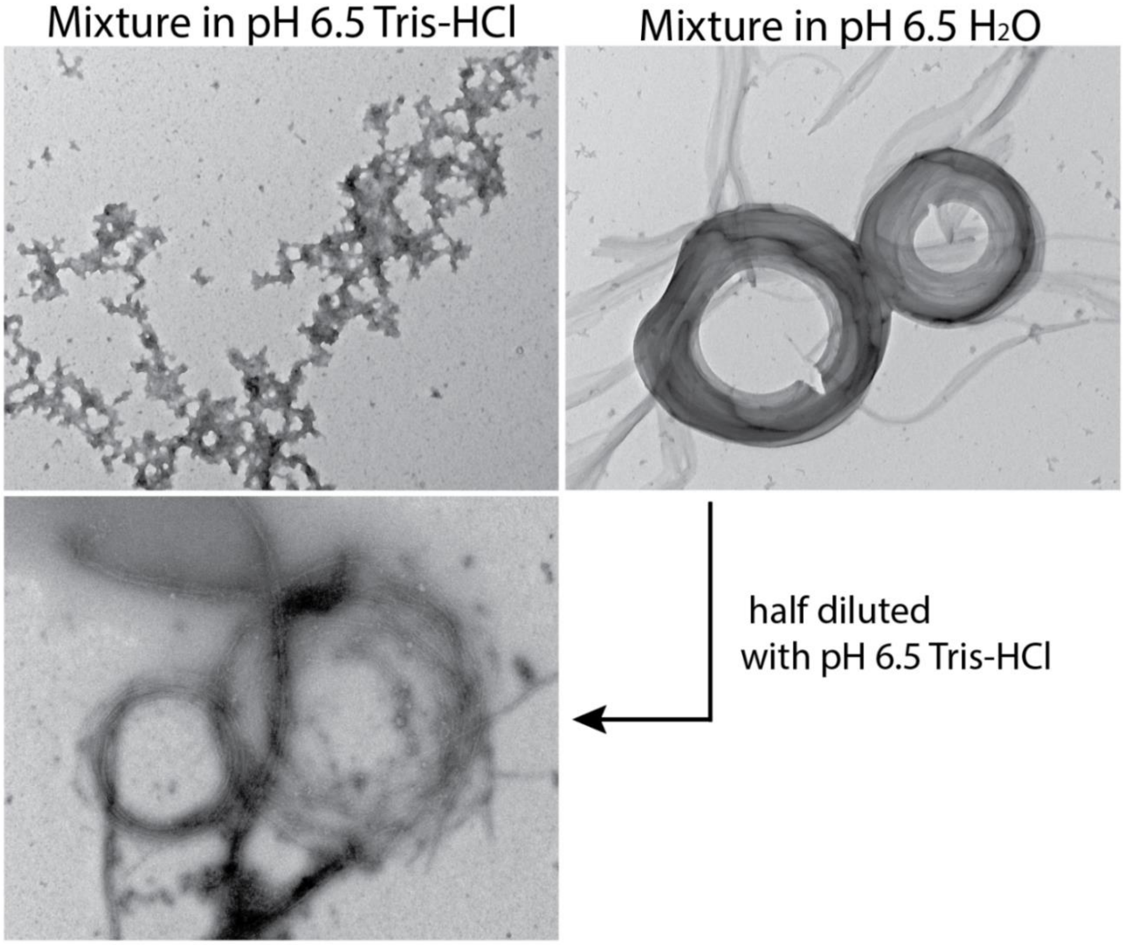

Figure S31. TEM images of 500 μM Pyn-(D)-eeeee$_p$y (**3**) with 2 equivalents of Pyn-(L)-KKKKKK (**4**) in Tris-HCl buffer and water at pH=6.5 and diluted by half with pH=6.5 1M Tris-HCl for 24 h at 37 °C.

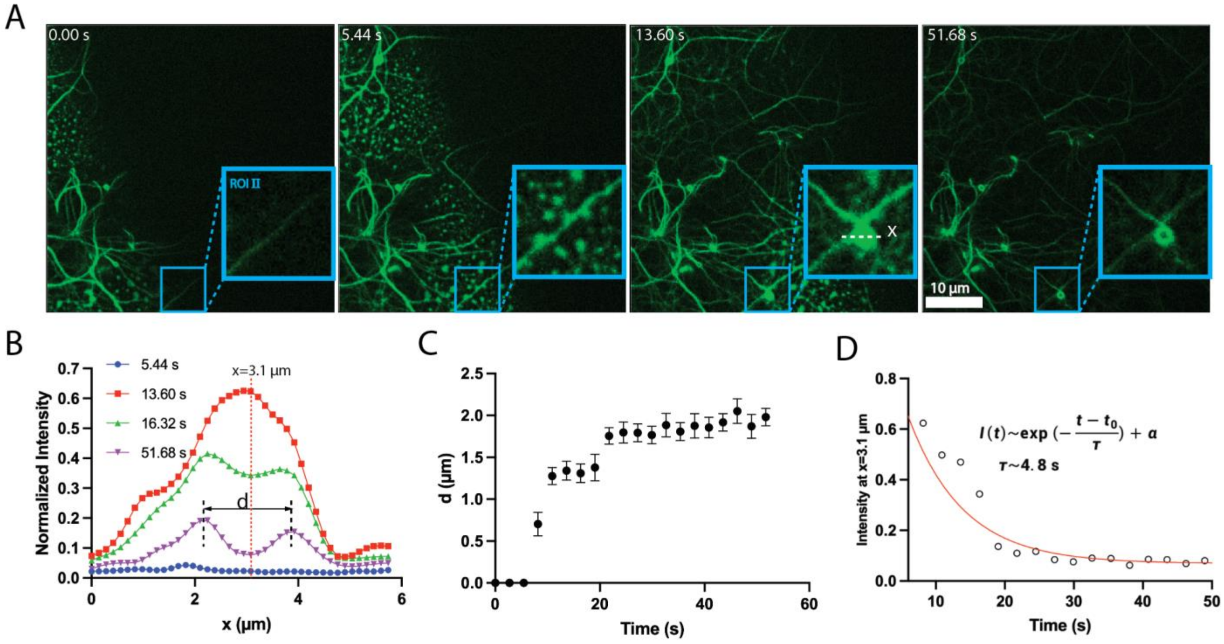

Figure S32. (A) Image sequences of ring formation during reactions. Highlighted ROI II exhibits nucleation on the fibers followed by the ring formation. (B) Time evolution of the normalized intensity profiles on the line in the third panel in ROI II. Two peaks from 16.32 s indicate the ring shapes with a hole at the center. (C) The size of the hole, d, is rapidly saturated near 2 μm, consistently with the decaying of the intensity at x=3.1 μm. (D) The intensity at x=3.1 μm decays exponentially and the fitted lifetime (τ) is about 4.8 s.

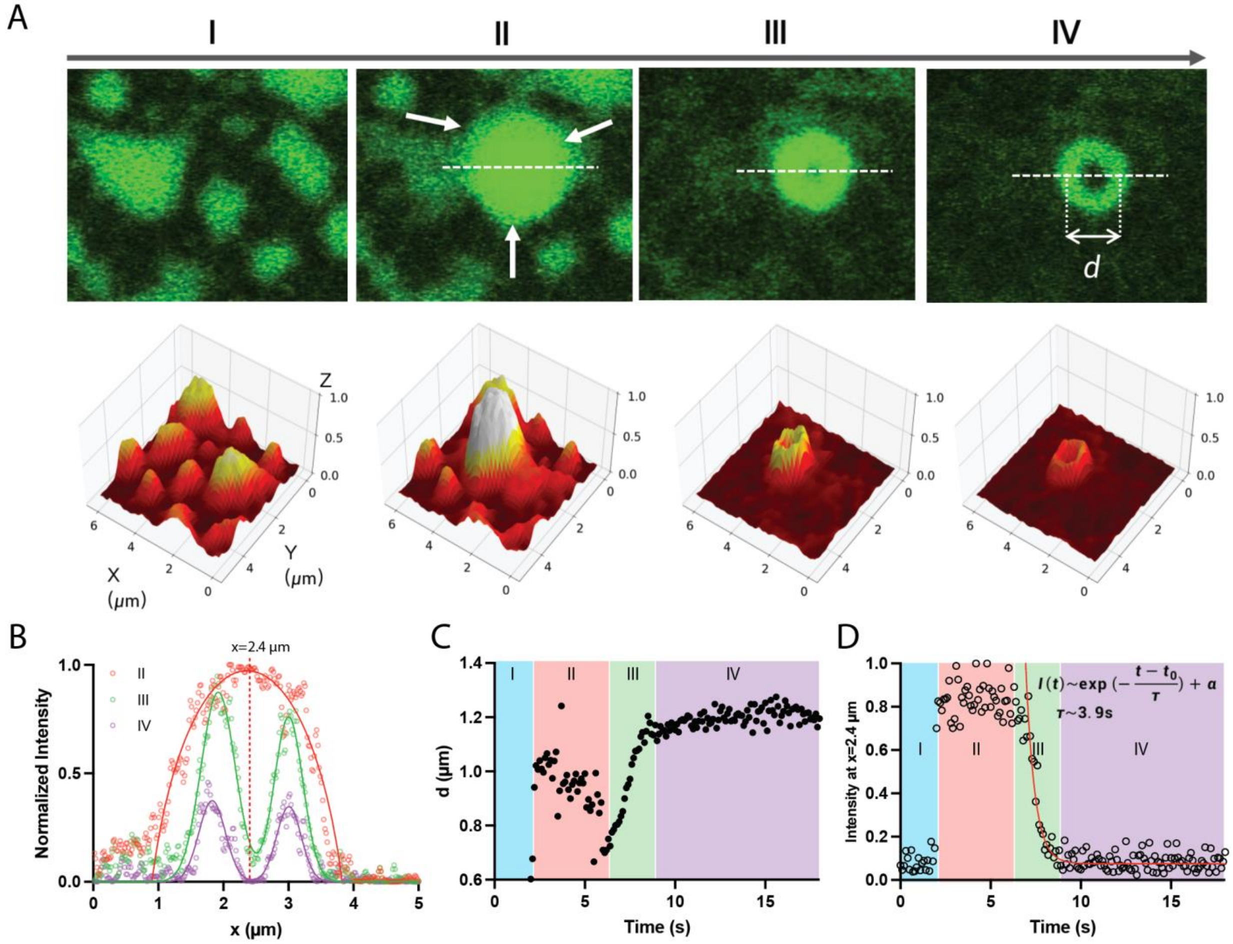

Figure S33. (A) Image sequences from the formation of liquid droplet (stage I and II) to torus (stage III and IV). The bottom panels are the 3D intensity profiles of the upper panels. (B) Intensity profile at stage II, III, and IV along the white dashed line in A. The profile in the stage II is fitted by circular function, which was better fitting than a Gaussian distribution and the others are fitted with bimodal Gaussian distribution. (C) The size of the hole, d, is rapidly saturated near 1.2 μm, consistently with the decaying of the intensity at x = 2.4 μm. (D) The intensity at x = 2.4 μm decays exponentially and the fitted lifetime (τ) is about 3.9 s.

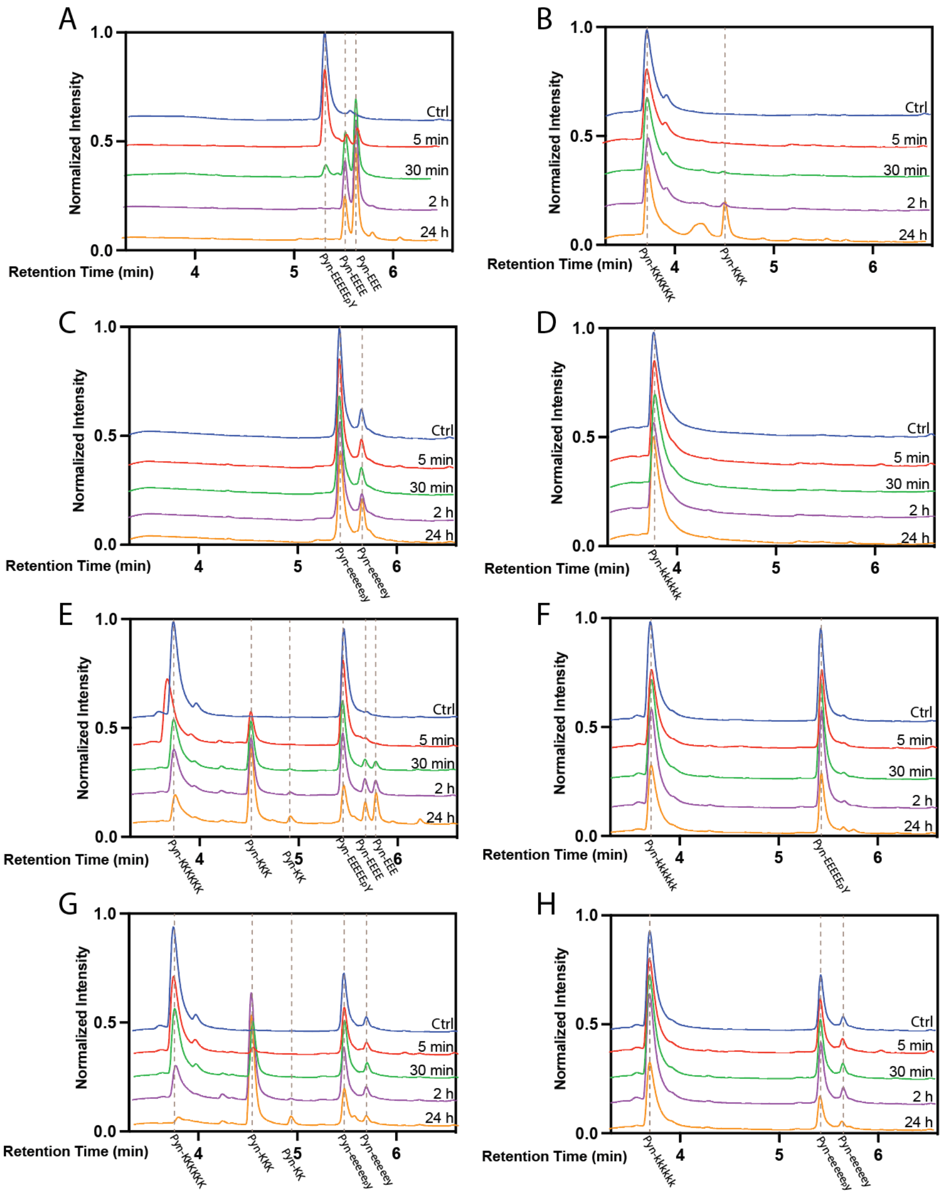

Figure S34. Normalized LC graph for (A) Pyn-(L)-EEEEE$_{p}$Y (**1**), (B) Pyn-(L)-KKKKKK (**4**), (C) Pyn-(D)-eeeee$_{p}$y (**3**), (D) Pyn-(D)-kkkkkk (**2**), (E) **1** mixed with 2 equivalences of **4** (**1/4**), (F) **1** mixed with 2 equivalences of **2** (**1/2**), (G) **3** mixed with 2 equivalences of **4** (**3/4**), and (H) **3** mixed with 2 equivalences of **2** (**3/2**) treated with 1 mg/mL proteinase K for 5 min, 30 min, 2 h, and 24 h at 37 ˚C in water.

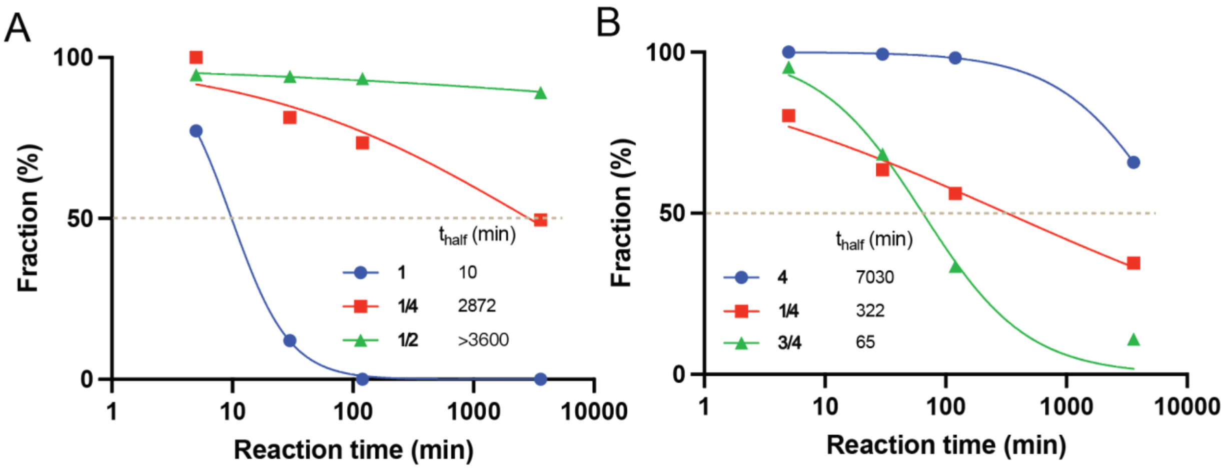

Figure S35. Time-dependent substrate depletion by 1 mg/mL Proteinase K for (A) Pyn-(L)-EEEEE$_p$Y (**1**) and (B) Pyn-(L)-KKKKKK (**4**). Legends indicate different reaction conditions: **1** (Pyn-(L)-EEEEE$_p$Y), **4** (Pyn-(L)-KKKKKK), **1/4**: 500 μM Pyn-(L)-EEEEE$_p$Y (**1**) mixed with 2 equivalents of Pyn-(L)-KKKKKK (**4**); **1/2**: 500 μM Pyn-(L)-EEEEE$_p$Y (**1**) mixed with 2 equivalents of Pyn-(D)-kkkkkk (**2**); **3/4**: 500 μM Pyn-(D)-eeeee$_p$y (**3**) mixed with 2 equivalents of Pyn-(L)-KKKKKK (**4**).

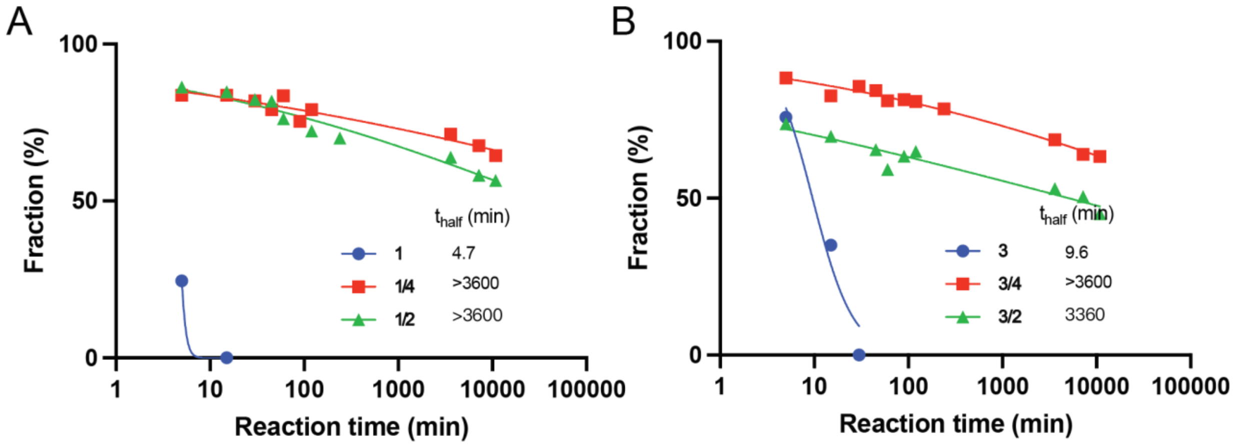

Figure S36. Time-dependent substrate depletion by 1U/mL ALP for (A) Pyn-(L)-EEEEE$_p$Y (**1**) and (B) Pyn-(D)-eeeee$_p$y (**3**). Legends indicate different reaction

conditions: **1** (Pyn-(L)-EEEEE$_p$Y), **3** (Pyn-(D)-eeeee$_p$y), **1/4**: 500 μM Pyn-(L)-EEEEE$_p$Y (**1**) mixed with 2 equivalents of Pyn-(L)-KKKKKK (**4**); **1/2**: 500 μM Pyn-(L)-EEEEE$_p$Y (**1**) mixed with 2 equivalents of Pyn-(D)-kkkkkk (**2**); **3/4**: 500 μM Pyn-(D)-eeeee$_p$y (**3**) mixed with 2 equivalents of Pyn-(L)-KKKKKK (**4**). **3/2**: Pyn-(D)-eeeee$_p$y (**3**) mixed with 2 equivalents of Pyn-(D)-kkkkkk (**2**).

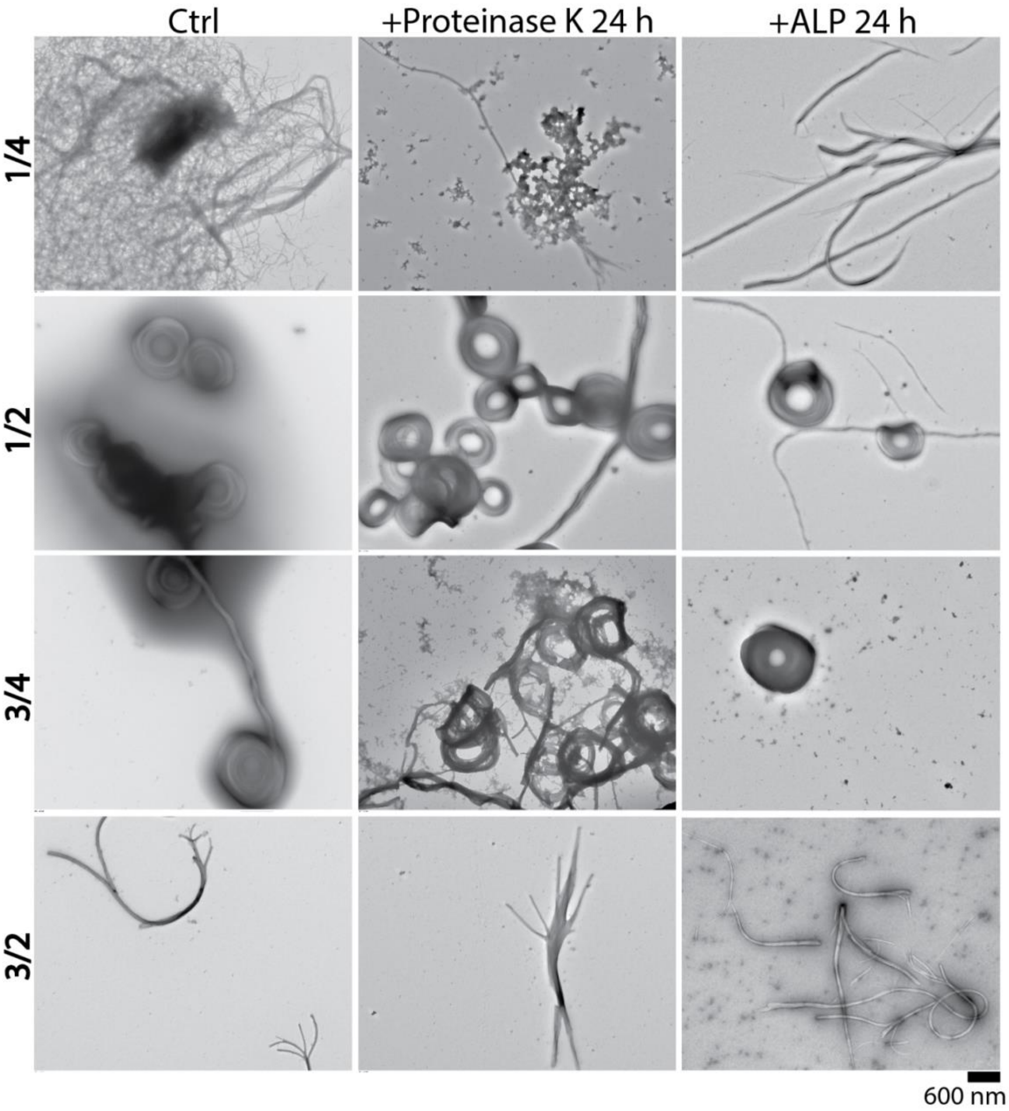

Figure S37. TEM images of mixtures **1/4, 1/2, 3/4,** and **3/2** treated with 1 mg/mL proteinase K or 1 U/mL ALP at 37 °C and neutral pH for 24 h. **1/4**: 500 μM Pyn-(L)-

EEEEE$_{p}$Y (**1**) mixed with 2 equivalents of Pyn-(L)-KKKKKK (**4**); **1/2**: 500 μM Pyn-(L)-EEEEE$_{p}$Y (**1**) mixed with 2 equivalents of Pyn-(D)-kkkkkk (**2**); **3/4**: 500 μM Pyn-(D)-eeeee$_{p}$y (**3**) mixed with 2 equivalents of Pyn-(L)-KKKKKK (**4**); **3/2**: 500 μM Pyn-(D)-eeeee$_{p}$y (**3**) mixed with 2 equivalents of Pyn-(D)-kkkkkk (**2**).

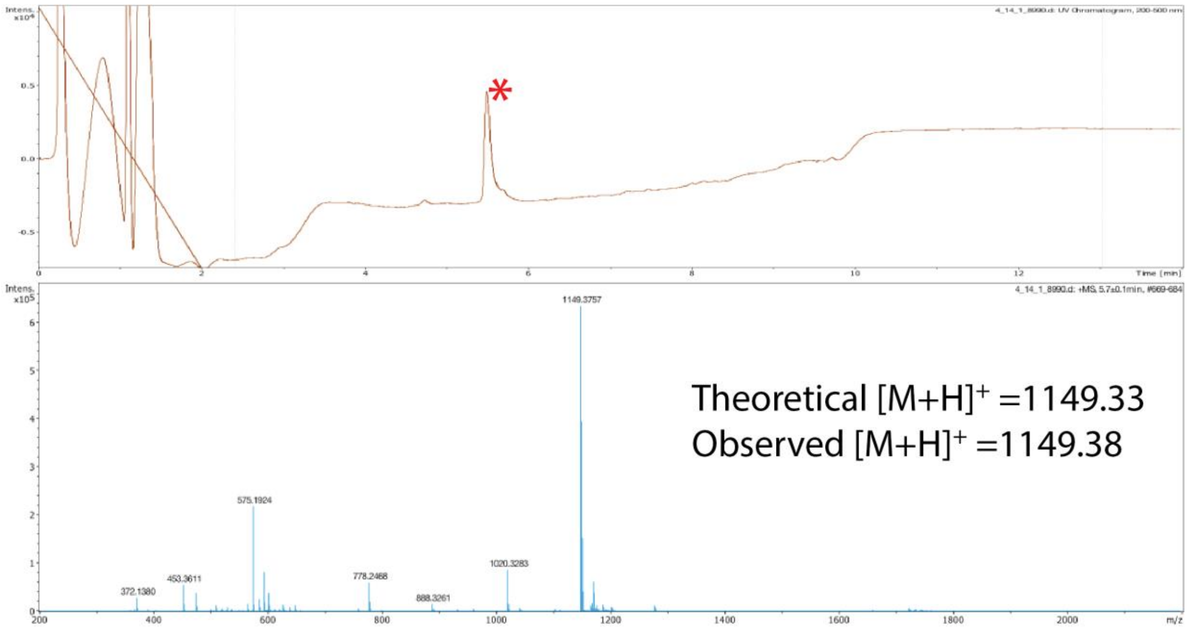

Figure S38. LC (up) and MS (down) spectra of Pyn-(L)-EEEEE$_{p}$Y (**1**). The LC peak corresponding to the assigned MS spectrum is marked with a red asterisk.

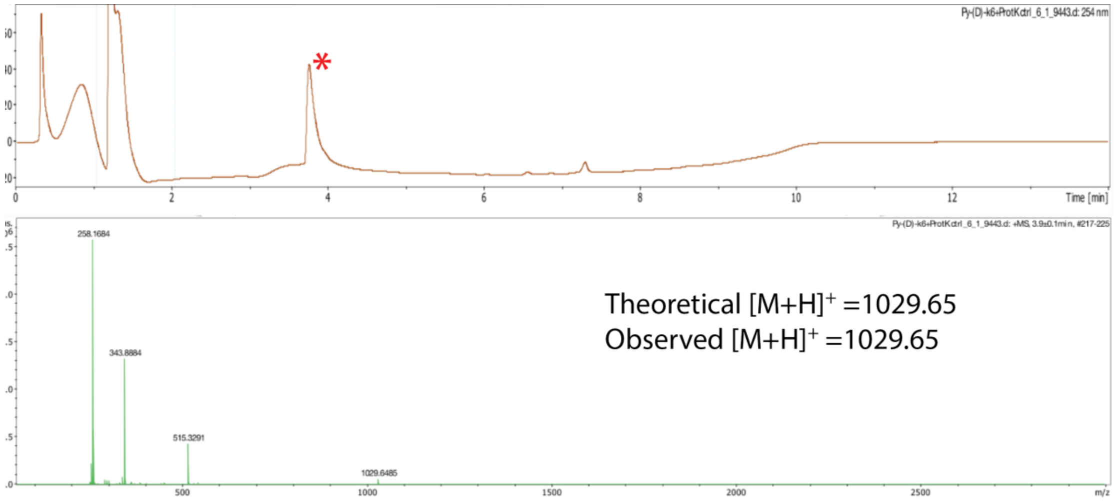

Figure S39. LC (up) and MS (down) spectra of Pyn-(D)-kkkkkk (**2**).The LC peak corresponding to the assigned MS spectrum is marked with a red asterisk.

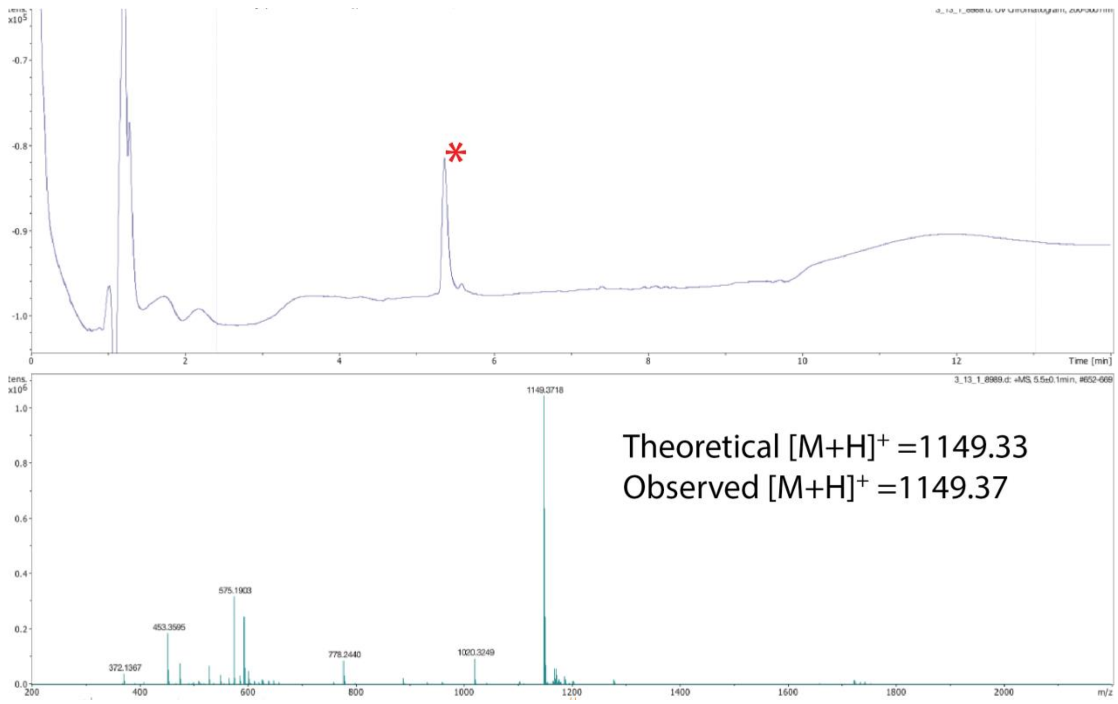

Figure S40. LC (up) and MS (down) spectra of Pyn-(D)-eeeee$_p$y (**3**). The LC peak corresponding to the assigned MS spectrum is marked with a red asterisk.

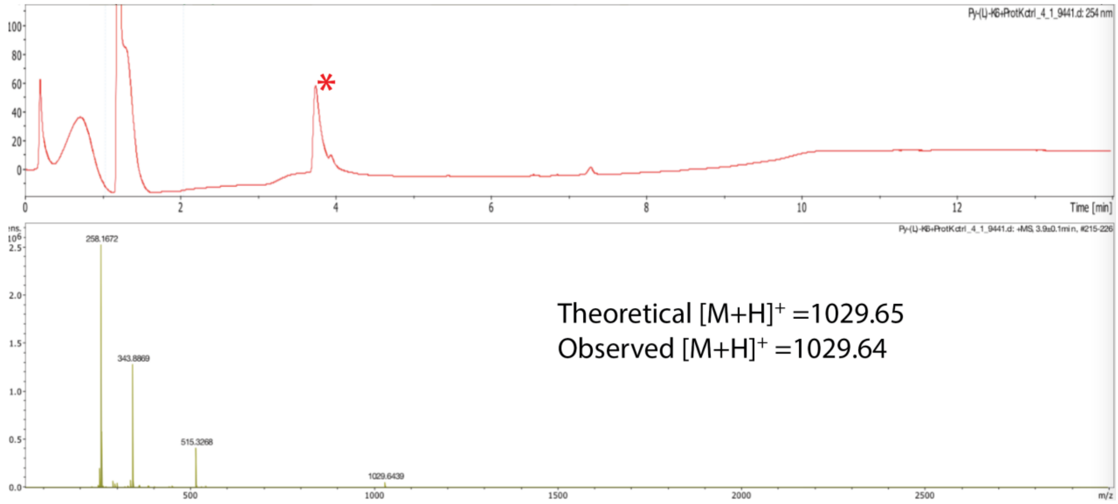

Figure S41. LC (up) and MS (down) spectra of Pyn-(L)-KKKKKK (**4**). The LC peak corresponding to the assigned MS spectrum is marked with a red asterisk.

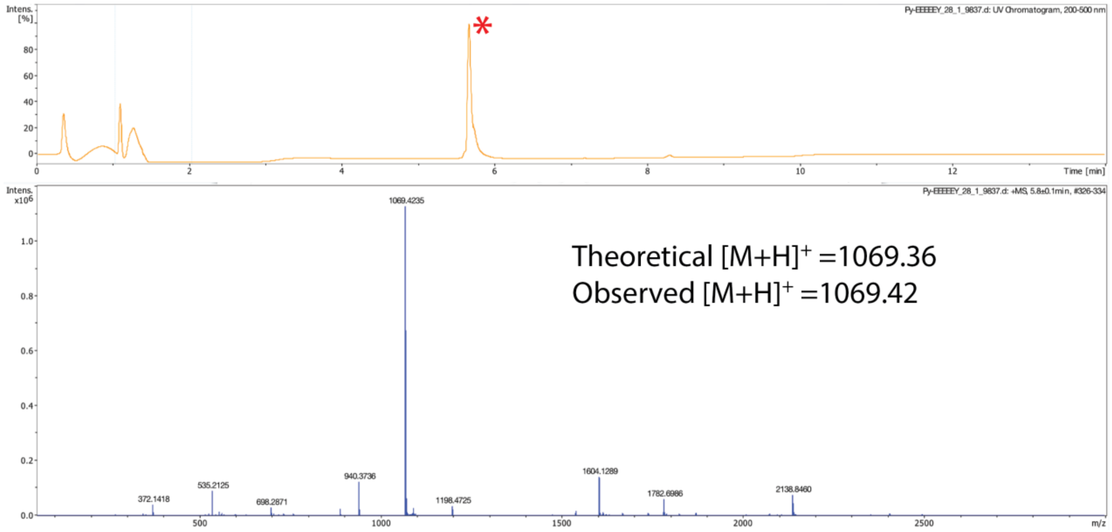

Figure S42. LC (up) and MS (down) spectra of Pyn-(L)-EEEEEY (**5**). The LC peak corresponding to the assigned MS spectrum is marked with a red asterisk.

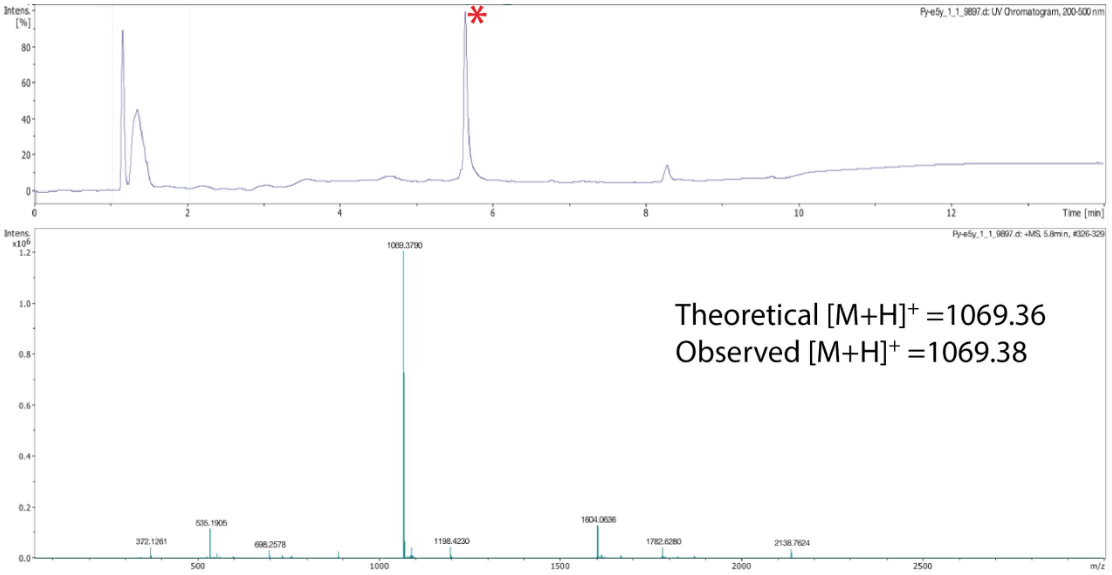

Figure S43. LC (up) and MS (down) spectra of Pyn-(D)-eeeeey (**6**). The LC peak corresponding to the assigned MS spectrum is marked with a red asterisk.

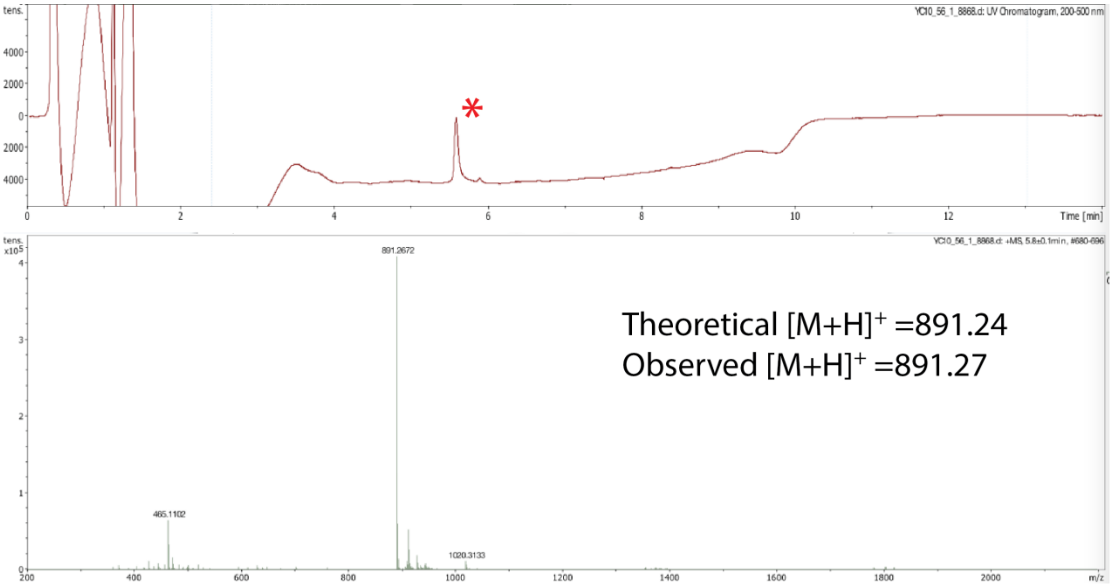

Figure S44. LC (up) and MS (down) spectra of Pyn-(L)-EEE$_p$Y (**7**). The LC peak corresponding to the assigned MS spectrum is marked with a red asterisk.

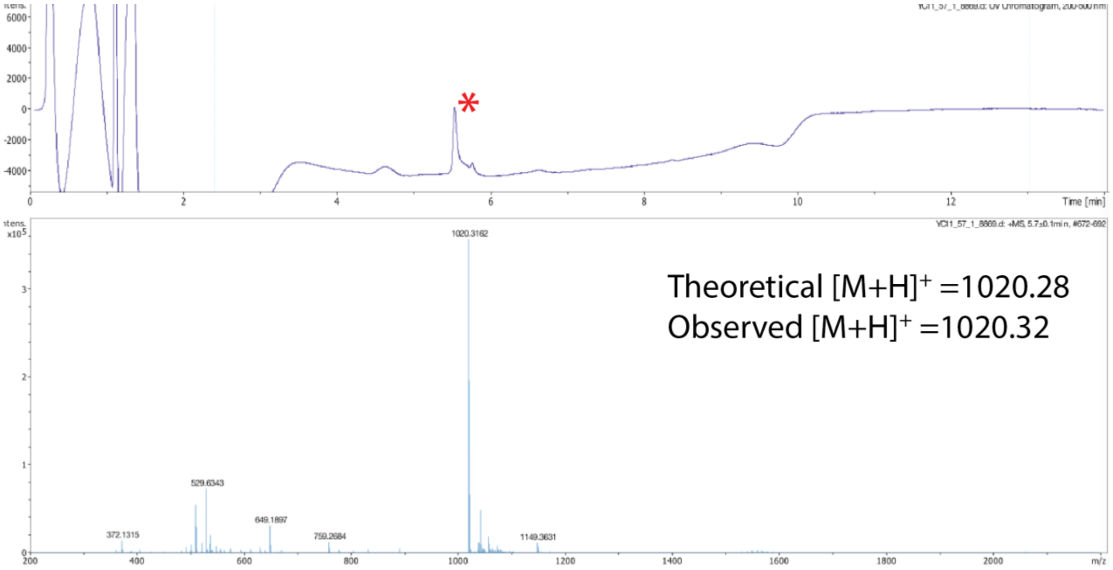

Figure S45. LC (up) and MS (down) spectra of Pyn-(L)-EEEE$_p$Y (**8**). The LC peak corresponding to the assigned MS spectrum is marked with a red asterisk.

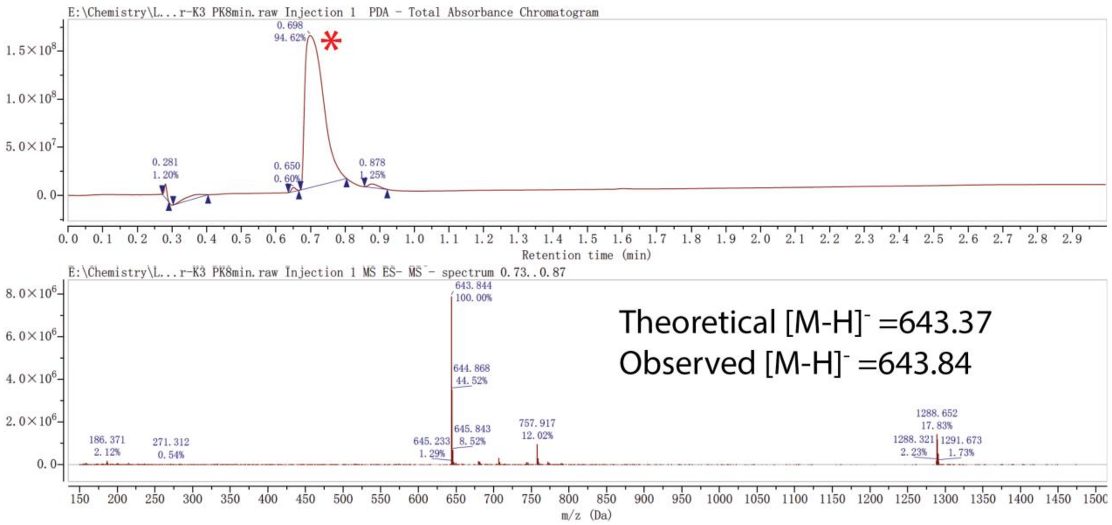

Figure S46. LC (up) and MS (down) spectra of Pyn-(L)-KKK (**9**). The LC peak corresponding to the assigned MS spectrum is marked with a red asterisk.

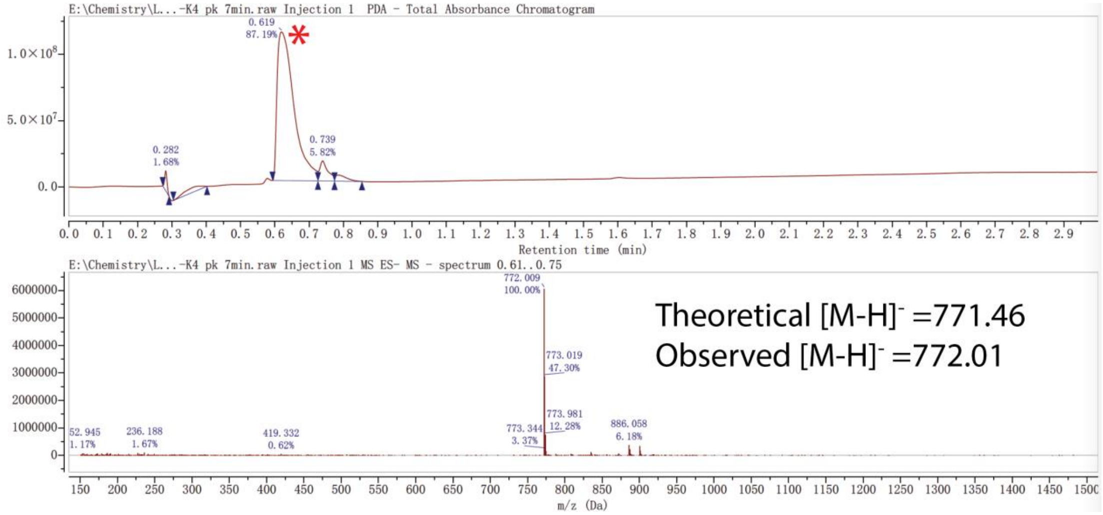

Figure S47. LC (up) and MS (down) spectra of Pyn-(L)-KKKK (**10**). The LC peak corresponding to the assigned MS spectrum is marked with a red asterisk.

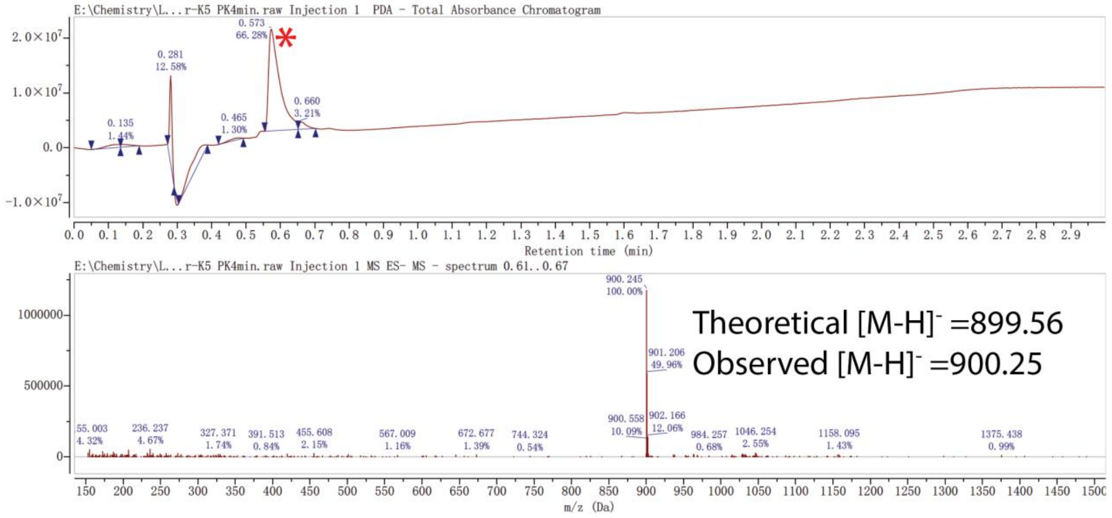

Figure S48. LC (up) and MS (down) spectra of Pyn-(L)-KKKKK (**11**). The LC peak corresponding to the assigned MS spectrum is marked with a red asterisk.

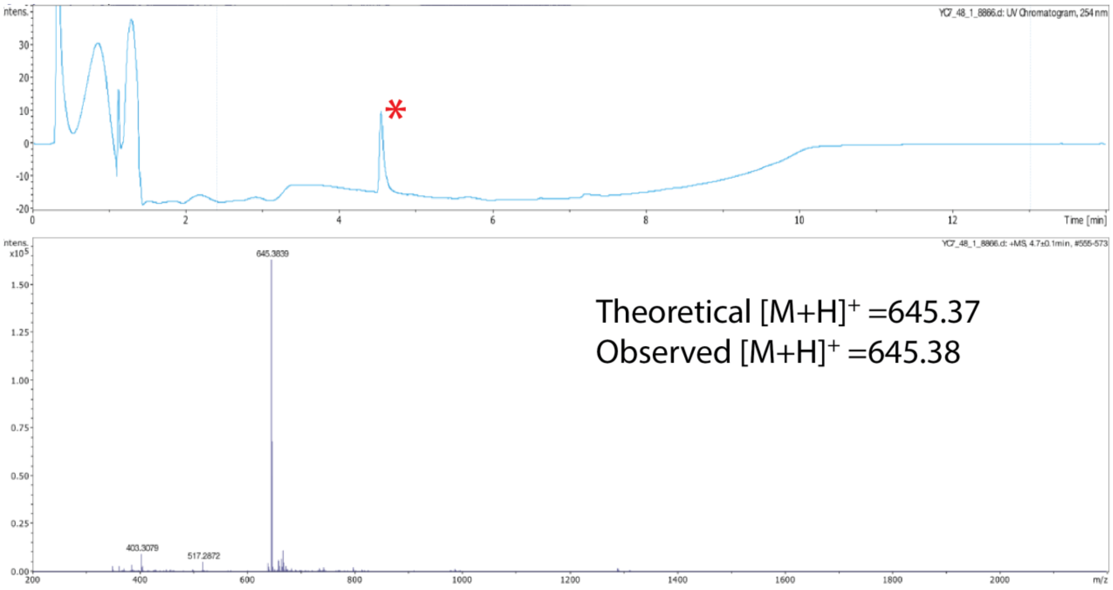

Figure S49. LC (up) and MS (down) spectra of Pyn-(D)-kkk (**12**). The LC peak corresponding to the assigned MS spectrum is marked with a red asterisk.

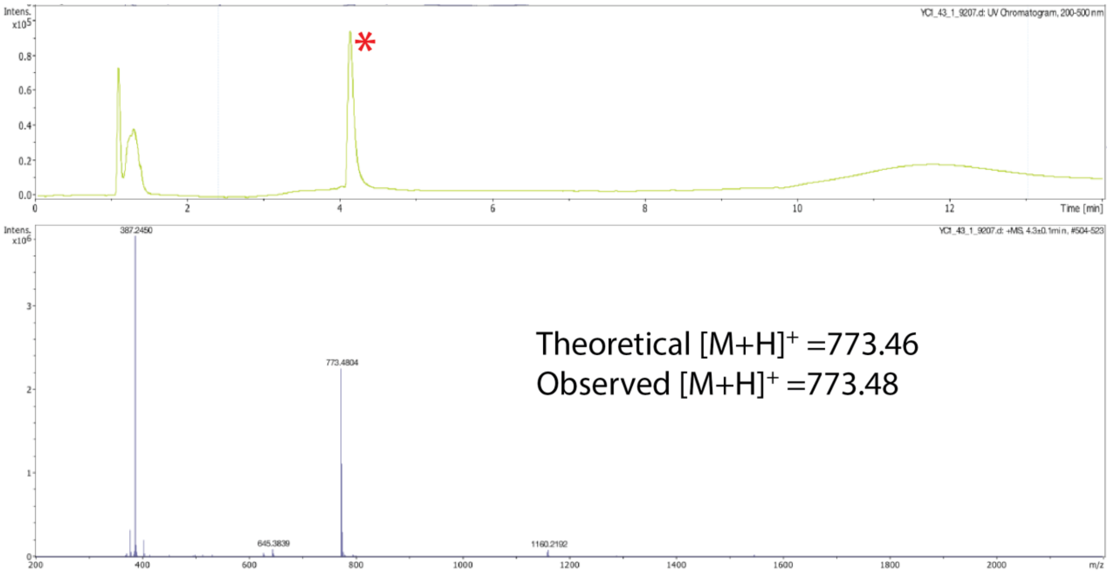

Figure S50. LC (up) and MS (down) spectra of Pyn-(D)-kkkk (**13**). The LC peak corresponding to the assigned MS spectrum is marked with a red asterisk.

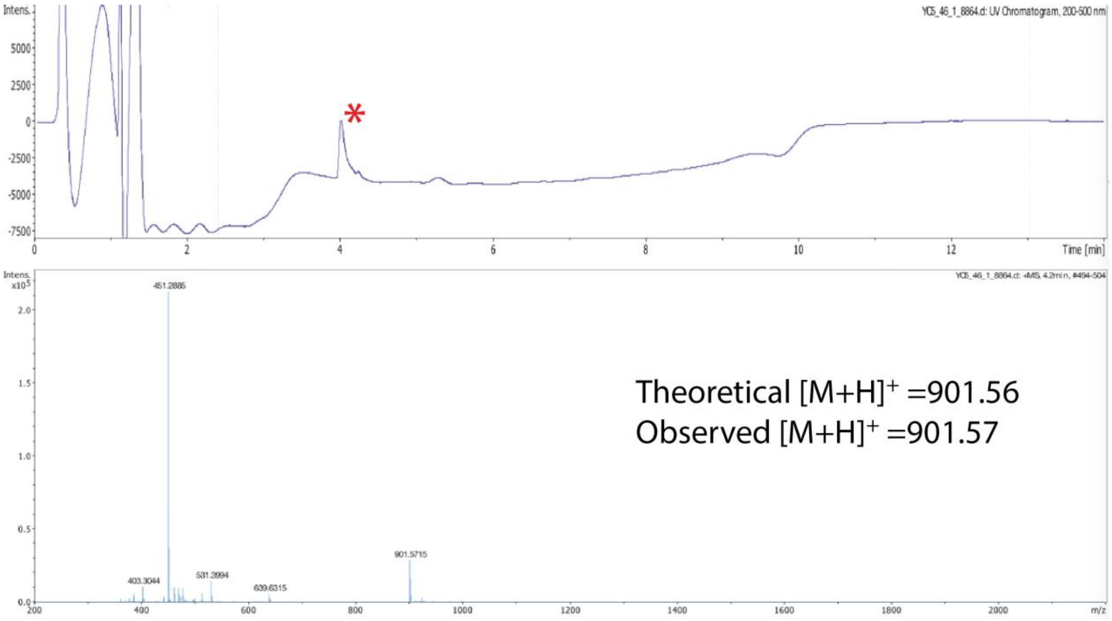

Figure S51. LC (up) and MS (down) spectra of Pyn-(D)-kkkkk (**14**). The LC peak corresponding to the assigned MS spectrum is marked with a red asterisk.

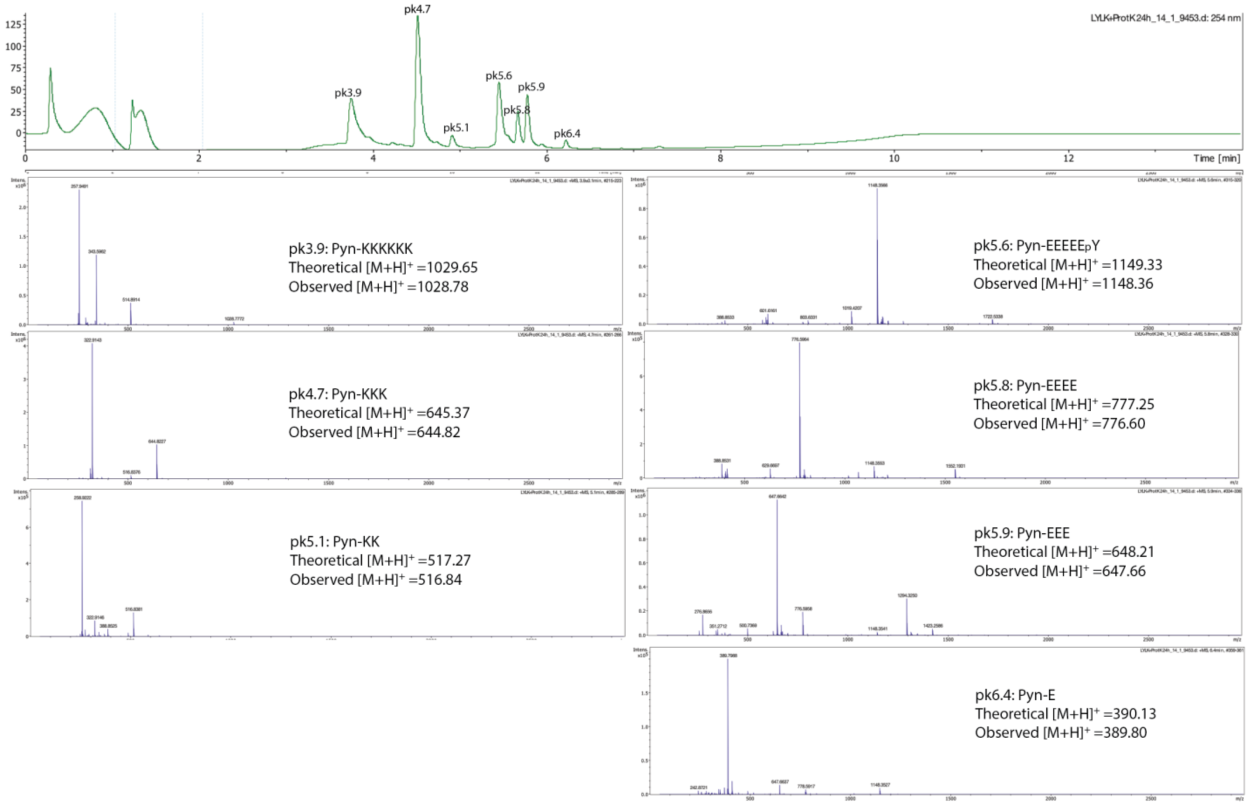

Figure S52. LC/MS spectra of **1/4** treated with 1mg/mL proteinase K after 24 h.

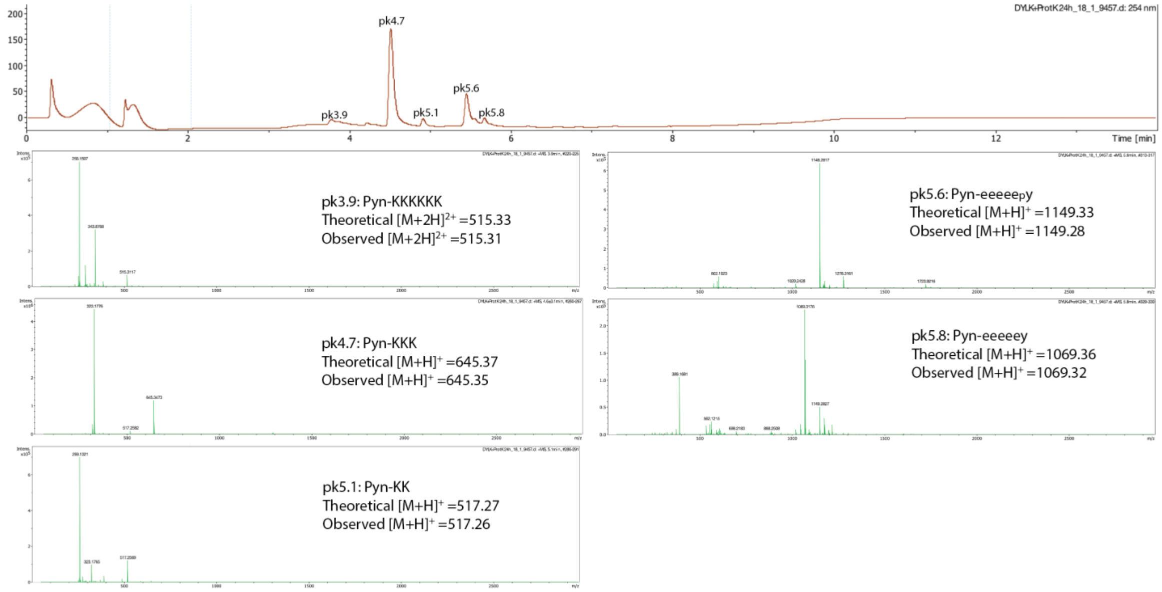

Figure S53. LC/MS spectra of **3/4** treated with 1mg/mL proteinase K after 24 h.

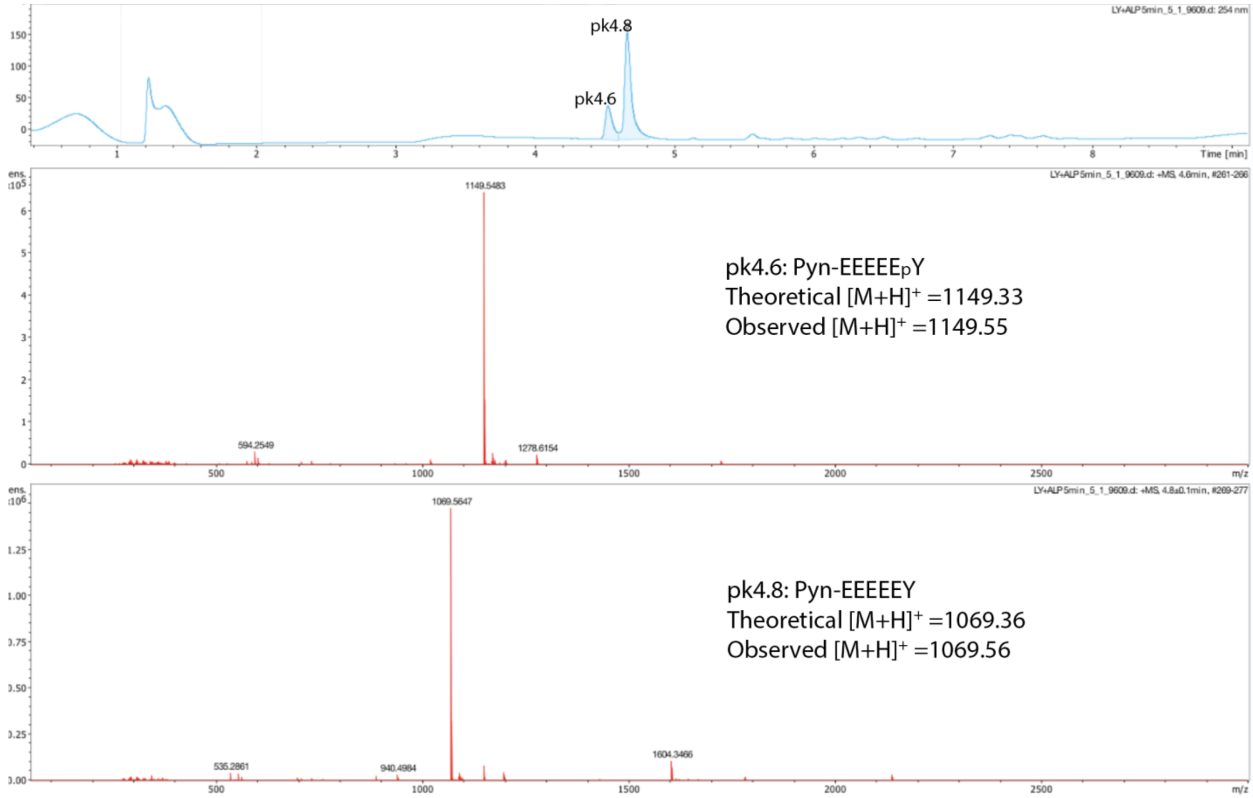

Figure S54. LC/MS spectra of **1** treated with 1 U/mL ALP after 5 min.

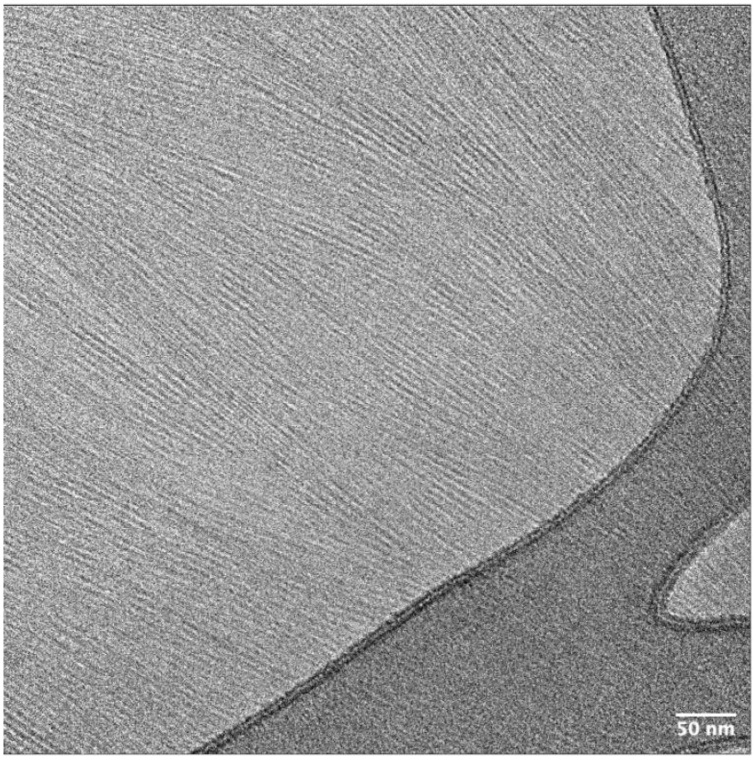

Figure S55. Cryo-EM image of **1/2** mixture.

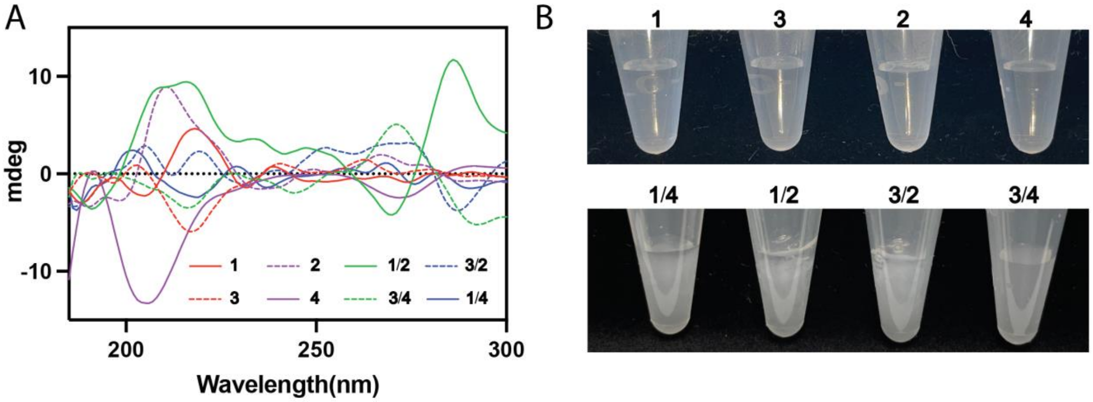

Figure S56. (A) CD spectra and (B) photographs of peptides 1–4 and heterotypic mixtures. **1/2: 1** mixed with 2 equivalences of **2**; **1/4: 1** mixed with 2 equivalences of **4**; **3/2: 3** mixed with 2 equivalents of **2.** and **3/4: 3** mixed with 2 equivalents of **4**. Enantiomeric relationships are indicated by solid versus dashed lines in panel A.

Table S1. Integrated LC peak areas for samples treated with proteinase K.

Sample	Time	pk3.9	pk4.4	pk4.6	pk5.1	pk5.6	pk5.8	pk5.9	pk6.4

s	points								
**1**	5min					504.9	60.74	88.25	
	30min					78.06	195.8	376.5	
	2h						214.3	453	
	24h						196.6	492.7	6.787
**4**	5min	433.4							
	30min	442.6		2.675					
	2h	412.4		7.49					
	24h	353.7	94.6	89.05					
**1/4**	5min	1031		253.1		986.6			
	30min	710.1		400.9	7.023	666.5	80.36	71.89	
	2h	626.7		477	13.59	575	107.8	99.97	
	24h	379.9		676.7	41.99	367.1	135.5	217.7	21.06
**1/2**	5min	968.8				710.4	31.78	8.51	
	30min	1227				889.8	38.63	17.21	
	2h	1214				880.7	44.45	17.37	
	24h	825.5				513.6	37.99	24.8	
**3/4**	5min	1021	9.398	39.72		375.9	95.81		
	30min	887.1	13.74	396.4		453.7	119		
	2h	449.1	28.06	854.1	9.118	428.3	112.6		
	24h	116.1	20.25	871.3	51.58	311.4	76.08		

Table S2. Integrated LC peak areas for samples treated with ALP.

Samples	Time points	pk4.5	pk4.7
**1**	ctrl	883.06	0
	5min	197.69	606.88
	15min	0	839.76
**3**	ctrl	1124.1	0
	5min	907.63	290.01
	15min	404.69	751.51
	30min	0	1198.3
**1/4**	ctrl	1451.3	0
	5min	685.15	133.04
	15min	767.94	149.23
	30min	605.66	134.17
	45min	675.43	177.83
	60min	664.32	131.02
	90min	228.2	74.404
	120min	583.55	153.93
	1day	441.55	177.92
	2day	393.66	188.73
	3day	350.3	193.03
**1/2**	ctrl	1057.9	0
	5min	698.08	110.56
	15min	644.32	115.8
	30min	670.07	143.99
	45min	639.06	142.64
	60min	633.71	198.23
	120min	529.98	202.76

	240min	631.68	270.12
	1day	442.08	248.85
	2day	437.2	313.81
	3day	326.57	251.06
**3/4**	ctrl	665.84	0
	5min	837.57	110.86
	15min	763.09	160.08
	30min	871.38	146.53
	45min	866.23	161.79
	60min	864.91	202.88
	90min	742.73	170.08
	120min	854.71	202.68
	240min	1048.38	287.58
	1day	682.86	311.74
	2day	602.86	339.54
	3day	737.74	428.3
**3/2**	ctrl	1134.9	0
	5min	846.09	301.17
	15min	796.86	346.2
	45min	693.47	366.26
	60min	385.32	266.49
	90min	660.31	381.44
	120min	716.98	388.06
	1day	430.35	381.56
	2day	378.71	372.44
	3day	363.97	444.42
